# Performance of DeepSeek V3, DeepSeek R1, ChatGPT 4o, and ChatGPT o1 on the National Health Professional and Technical Qualification Examination (Intermediate Level) in China: Comparative Analysis

**DOI:** 10.2196/90673

**Published:** 2026-04-06

**Authors:** Jipeng Xue, Shitong Wang, Jinan Yang, Xiaogang Guo, Jie Shen, Qiwen Wang

**Affiliations:** 1 First Affiliated Hospital Zhejiang University Hangzhou, Zhejiang China; 2 Hangzhou City University Hangzhou, Zhejiang China

**Keywords:** large language models, DeepSeek, ChatGPT, support clinical decision-making, cardiology

## Abstract

**Background:**

In recent years, large language models (LLMs) have undergone swift cycles of refinement and iteration. However, in the realm of clinical medicine, different LLMs' capability of logical reasoning and disease diagnosis needs further investigation.

**Objective:**

The aim of our study was to evaluate the performance of 4 different LLMs in the National Health Professional and Technical Qualification Examination in China.

**Methods:**

A total of 398 multiple-choice questions of 5 different question types were integrated within the examination with respect to the diagnosis or care of cases. These questions were categorized into different cardiology subspecialties and different clinical disciplines. DeepSeek V3 and R1 were accessed through an application programming interface, while ChatGPT 4o and o1 were queried via its public chat-based interface. We offered the same prompts instructing LLMs to assume the role of a physician and provide answers with explanations at the beginning of each conversation. We assessed different LLMs’ performance by the accuracy in the responses to the multiple-choice questions. For the first 3 examination sections, McNemar test was used to compare the accuracy among the models, with post hoc pairwise comparisons performed using partitions of chi-square method and Bonferroni correction (significance set at *P*<.008). For the fourth section involving partially credit scoring, one-way ANOVA was performed to compare the mean scores among the models, with statistical significance set at *P*<.05.

**Results:**

Both DeepSeek V3 and R1 showed superior performance in the first 3 sections of the Chinese National Health Professional and Technical Qualification Examination, achieving an overall performance of 93% and 93.6%, respectively. ChatGPT 4o and o1 achieved accuracies of 73.3% and 69%, respectively (all *P*<.001 compared with DeepSeek V3). For the fourth section, the performance of all 4 LLMs markedly declined compared to their results in the preceding sections. Particularly, in the section of gastroenterology and hematology, DeepSeek V3 achieved the highest accuracy, while R1 ranked first in cardiology and neurology. ChatGPT o1 achieved the highest accuracy in the topic of coronary artery disease, with no statistical significance.

**Conclusions:**

DeepSeek V3 and R1 showed remarkable potential in facilitating clinical decision-making in the Chinese professional examination, with both outperforming ChatGPT 4o and o1. Nonetheless, future research should continue evaluating their economic efficiency and susceptibility to hallucination.

## Introduction

In recent years, large language models (LLMs) such as Anthropic 2024, Google Gemini 2024 [1], and OpenAI 2024 [2] have undergone swift refinement and iteration. Since their launch by OpenAI, ChatGPT holds significant potential across various facets of the medical field, including medical documentation, scientific writing, and medical education [3-5]. Numerous studies have demonstrated its potential applications in health care, particularly in cardiology, due to its ability to advance the management of long-term heart conditions [6], provide medical advice on acute cardiac events [7], answer clinical cardiac questions [8], interpret cardiac diagnostics tests [9], and design an individualized therapeutic strategy [10]. Specifically, Wang et al [11] revealed that ChatGPT was proficient in specific medical tasks such as discharge summarization and group learning within the Chinese linguistic paradigm. Another investigation showed that both ChatGPT-3.5 and GPT-4 can successfully achieve average scores that exceed the admission benchmark on the master’s degree entrance examination in clinical medicine [12] only, with an accuracy of 48% and 68% respectively. However, Sarangi et al [13] illustrated that ChatGPT-4 has limitations in processing radiology anatomy. While these studies suggest that ChatGPT may have potential proficiency in logical reasoning and disease diagnosis, considering its financial cost and underperformance on image-based questions, its performance warrants further evaluation.

In addition to proprietary systems, open-source frameworks are achieving substantial breakthroughs in capability development, actively narrowing the performance divide with their closed-source counterparts such as the newly published DeepSeek MoE. Launched in January 2025, DeepSeek’s DeepThink (R1), an open-source LLM [14], is different from proprietary models as it fosters a sustained learning environment by integrating publicly accessible open-source datasets, which may in turn improve its ability to adapt to the continuously evolving domains of medical expertise and scientific analysis [15,16]. Moreover, compared to proprietary LLMs, DeepSeek R1 offered free-tier access and reduced financial costs, making artificial intelligence more accessible for smaller institutions [17-20]. In terms of performance on mathematics and science problems, DeepSeek-R1 demonstrates proficiency rivaling that of the ChatGPT-o1 model, released in September by OpenAI, whose reasoning models were considered industry leaders [21]. However, in the clinical medicine domain, DeepSeek-R1’s capabilities of logical reasoning and disease diagnosis warrants further investigation.

The National Health Professional and Technical Qualification Examination (intermediate level) in China is a government-organized assessment, and passing the examination demonstrates the requisite competence to assume corresponding levels of professional and technical responsibilities. This examination is designed to evaluate the clinical acumen, depth of knowledge, diagnostic competence, and clinical decision-making expertise of resident physicians who have chosen cardiology as their practice area and are aiming at the promotion to fellows. The examination consists of 5 types of questions, namely, A1 (knowledge-based multiple choice), A2 (case-based multiple choice), A3/A4 (case-group-based multiple choice), B (matching), and X (multiple choice), totaling 398 multiple-choice questions (MCQs) distributed across 4 parts: basic concepts, relevant expertise, foundational professional knowledge, and professional practical skills. In the sections of basic concepts and relevant expertise, the examination encompasses several distinct branches of internal medicine covering the foundational concepts and standard clinical practices in medicine, such as respiratory medicine, cardiology, gastroenterology, hematology, nephrology, infectious diseases, neurology, rheumatology and immunology, endocrinology, and emergency medicine. In the section on foundational professional knowledge, a comprehensive overview of 10 prevalent cardiac conditions is provided, covering in-depth, cardiology-specific expertise in heart failure, arrhythmias, cardiac arrest and sudden cardiac death, congenital cardiovascular diseases, hypertension, coronary artery disease, valvular heart disease, infective endocarditis, myocardial diseases, and pericardial disorders. For the section on professional practical skills, a series of multiple-answer questions is set within a simulated clinical scenario. To eliminate the need for analyzing pictures or other visual forms, we established a dataset comprising questions and options only in text format.

In this study, we aimed to investigate whether different types or fields of questions would influence LLMs’ performance in the Chinese linguistic paradigm. Delving deeper, we aimed to evaluate the efficacy and reliability of different LLMs’ decision-making ability, offering insights and practical recommendations of their possible role in facilitating clinical decision-making. We selected the most recent LLMs, GPT-4o and GPT-o1 in the GPT family of models, as they represent the proprietary systems that were released in May 2024 and September 2024, respectively. In contrast to GPT’s proprietary framework, we selected the open-access models DeepSeek V3 and R1 [14,22] launched in January 2025 to compare their accuracy, robustness, and limitations.

## Methods

### Chinese National Health Professional and Technical Qualification Examination (Intermediate Level) Knowledge Datasets

**Figure 1:**
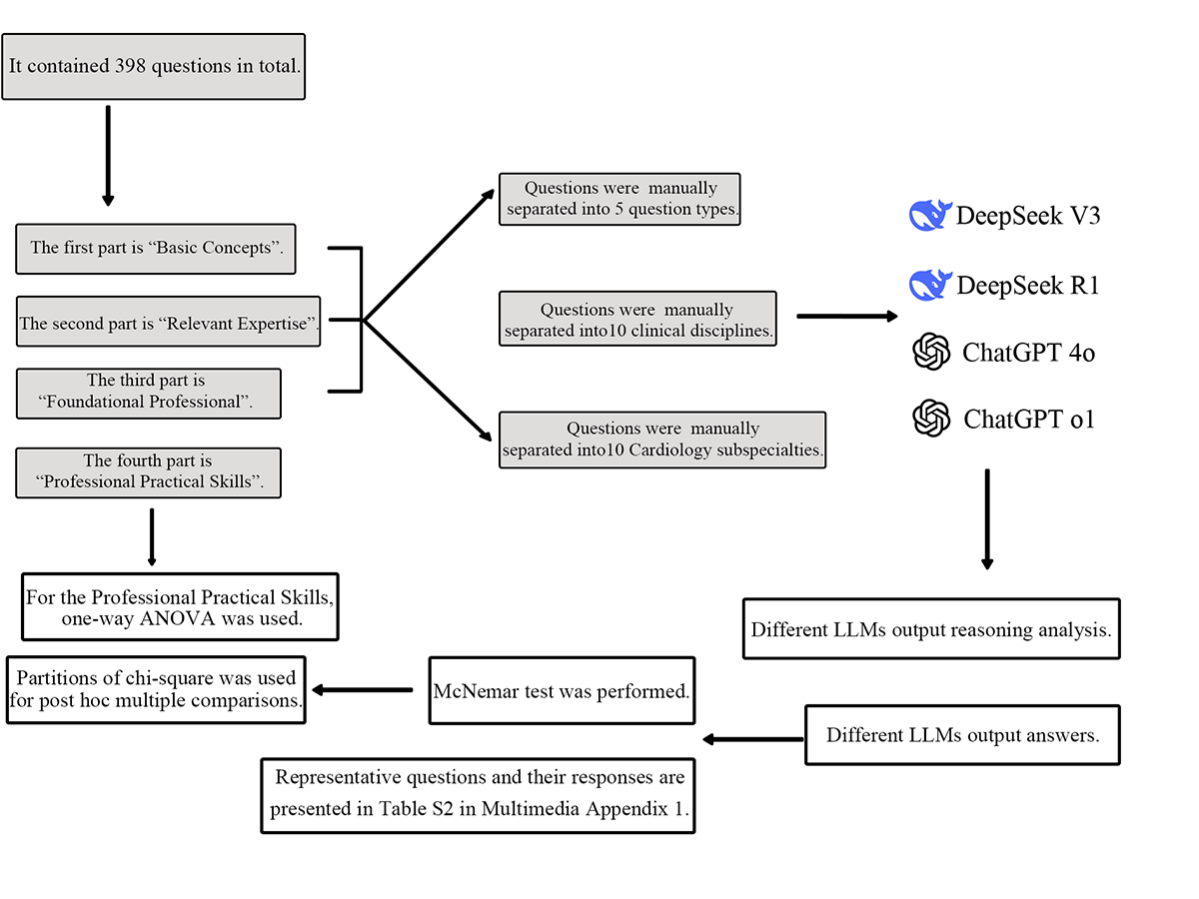
Workflow illustration of this study.

We created an examination dataset of the National Health Professional and Technical Qualification Examination (intermediate level) containing questions extracted from the book *Cardiovascular Medicine: Synchronized Exercises and Comprehensive Mock Examinations* [23] to test the performance of different LLMs (Figure 1). We randomly selected 398 queries from the dataset across various medical fields (Multimedia Appendix 1) under 4 different types: A1 (knowledge-based multiple choice), A2 (case-based multiple choice), A3/A4 (case-group-based multiple choice), B (matching), and X (multiple choice). The composition of these four sections is presented in Table 1. Except for the X-type questions, each question presented 5 answer options, with only 1 correct answer. Meanwhile, for the X-type questions, there were 5-11 options across various questions. All questions were composed and presented in Chinese, with no English inclusion or explanation.

**Table 1:** Dataset of the National Health Professional and Technical Qualification Examination in China.

		Questions, n
**Parts of the examination (N=398)**		
	Basic Concepts	100
	Relevant Expertise	100
	Foundational Professional Knowledge	100
	Professional Practical Skills	98
**Format of the examination (N=398)**		
	A1-type	89
	A2-type	78
	A3/A4-type	88
	B-type	45
	X-type	98
**Clinical disciplines in the section of Basic Concepts and Relevant Expertise (n=200)**		
	Respiratory Medicine	34
	Cardiology	32
	Gastroenterology	24
	Hematology	17
	Nephrology	10
	Infectious diseases	31
	Neurology	19
	Rheumatology and immunology	8
	Endocrinology	15
	Emergency medicine	10
**Cardiology subspecialties in the section of Foundational Professional Knowledge (n=100)**		
	Heart failure	10
	Arrhythmias	25
	Cardiac arrest and sudden cardiac death	1
	Congenital cardiovascular diseases	1
	Hypertension	12
	Coronary artery disease	17
	Valvular heart disease	3
	Infective endocarditis	3
	Myocardial diseases	15
	Pericardial disorders	13

### LLM Testing

In this comparative study, we tested 398 MCQs selected from the dataset with 4 different LLMs, namely, DeepSeek V3, DeepSeek R1, ChatGPT 4o, and ChatGPT o1 by manually entering the questions. To assess the performance of DeepSeek V3 and R1, we used the application programming interface (API) provided by SiliconFlow [24], a cloud service platform, due to usage limitations on the official server. For the evaluation of ChatGPT 4o and o1, we obtained them through the official chat user interface (UI). Temperature settings are crucial in the usage of LLMs, as this directly influences the randomness of the generated content. In this study, the temperature for DeepSeek V3 and R1 was set typically at 0.7. Regarding the ChatGPT chat UI, we were unable to find the direct control over temperature settings; thus, these 2 models were evaluated under default configurations, which could have introduced variability in systematic bias. The responses were generated by different LLMs between February 21 and February 28, 2025.

Questions were run independently without additional instructions during the conversation. To enhance contextual connections capabilities, we offered the same prompts instructing LLMs to assume the role of a physician and provide answers with explanations at the beginning of each conversation [25,26] (Table S1 of Multimedia Appendix 1). Answers and explanations generated by LLMs were meticulously documented using Word and cross-referenced with the correct answers to ensure precise evaluation of examination performance.

For the first three parts (basic concepts, relevant expertise, foundational professional knowledge), we evaluated different models’ performance by calculating the accuracy rates (percentage of correct answers out of the total). For the final part of professional practical skills, each question was assigned one point; correct answers received full credit, while incorrect ones received none. For partially correct responses, scores were awarded proportionally based on the number of accurate options selected. To compare the performance of different models in various types or fields of questions, the questions from the first three sections were categorized into different segments based on their types and subject domains, as mentioned before, and then analyzed.

### Cost-Effectiveness Analysis

For DeepSeek V3 and R1, costs were calculated on a pay‑per‑token basis using the official pricing published on the SiliconFlow website (as of February 2025): ¥2 per million input tokens and ¥8 per million output tokens for DeepSeek V3; ¥4 per million input tokens and ¥16 per million output tokens for DeepSeek R1 [24]. During the study, the applicable exchange rate was US $1=¥7.52. Total input and output tokens for each model were obtained from the API response logs.

For ChatGPT 4o and o1, both models were queried via the public chat UI. We estimated expenditure based on the monthly subscription fee required for o1 access (ChatGPT Plus, US $20 per month, approximately ¥150.59), which includes up to 50 o1 prompts per month according to OpenAI’s policy at the time of the study. ChatGPT 4o is included in the same subscription tier with no separate prompt limit.

### Data Analysis

All data for this study were collected using Microsoft Excel for Mac 16.95, and the accuracy was analyzed using SPSS software (version 30.0; IBM Corp). For the first three sections, that is, basic concepts, relevant expertise and foundational professional knowledge, we performed the McNemar test to examine the performance among the models. For post hoc multiple comparisons of accuracy rates across multiple groups, we used partitions of chi-square method. To control the risk of type I error, statistical significance was set at *P*<.008 according to the Bonferroni correction α'= α/(k*(k-1)/2). For the professional practical skills, one-way ANOVA was used to compare the performance of the 4 LLMs in processing real-world clinical cases, with statistical significance set at *P*<.05.

### Ethical Considerations

As this study was limited to medical state examination questions and publicly available results, no research involving human participants was conducted. Ethics approval was therefore not required.

## Results

### Overview of Different LLMs’ Performance in the Examination

**Figure 2:**
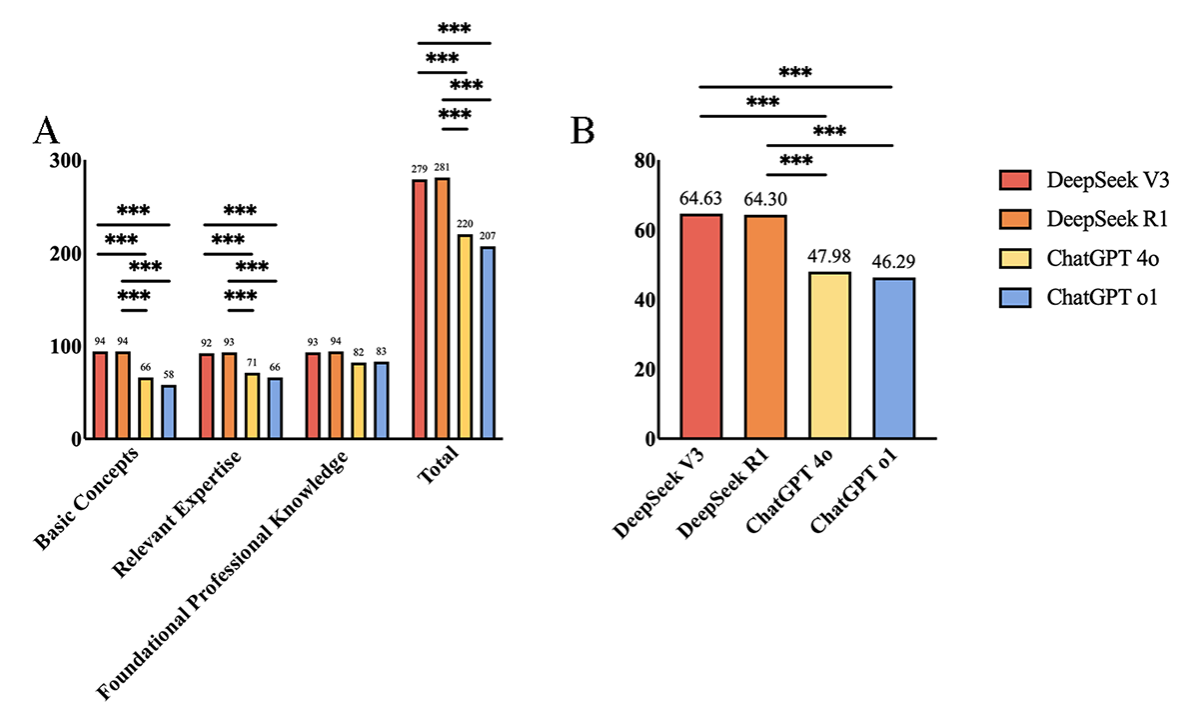
Comparisons among DeepSeek V3, DeepSeek R1, ChatGPT 4o, and ChatGPT o1. (A) Performance on the first three sections. Pairwise model comparisons were performed using McNemar test with Bonferroni correction. (**P<.008; ***P<.001). (B) Performance on the fourth section of the multiple choice questions. Pairwise model comparisons were performed using one-way ANOVA (***P<.001).

As illustrated in the Table 2 and Figure 2A, for the first three parts of the examination, DeepSeek V3, DeepSeek R1, ChatGPT 4o, and ChatGPT o1 showed accuracy of 93%, 93.6%, 73.3%, and 69%, respectively (*χ*^2^_3_=102.9; *P*<.001). Compared with ChatGPT 4o and ChatGPT o1, DeepSeek R1 demonstrated better performance (DeepSeek R1 vs ChatGPT 4o, *χ*^2^_1_=45.1, *P*<.001; DeepSeek R1 vs ChatGPT o1, *χ*^2^_1_= 60.1; *P*<.001) among the 4 LLMs, and DeepSeek V3 ranked second (DeepSeek V3 vs ChatGPT 4o *χ*^2^_1_=41.4; *P*<.001; DeepSeek V3 vs ChatGPT o1 *χ*^2^_1_=56.1; *P*<.001), with no statistically significant differences between R1 and V3 (*χ*^2^_1_=0.1; *P*=.74).

Regarding each individual section, both in basic concepts and relevant expertise sections, ChatGPT 4o and o1 showed lower accuracy of 66% and 58% for the basic concepts part and 71% and 66% for the relevant expertise part, respectively—all with statistical significance compared with 2 models of DeepSeek (*P*<.008). In the section of foundational professional knowledge, the two models of ChatGPT, 4o and o1, showed a moderate increase of accuracy of 82% and 83%, respectively, compared with the first two sections, and showed no statistical significance when the 4 models compared with each other (*χ*^2^_3_=11.3; *P*=.01).

Additionally, for the section on professional practical skills, as illustrated in the Table 2 and Figure 2B, although the two DeepSeek’s models achieved worse performance as they did in the first three sections, DeepSeek V3 and R1 ranked first and second respectively, with scores of 64.63 and 64.3, respectively, while the two models of ChatGPT ranked third and last, with 4o’s score at 47.98 and o1’s at 46.29. This is also of statistical significance compared with DeepSeek V3 (*P*=.003; *P*<.001) and DeepSeek R1 (*P*=.003; *P*<.001), respectively.

**Table 2:** Performance of the different large language models in the examination.

		DeepSeek V3	DeepSeek R1	ChatGPT 4o	ChatGPT o1
**Correct answers in the first 3 sections, n (%)**					
	Basic Concepts (n=100)	94 (94)	94 (94)	66 (66)	58 (58)
	Relevant Expertise (n=100)	92 (92)	93 (93)	71 (71)	66 (66)
	Foundational Professional Knowledge (n=100)	93 (93)	94 (94)	82 (82)	83 (83)
	Total (n=300)	279 (93)	281 (93.6)	220 (73.3)	207 (69)
**Mean scores on the fourth section (n=98, each scored 0-1 point with partial credit)**					
	Professional Practical Skills	64.63	64.3	47.98	46.29

### Performance of LLMs in Various Topics

**Figure 3:**
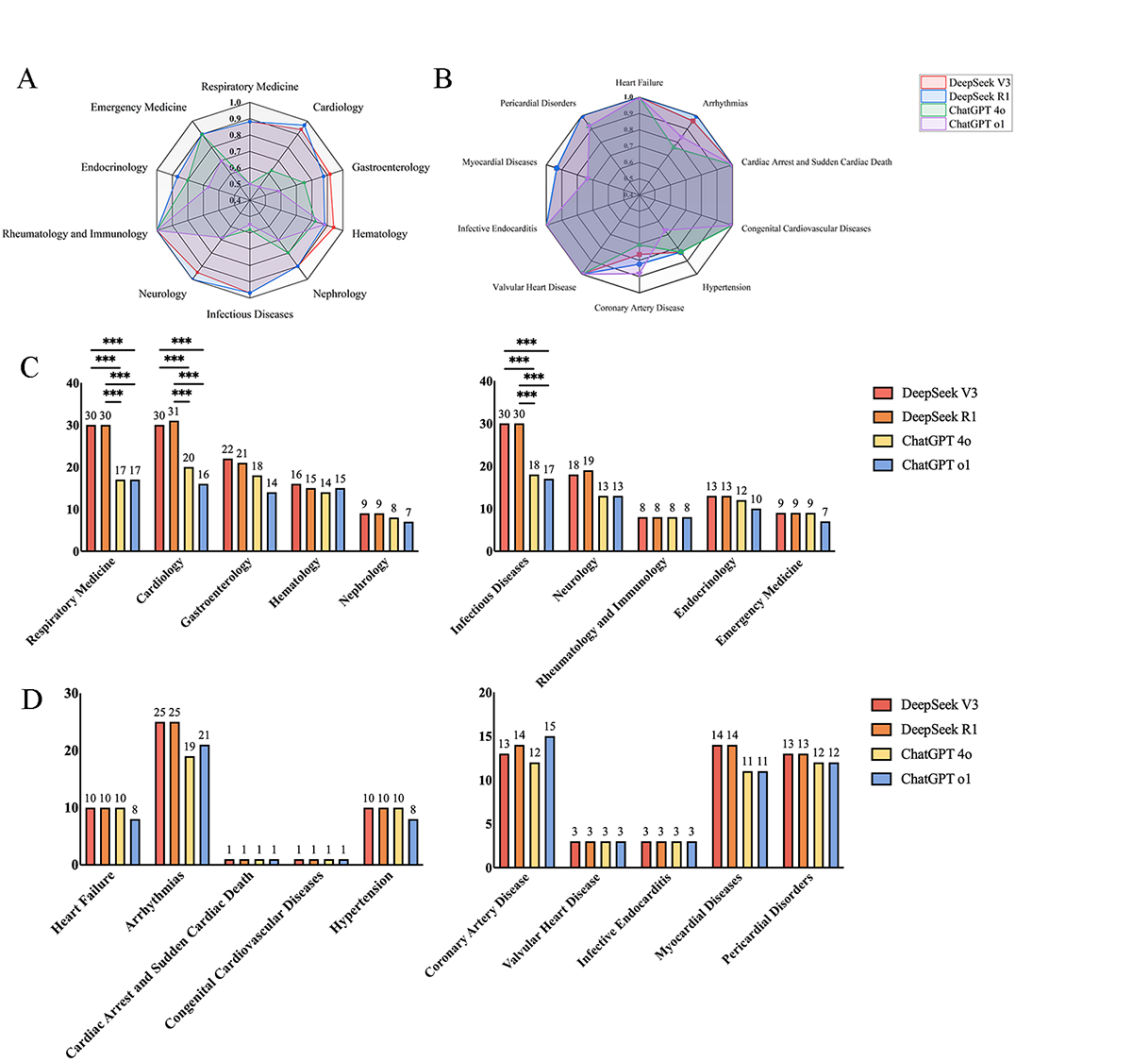
Comparisons among DeepSeek V3, DeepSeek R1, ChatGPT 4o, and ChatGPT o1. (A) and (C) show the performance on the topics of clinical disciplines, and (B) and (D) show the performance on the topics of cardiology subspecialties. Pairwise model comparisons were performed using McNemar test with Bonferroni correction (**P<.008; ***P<.001).

Table 3 presents the percentage of correct answers for each field for each question answered by various LLMs. The topics in Table 3 were manually sorted into 10 clinical disciplines, which contained different branches of internal medicine. Among these topics, DeepSeek-V3 achieved the highest accuracy in two topics, that is, gastroenterology (91.7%) and hematology (94.1%), with no statistical significance compared with DeepSeek R1 (*χ*^2^_1_=1.3; *P*=.26; *χ*^2^_1_=0.4; *P*=.54). DeepSeek R1 achieved the highest accuracy in cardiology (96.9%) and neurology (100%). In the topics of gastroenterology, hematology, nephrology, neurology, rheumatology, immunology, endocrinology, and emergency medicine, the performance of the 4 LLMs did not illustrate statistically significant differences (*χ*^2^_1_=9.4, *P*=.02; *χ*^2^_1_=1.1, *P*=.77; *χ*^2^_1_=3.9, *P*=.27; *χ*^2^_1_=11.4, *P*=.01; *P*>.99; *χ*^2^_1_=2.5, *P*=.48; *χ*^2^_1_=2.3, *P*=.50, respectively). Besides, statistical significance was observed when ChatGPT 4o was compared with DeepSeek V3 and R1 in the fields of respiratory medicine (*χ*^2^_1_=14.8, *P*<.001; *χ*^2^_1_=11.6, *P*<.001), cardiology (*χ*^2^_1_=9.1, *P*=.002; *χ*^2^_1_=11.7, *P*<.001), and infectious diseases (*χ*^2^_1_=14.8, *P*<.001; *χ*^2^_1_=14.8, *P*<.001). As for ChatGPT-o1, statistical significance was observed also in respiratory medicine (*χ*^2^_1_=14.8, *P*<.001; *χ*^2^_1_=11.6, *P*<.001), cardiology (*χ*^2^_1_=15.8, *P*<.001; *χ*^2^_1_=18.8, *P*<.001), and infectious diseases (*χ*^2^_1_=14.9, *P*<.001; *χ*^2^_1_=14.9, *P*<.001) in comparison with DeepSeek V3 and R1 (Figures 3A-C).

Meanwhile, for the third section of foundational professional knowledge, 100 questions were categorized into 10 prevalent cardiology subspecialties. As presented in Table 3 and Figures 3B-D, the performance of ChatGPT 4o and o1, in the field of arrhythmias, hypertension, myocardial diseases and pericardial disorders, was poorer than that of DeepSeek V3 and R1, while it was of no statistical significance (*χ*^2^_1_=9.3, *P*=.03; *χ*^2^_1_=1.5, *P*=.68; *χ*^2^_1_=4.3, *P*=.23; *χ*^2^_1_=2.1, *P*=.56). Particularly, it is noteworthy that in the field of coronary artery disease, the accuracy of ChatGPT o1 was 88.2% and ranked the highest even when no statistical significance was observed compared with DeepSeek V3 and R1 (*χ*^2^_1_=0.8, *P*=.37; *χ*^2^_1_=0.2, *P*=.63).

**Table 3:** Correct answers by clinical discipline in the Basic Concepts and Relevant Expertise section and by cardiology subspecialty in the Foundational Professional Knowledge section.

			DeepSeek V3, n (%)		DeepSeek R1, n (%)		ChatGPT 4o, n (%)		ChatGPT o1, n (%)
**Basic Concepts and Relevant Expertise section (n=200)**									
	Respiratory medicine (n=34)	30 (88.2)		30 (88.2)		17 (50)		17 (50)	
	Cardiology (n=32)	30 (93.6)		31 (96.9)		20 (62.5)		16 (50)	
	Gastroenterology (n=24)	22 (91.7)		21 (87.5)		18 (75)		14 (58.3)	
	Hematology (n=17)	16 (94.1)		15 (88.2)		14 (82.3)		15 (88.2)	
	Nephrology (n=10)	9 (90)		9 (90)		8 (80)		7 (70)	
	Infectious diseases (n=31)	30 (96.8)		30 (96.8)		17 (54.8)		17 (54.8)	
	Neurology (n=19)	18 (94.7)		19 (100)		13 (68.4)		13 (68.4)	
	Rheumatology and immunology (n=8)	8 (100)		8 (100)		8 (100)		8 (100)	
	Endocrinology (n=15)	13 (86.7)		13 (86.7)		12 (80)		10 (66.7)	
	Emergency medicine (n=10)	9 (90)		9 (90)		9 (90)		7 (70)	
**Cardiology subspecialty in the Foundational Professional Knowledge section (n=100)**									
	Heart failure (n=10)	10 (100)		10 (100)		10 (100)		8 (80)	
	Arrhythmias (n=25)	24 (96)		25 (100)		19 (76)		21 (84)	
	Congenital cardiovascular diseases (n=1)	1 (100)		1 (100)		1 (100)		1 (100)	
	Cardiac arrest and sudden cardiac death (n=1)	1 (100)		1 (100)		1 (100)		1 (100)	
	Hypertension (n=12)	10 (83.3)		10 (83.3)		10 (83.3)		8 (66.7)	
	Coronary artery disease (n=17)	13 (76.5)		14 (82.4)		12 (70.6)		15 (88.2)	
	Valvular heart disease (n=3)	3 (100)		3 (100)		3 (100)		3 (100)	
	Infective endocarditis (n=3)	3 (100)		3 (100)		3 (100)		3 (100)	
	Myocardial diseases (n=15)	14 (93.3)		14 (93.3)		11 (73.3)		11 (73.3)	
	Pericardial disorders (n=13)	13 (100)		13 (100)		12 (92.3)		12 (92.3)	

### Performance of LLMs in Different Question Types

**Figure 4:**
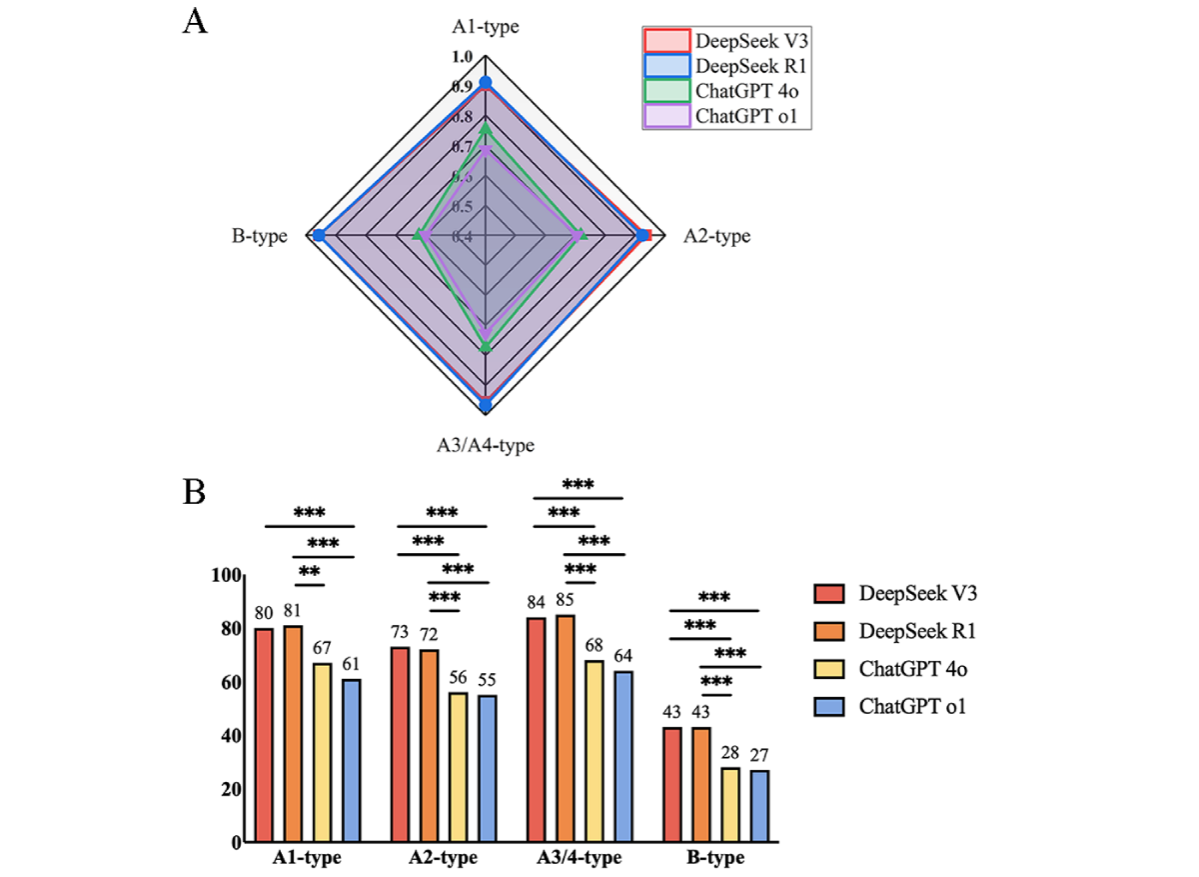
Comparisons among DeepSeek V3, DeepSeek R1, ChatGPT 4o, and ChatGPT o1. (A) and (B) show the performance on the topics of different question types. Pairwise model comparisons were performed using McNemar test with Bonferroni correction (**P<.008; ***P<.001).

As mentioned before, questions in basic concepts, relevant expertise, and foundational professional knowledge were separated into 4 types, namely, A1 (knowledge-based multiple choice), A2 (case-based multiple choice), A3/A4 (case-group-based multiple choice), and B (matching). As illustrated in Table 4 and Figure 4, DeepSeek V3 and R1 showed distinct capability in answering various question types, even though they did not show a comparable difference (*χ*^2^_1_=0.06, *P*=.80; *χ*^2^_1_=0.09, *P*=.75; *χ*^2^_1_=0.2, *P*=.70; *P*>.99). Specifically, DeepSeek R1 ranked the highest in A1-type (91%) and A3/4-type (96.6%), while DeepSeek V3 ranked first in A2 (93.6%). Except for A1 type questions (ChatGPT 4o vs DeepSeek V3 *χ*^2^_1_=6.6; *P*=.01), ChatGPT 4o and o1 demonstrated an equal weakness in other 3 question types, with statistically significant differences compared with DeepSeek V3 and R1 (*P*<.001).

To make it more transparent and demonstrate the performance differences among models, the representative questions and their responses made by the LLMs were chosen and are presented in Table S2 in Multimedia Appendix 1.

**Table 4:** Performance of the 4 large language models by question format in the first 3 sections.

Question format	DeepSeek V3, n (%)	DeepSeek R1, n (%)	ChatGPT 4o, n (%)	ChatGPT o1, n (%)
A1-type (n=89)	80 (89.9)	81 (91)	67 (75.3)	61 (68.5)
A2-type (n=78)	73 (93.6)	72 (92.3)	56 (71.8)	55 (70.5)
A3/4-type (n=88)	84 (95.5)	85 (96.6)	68 (77.3)	64 (72.7)
B-type (n=45)	43 (95.6)	43 (95.6)	28 (62.2)	27 (60)

### Cost-Effectiveness

In total, there were approximately 251,190 input tokens used to prompt DeepSeek V3 and R1. The responses made by DeepSeek V3 contained 22,530 output tokens, while for R1, there were about 48,575 output tokens when compiling all the responses. Based on the token counts recorded during API queries, the total cost for DeepSeek V3 was approximately ¥0.68, and for DeepSeek R1 ¥1.78. For ChatGPT 4o and o1, the estimated cost using the monthly subscription model was ¥37.65 for the 8-day data collection period. Under these specific access conditions, the expense of using ChatGPT models was approximately 55-fold higher than using DeepSeek V3 and 21-fold higher than using DeepSeek R1.

## Discussion

Our study aimed to evaluate and compare the performance of four LLMs—DeepSeek V3, DeepSeek R1, ChatGPT 4o, and ChatGPT o1—in answering medical questions within a Chinese-language context. As demonstrated above, our findings indicated that DeepSeek V3 and R1 showed comparable and superior overall performance compared to both ChatGPT models across multiple question types and clinical disciplines. In the first 3 sections, DeepSeek R1 achieved the best performance, slightly exceeding DeepSeek V3. Additionally, DeepSeek V3 and R1 achieved the highest accuracy in the greatest number of clinical topics. Regarding performance across different question types, DeepSeek R1 performed exceptionally well on A1 and A3/4 types, while DeepSeek V3 performed better on A2 type. Notably, ChatGPT o1, which was thought to be comparable on reasoning tasks to DeepSeek R1 and better than ChatGPT 4o, performed the poorest across most question types among the 4 LLMs. However, all models showed a notable performance decline in realistic clinical case simulations (professional practical skills section).

Our findings demonstrated substantial accordance with previous research. Xu et al [27] reported that in ophthalmology, DeepSeek R1, with overall accuracy of 86.2% on Chinese MCQs, showed superior performance in Chinese complex reasoning tasks compared to Gemini 2.0 Pro, OpenAI o1 and o3-mini. Similarly, Mikhail et al [28] illustrated that DeepSeek R1 had comparable performance with ChatGPT o1 at reduced cost. However, DeepSeek R1 outperformed ChatGPT-4 on pediatric MCQs [19]. Integrating previous research outcomes with our new evidence, LLMs, in particular, DeepSeek V3 and R1, show extensive medical knowledge under the given settings.

However, both in our research and prior studies [19,27,28], LLMs demonstrated exceptional performance primarily in MCQs. Although these questions were designed to assess examinees' mastery of clinical knowledge, most can be effortlessly answered through mere memorization. MCQs failed to mirror the complexity and depth inherent in real-world clinical judgments, which require gathering and evaluating diverse data to reach evidence-based clinical decisions. To assess the usage of LLMs in an autonomous, real-world context, rigorous testing with authentic data and within practical, real-life conditions is essential [29]. Hence, the fourth section of professional practical skills was designed simulate a real-world clinical case. As evidenced in our study, all 4 LLMs showed marked performance decline compared to their results in the preceding three sections, highlighting their evident limitations in confronting with X-type questions (multiple choices) involving real-world clinical case analyses. Tordjman et al [30] reported that for text-based cases without answer choices, DeepSeek R1 (0.36) preformed similarly as ChatGPT o1 (0.32), reflecting the underperformance of the LLMs on open-ended questions. Consequently, while we firmly believe that LLMs have immense potential to revolutionize clinical decision-making in the future, their limitations in more realistic clinical contexts make us skeptical about their suitability at this stage.

Synthesizing the findings from previous research with the outcomes of our study, we raised the following inquiry: what factors enable DeepSeek to surpass ChatGPT in examinations within a Chinese linguistic framework? The DeepSeek team reported in their article [14] that to train a user-friendly model that can produce clear and coherent chains of thought, they designed a pipeline constructed in 4 stages. These 4 stages incorporate the following components: cold start, reasoning-oriented reinforcement learning, rejection sampling, and supervision of fine-tuning and reinforcement learning for all scenarios [14]. These 4 unique stages may be the elements that set DeepSeek R1 apart from the multitude of LLMs in reasoning tasks, elevating it to a distinct level of excellence. Moreover, despite the absence of publicly available details regarding the precise proportion of Chinese and English corpora used in DeepSeek R1’s training process, we found that in its early version DeepSeek V2, it contained 1.12 times more Chinese tokens than English data [31]. It is reasonable to infer that DeepSeek, a Chinese company, likely prioritizes Chinese corpora over English materials in training its LLMs; thus, the superior performance of DeepSeek V3 and R1 in the examination can be partly attributed to this factor.

Beyond the aforementioned discussion, we found several limitations requiring attention. First, in this study, costs of different LLMs were not calculated precisely, and we are yet to establish a comprehensive framework to assess the costs associated with different models. This may contribute to the underestimation of the actual token usage and associated costs. Second, during the interaction process, we provided a prompt requesting each LLM to furnish a detailed analysis for each question. Nonetheless, we were unable to devise a suitable methodology to thoroughly examine these analyses, which warranted further in-depth investigation. Third, as previously reported, LLMs may generate nonsensical or untrue content in relation to certain sources, which is called hallucinations [32]. The occurrence of such inaccuracies in clinical applications may lead to significant economic repercussions and, more gravely, the loss of life [33]. Additionally, we encompassed only one question referring to the image-based questions. However, as illustrated by Sarangi et al [34,35], even the performance of 4 LLMs, that is, Bing, Claude, ChatGPT, and Perplexity, varied in responding to MCQs based on radiology cases, while all failed to perform remarkably well when compared with residents. Meanwhile, even we compiled our own question database to avoid the risk of dataset contamination noted in prior studies (eg, Mikhail et al [28]), we cannot exclude the possibility that this material was included in the training corpora of the evaluated LLMs. Consequently, high accuracy in this study should not be equated with robust clinical decision-making ability. Last, the temperature for DeepSeek V3 and R1 was set to 0.7, while ChatGPT 4o and o1 were evaluated under default configuration. Thus, the comparison between models accessed via API and those evaluated through chat-based interfaces would have introduced systematic bias in output variability and accuracy [12]. Therefore, we have to admit that our findings reflect comparative performance under these specific experimental conditions rather than inherent model superiority.

In the future, we will concentrate on addressing the limitations mentioned above to further evaluate the disparities in the economic efficiency and the dissemination of erroneous information among various LLMs.

On these 398 questions comprising 5 different question types and 10 fields of different clinic disciplines, DeepSeek V3 and R1 demonstrated comparable performance, both surpassing ChatGPT 4o and o1 within the Chinese linguistic environment under the chosen experimental conditions. Consequently, they showed remarkable potential in facilitating clinical decision-making. Nonetheless, continued research is needed to evaluate their economic efficiency and hallucination.

## Appendix

Multimedia Appendix 1 Questions and representative answers given by different large language models.

## Abbreviations

API: application programming interface
LLM: large language model
MCQ: multiple-choice question
UI: user interface

## Data Availability

The datasets generated or analyzed during this study are available from the corresponding author on reasonable request.
