# Supplementary material for: Performance of DeepSeek V3, DeepSeek R1, ChatGPT 4o, and ChatGPT o1 on the National Health Professional and Technical Qualification Examination (Intermediate Level) in China: Comparative Analysis
Source: JMIR Form Res. 2026 Apr 6;10:e90673. doi: 10.2196/90673 (PMC13096768; doi:10.2196/90673)
Supplement: Multimedia Appendix 1 [file formative_v10i1e90673_app1.docx]

**Table S1. Prompt used in the research to generate responses from Large Language Models in Chinese and its translated version.**

| Prompt (translated version) | In this dialogue, you will assume the role of a physician taking the intermediate professional qualification examination in cardiology. I will provide you with a multiple-choice test paper. Please select the correct answers you deem appropriate and provide detailed analysis for each question. |
| --- | --- |

**Table S2. Representative questions and responses made by different LLMs**

**Table S2A. Representative question where all LLMs responded correctly.**

| Questions | Male patient, 70 years old. History: Cough, sputum for 30 years, shortness of breath on activity for 10 years, bilateral lower limb edema in the last year. Pulmonary function shows obstructive ventilation disorder, 50% of predicted value. The main factor for the development of pulmonary hypertension in this patient is: |
| --- | --- |
| Options | A. Reduction of pulmonary capillary bed  B. Increased blood viscosity  C. Increased blood volume  D. Formation of microemboli in pulmonary capillaries  E. Hypoxia and carbon dioxide retention causing pulmonary arteriolar spasm **(Correct Answer)** |
| Responses of DeepSeek V3 | **Answer:** E  **Analysis:** Hypoxia and carbon dioxide retention causing pulmonary arteriolar spasm is the most important factor in the formation of pulmonary hypertension. |
| Responses of DeepSeek R1 | **Answer:** E  **Analysis:** Hypoxia leads to pulmonary vasoconstriction (hypoxic pulmonary vasoconstriction), which progresses over the long term into pulmonary hypertension. This is the primary mechanism for the formation of pulmonary hypertension in COPD. |
| Responses of ChatGPT 4o | **Answer:** E  **Analysis:** Pulmonary hypertension caused by emphysema is primarily due to hypoxia and carbon dioxide retention leading to spasm and vasoconstriction of the small pulmonary arteries. |
| Responses of ChatGPT o1 | **Answer:** E  **Analysis:** Patients with emphysema often experience impaired oxygenation, leading to hypoxia and carbon dioxide retention. This, in turn, causes spasm of the small pulmonary arteries, resulting in pulmonary hypertension. |

**Table S2B. Representative question where only DeepSeek V3 responded correctly.**

| Questions | Male patient, 29 years old. Elevated BP for over 2 years, accompanied by paroxysmal headache, palpitations. Local hospital gave amlodipine, irbesartan, BP control fair. Our hospital: Norepinephrine 4911.40pg/ml (ref 19-121), Metanephrine 23.94pg/ml (ref 14-90). Whole abdomen enhanced CT: Hypervascular tumors at right renal hilum and left para-aortic area. For tumor resection, the preferred preoperative antihypertensive is: |
| --- | --- |
| Options | A. Bisoprolol B. Perindopril C. Indapamide D. Clonidine E. Terazosin **(Correct Answer)** |
| Responses of DeepSeek V3 | **Answer:** E  **Analysis:** The patient has classic manifestations of pheochromocytoma. The preferred preoperative antihypertensive medication is terazosin (an alpha-blocker). |
| Responses of DeepSeek R1 | **Answer:** A  **Analysis:** The patient was diagnosed with pheochromocytoma. Preoperative management requires alpha-blockers (such as phenoxybenzamine) for blood pressure control, but this was not among the available options. Therefore, a beta-blocker (bisoprolol) was selected to assist with heart rate control. It is critical to note that beta-blockers must only be administered after adequate alpha-blockade has been established to prevent a hypertensive crisis. |
| Responses of ChatGPT 4o | **Answer:** D  **Analysis:** For a patient with suspected pheochromocytoma, clonidine is not an appropriate antihypertensive agent for preoperative use. |
| Responses of ChatGPT o1 | **Answer:** D  **Analysis:** This patient presents with paroxysmal hypertension and elevated adrenergic levels, suggesting a possible pheochromocytoma. Preoperative management requires the use of an alpha-antagonist (such as clonidine) for blood pressure control. |

**Table S2C. Representative question where only DeepSeek R1 responded correctly.**

| Questions | The most common early arterial blood gas analysis finding in an acute asthma attack is: |
| --- | --- |
| Options | A. Decreased PaO₂, increased PaCO₂, increased pH B. Decreased PaO₂, increased PaCO₂, decreased pH C. Normal PaO₂, increased PaCO₂, decreased pH D. Decreased PaO₂, normal PaCO₂, decreased pH E. Decreased PaO₂, decreased PaCO₂, increased pH **(Correct Answer)** |
| Responses of DeepSeek V3 | **Answer:** B  **Analysis:** The most common arterial blood gas finding in the early stage of an acute asthma attack is decreased PaO₂, elevated PaCO₂, and decreased pH. |
| Responses of DeepSeek R1 | **Answer:** E  **Analysis:** In the early stage of asthma, hyperventilation leads to respiratory alkalosis (↓PaCO₂, ↑pH). During a severe attack, PaCO₂ may be normal or elevated (indicating respiratory muscle fatigue). |
| Responses of ChatGPT 4o | **Answer:** D  **Analysis:** In the early stage of an acute asthma attack, PaO₂ (partial pressure of oxygen) typically decreases, PaCO₂ (partial pressure of carbon dioxide) may remain normal or be slightly low, and the pH decreases, indicating respiratory acidosis. |
| Responses of ChatGPT o1 | **Answer:** D  **Analysis:** During an acute asthma attack, impaired oxygenation typically leads to a decreased PaO₂. When carbon dioxide elimination becomes compromised, PaCO₂ normalizes, and the pH falls, manifesting as acidosis. |

**Table S2D. Representative question where only ChatGPT 4o responded correctly.**

| Questions | Male patient, 35 years old. Thirst, polydipsia, polyuria for 1 month. Fasting blood glucose 18mmol/L, urine sugar (+++), ketones (±); his mother has diabetes for 5 years. Physical examination: Height 167cm, Weight 75kg, heart, lungs, abdomen (-). Based on this patient's condition, the best treatment plan is: |
| --- | --- |
| Options | A. Short-acting insulin 3 times + intermediate-acting insulin B. Intermediate/Long-acting insulin + sulfonylurea oral hypoglycemic C. Sulfonylurea oral hypoglycemic + glucosidase inhibitor D. Sulfonylurea oral hypoglycemic + biguanide oral hypoglycemic  **(Correct Answer)** E. Short/Intermediate mixed insulin + biguanide oral hypoglycemic |
| Responses of DeepSeek V3 | **Answer:** E  **Analysis:** The patient's condition is severe. A combination of short- and intermediate-acting mixed insulin with a biguanide oral hypoglycemic agent is the optimal treatment regimen. |
| Responses of DeepSeek R1 | **Answer:** E  **Analysis:** The patient has a normal body weight and a high likelihood of type 1 diabetes. Insulin therapy (short-acting + intermediate-acting) is recommended, combined with a biguanide to improve insulin resistance. |
| Responses of ChatGPT 4o | **Answer:** D  **Analysis:** For patients with type 2 diabetes, sulfonylureas and biguanides are commonly used treatment regimens. They work by increasing insulin secretion and improving insulin resistance to control blood glucose. |
| Responses of ChatGPT o1 | **Answer:** B  **Analysis:** For this type of diabetic patient, a combination of intermediate- or long-acting insulin and a sulfonylurea oral hypoglycemic agent is a common treatment strategy." |

**Table S2E. Representative question where no LLMs responded correctly.**

| Questions | Male patient, 32 years old. Past history of hypertension for many years, self-reported irregular antihypertensive use, poor BP control. Tearing chest pain 1 hour ago without obvious cause, severe unbearable pain, our emergency BP 185/100mmHg, completed thoracic-abdominal aortic CTA: Aortic dissection, DeBakey type I. The BP management goal for this patient is: |
| --- | --- |
| Options | A. Reduce SBP to 120mmHg within 30 minutes B. Reduce SBP to 100mmHg within 30 minutes **(Correct Answer)** C. Reduce SBP to 120mmHg within 2 hours D. Reduce SBP to 100mmHg within 2 hours E. To maintain peripheral circulation stability, cannot rapidly lower BP, BP should gradually normalize over 4-8 hours. |
| Responses of DeepSeek V3 | **Answer: D**  **Analysis:** The blood pressure management goal for a patient with aortic dissection is to reduce the systolic blood pressure to 100 mmHg within 2 hours. |
| Responses of DeepSeek R1 | **Answer: C**  **Analysis:** For an aortic dissection (DeBakey type I), rapid reduction of systolic blood pressure to 100-120 mmHg and control of heart rate to <60 bpm is required. However, among the options provided, the target described is a systolic blood pressure of 120 mmHg. Therefore, the option that most closely aligns with guideline recommendations should be selected. |
| Responses of ChatGPT 4o | **Answer:** **E**  **Analysis:** The treatment goal for aortic dissection is to gradually control blood pressure, rather than rapid reduction, because rapid blood pressure reduction may increase the risk of dissection rupture. |
| Responses of ChatGPT o1 | **Answer: E**  **Analysis:** Blood pressure management in aortic dissection should avoid rapid reduction to prevent further damage, and gradual control is recommended. |

**Table S2F. Representative questions of the section of Professional Practical Skills.**

| Questions | **[Case 8]** Male patient, 43 years old. Admitted due to sudden chest/back pain during activity for 2 hours, syncope once. Hypertension and diabetes history 8 years, no chronic cough or diabetes history. Physical examination: Acute ill appearance, BP 195/105mmHg, R 28 bpm, HR 45 bpm. Irregular rhythm, P2 accentuated, diastolic rumbling murmur audible at left sternal border 3rd intercostal space. ECG: Complete right bundle branch block, ST segment horizontal depression 0.2mV in II, III, aVF. |
| --- | --- |
| Options | **Question 1:** **The preliminary diagnosis may be:**  A. Acute massive pulmonary embolism **(Correct Answer)**  B. Rheumatic heart disease, aortic insufficiency with acute left heart failure C. Severe myocarditis D. Acute inferior wall non-ST elevation myocardial infarction **(Correct Answer)**  E. Aortic dissection involving right coronary ostium **(Correct Answer)**  F. Tension pneumothorax  **Question 2：If chest X-ray suggests widened mediastinum, and echocardiography suggests significantly enhanced aortic intima echo with displacement into the aortic lumen, the core content for further emergency examination is:**  A. Pulmonary artery CTA B. Pulmonary vein CTA C. Coronary CTA D. Aortic CTA **(Correct Answer)** E. Cardiac MRI F. Cardiac ECT  **Question 3: Besides hypertension, the causes of this type of disease also include:** A. Cystic medial necrosis of aorta  **(Correct Answer)** B. Congenital cardiovascular disease  **(Correct Answer)** C. Atherosclerosis **(Correct Answer)** D. Takayasu's arteritis  **(Correct Answer)** E. Cardiac interventional procedures  **(Correct Answer)** F. Trauma **(Correct Answer)**  **Question 4:** **The general treatment principles for this type of disease include:**  A. Absolute bed rest **(Correct Answer)** B. Analgesia and sedation **(Correct Answer)** C. Blood pressure control **(Correct Answer)** D. Heart rate reduction **(Correct Answer)** E. Heart failure control **(Correct Answer)** F. Surgical evaluation for indication and timing  **(Correct Answer)**  **Question 5:** **Incorrect internal medicine drug use in the acute phase for this patient is:**  A. Morphine B. Low molecular weight heparin **(Correct Answer)** C. Sodium nitroprusside D. Metoprolol **(Correct Answer)** E. Aspirin + Clopidogrel **(Correct Answer)** F. Ticagrelor **(Correct Answer)** |
| Responses of DeepSeek V3 | **Question 1:**  **Answer:** D, E  **Analysis:**  Acute Inferior Non-ST-Elevation Myocardial Infarction: The patient presents with chest and back pain and syncope. The electrocardiogram shows horizontal ST-segment depression in leads II, III, and aVF, which is consistent with inferior wall myocardial ischemia.  Aortic Dissection Involving the Right Coronary Ostium: Chest and back pain, syncope, a loud P₂ sound, and a diastolic rumbling murmur suggest aortic dissection. Involvement of the right coronary artery ostium can lead to inferior wall myocardial ischemia.  Ruled-out Alternatives:  Acute Massive Pulmonary Embolism: No significant dyspnea or hypoxemia is present.  Rheumatic Heart Disease with Aortic Insufficiency and Acute Left Heart Failure: There is no history of valvular disease, and no signs of left heart failure are evident.  Severe Myocarditis: There is no history of infection, and no evidence of cardiac insufficiency exists.  Tension Pneumothorax: Typical manifestations such as dyspnea and tracheal deviation are absent.  **Question 2:**  **Answer:** D  **Analysis:**  Aortic CTA is the gold standard for diagnosing aortic dissection. It can clearly define the extent of the dissection, the involved vessels, and any complications.  Other Options:  Pulmonary Artery CTA: Applicable for the diagnosis of pulmonary embolism.  Pulmonary Vein CTA, Coronary CTA, Cardiac MRI, Cardiac ECT: These are not appropriate for the diagnosis of aortic dissection.  **Question 3:**  **Answer:** A, B, C, D, E, F  **Analysis:**  The primary etiologies of aortic dissection include:  Cystic medial degeneration (e.g., Marfan syndrome)  Congenital cardiovascular disease (e.g., coarctation of the aorta)  Atherosclerosis  Aortitis (e.g., Takayasu's arteritis)  Cardiac interventional procedures or aortic surgery (iatrogenic causes)  Trauma (e.g., blunt chest trauma)  **Question 4:**  **Answer:** A, B, C, D, F  **Analysis:**  Absolute Bed Rest: To avoid exacerbating the condition through physical activity.  Analgesia and Sedation: To relieve pain and mitigate sympathetic nervous system excitation.  Blood Pressure Control: To reduce pressure on the aortic wall and prevent extension of the dissection.  Heart Rate Reduction: To diminish shear stress on the aortic wall.  Surgical Evaluation for Indication and Timing: The decision for and timing of surgery is based on the type of dissection and the extent of involvement.  Heart Failure Management: Aortic dissection does not typically cause heart failure directly, unless it involves the aortic valve or coronary arteries.  **Question 5:**  **Answer:** E, F  **Analysis:**  Aspirin + Clopidogrel / Ticagrelor: These are antiplatelet agents indicated for Acute Coronary Syndrome. However, their use in patients with aortic dissection may increase the risk of bleeding and is contraindicated, especially prior to potential surgical intervention.  Other Options:  Morphine: Used for analgesia.  Low Molecular Weight Heparin: Used for anticoagulation but must be used with extreme caution in aortic dissection.  Sodium Nitroprusside: For rapid blood pressure reduction.  Metoprolol: To lower heart rate and reduce shear stress on the aortic wall. |
| Responses of DeepSeek R1 | **Question 1:**  **Answer:** E  **Analysis:**  The patient presents with sudden-onset chest and back pain, syncope, and a history of hypertension. Physical examination reveals a loud P₂ and a diastolic murmur at the left sternal border (suggesting aortic root dilation or dissection). The ECG shows ST-segment depression in the inferior leads (possibly indicating myocardial ischemia due to dissection involving the right coronary artery).  Aortic dissection involving the right coronary ostium (Option E) is a classic presentation. Other options (such as pulmonary embolism or acute myocardial infarction) can be ruled out through further investigation.  **Question 2:**  **Answer:** D  **Analysis:**  Aortic CTA is the 'gold standard' for diagnosing aortic dissection. It can clearly define the extent of the dissection (Stanford classification) and whether the coronary arteries are involved. Other imaging modalities (such as cardiac MRI or coronary CTA) should be selected based on the clinical context and are not the first-line choice for emergency diagnosis.  **Question 3:**  **Answer:** A, C, F  **Analysis:**  Cystic medial degeneration (A): Conditions like Marfan syndrome led to structural abnormalities of the aortic wall.  Atherosclerosis (C): Plaque rupture can induce dissection.  Trauma (F): Chest trauma can directly cause aortic dissection.  Other etiologies include congenital coarctation of the aorta (B) or Takayasu's arteritis (D).  **Question 4:**  **Answer:** A、B、C、D、F  **Analysis:**  Blood Pressure Control (C): Requires reduction of systolic blood pressure to 100-120 mmHg (commonly using sodium nitroprusside + a beta-blocker).  Heart Rate Control (D): Target heart rate <60 bpm to reduce aortic shear stress (e.g., intravenous esmolol).  Surgical Evaluation (F): Stanford type A dissection requires emergency surgery.  **Question 5:**  **Answer:** E、F  **Analysis:**  Antiplatelet agents (E, F) may increase the risk of dissection rupture or bleeding (and are contraindicated unless complicated by acute myocardial infarction or requiring interventional therapy).  Sodium nitroprusside (C), beta-blockers (D), and morphine (A) are all components of standard medical therapy. |
| Responses of ChatGPT 4o | **Question 1:**  **Answer:** E  **Analysis:**  The patient presents with sudden-onset chest and back pain, syncope, right bundle branch block on ECG, and is accompanied by a diastolic rumbling murmur (suggesting aortic valve pathology) and a history of hypertension. These findings collectively point towards aortic dissection, particularly a dissection involving the right coronary artery ostium. Aortic dissection can cause acute chest pain and syncope. If it involves the right coronary artery, it may also lead to myocardial ischemia or arrhythmias.  While other conditions such as pulmonary embolism, rheumatic heart disease, myocarditis, or myocardial infarction can also cause chest pain, the patient's specific combination of symptoms and signs makes aortic dissection the most likely diagnosis.  **Question 2:**  **Answer:** D  **Analysis:** The chest X-ray shows a widened mediastinum, and the echocardiogram suggests a significantly enhanced aortic intimal echo with displacement into the aortic lumen, indicating a possible aortic dissection.  To confirm the diagnosis and evaluate the extent and impact of the dissection, Aortic CTA (Computed Tomographic Angiography) is the preferred imaging modality for diagnosing aortic dissection. It can clearly display the aortic anatomy and the location of the dissection.  Other examinations, such as Pulmonary Artery CTA, Coronary CTA, or Cardiac MRI, are not suitable for the primary diagnosis of aortic dissection.  **Question 3:**  **Answer:** A, D, F  **Analysis:**  Cystic Medial Degeneration: This is a congenital/hereditary defect of the aortic wall (common in genetic disorders like Marfan syndrome) that predisposes individuals to aortic dissection. The risk is significantly heightened when combined with hypertension.  Aortitis: Inflammatory conditions such as Takayasu's arteritis can cause inflammation of the aortic wall, increasing its vulnerability and the risk of dissection.  Trauma: Severe physical trauma can also lead to aortic rupture or the initiation of a dissection.  Other Factors: Conditions including congenital cardiovascular disease (e.g., aortic coarctation), atherosclerosis, and cardiac interventional procedures are also established etiologies that can directly lead to aortic dissection.  **Question 4:**  **Answer:** A, B, C, D, F  **Analysis:**  Absolute Bed Rest: Patients with aortic dissection must maintain complete stillness to avoid any activity that could increase cardiac workload and blood pressure.  Analgesia and Sedation: Acute pain exacerbates the patient's condition by driving sympathetic activation and hypertension; analgesia and sedation are crucial for mitigating this response.  Blood Pressure Control: Controlling hypertension is paramount to preventing the progression of the dissection. This is typically achieved using antihypertensive agents (e.g., beta-blockers, calcium channel blockers).  Heart Rate Control: Reducing the heart rate decreases the frequency of pulsatile force on the aortic wall, thereby lowering shear stress.  Surgical Evaluation for Indications and Timing: Surgical intervention is required if the dissection involves the aortic root (Stanford Type A) or leads to major branch vessel compromise, organ ischemia, or impending rupture.  **Question 5:**  **Answer:** C  **Analysis:**  Sodium Nitroprusside is a potent vasodilator that can rapidly lower blood pressure. However, in the acute management of aortic dissection, its use carries a risk of reflex tachycardia due to a sudden drop in blood pressure. For this reason, guidelines mandate that nitroprusside must always be used in combination with a beta-blocker in this setting and is not recommended as a first-line single-agent therapy.  Other medications such as morphine (for pain relief), low molecular weight heparin (for thrombosis prevention), and metoprolol (to lower heart rate and blood pressure) are components of standard management. In contrast, antiplatelet agents like aspirin + clopidogrel or ticagrelor are typically contraindicated in acute aortic dissection due to the increased risk of bleeding, particularly if surgery is anticipated. |
| Responses of ChatGPT o1 | **Question 1:**  **Answer:** E  **Analysis:**  E. Aortic Dissection Involving the Right Coronary Ostium: The patient presents with severe chest and back pain, syncope, horizontal ST-segment depression, and a history of hypertension, which are clinically consistent with aortic dissection. The diastolic rumbling murmur also suggests possible aortic valve insufficiency, particularly if the aortic dissection affects the right coronary ostium.  Other Options:  A. Acute Massive Pulmonary Embolism: Pulmonary embolism is typically accompanied by acute dyspnea, chest pain, etc., but this patient did not exhibit typical dyspnea symptoms.  B. Rheumatic Heart Disease with Aortic Insufficiency and Acute Left Heart Failure: The patient has no history of rheumatic heart disease, and the symptoms are inconsistent with this diagnosis.  C. Severe Myocarditis: Severe myocarditis is usually accompanied by more pronounced heart failure and electrophysiological abnormalities, which do not match the patient's presentation.  D. Acute Inferior Non-ST-Elevation Myocardial Infarction: The patient shows horizontal ST-segment depression, which does not meet the criteria for ST-segment elevation myocardial infarction.  **Question 2:**  **Answer:** D  **Analysis:**  D. Aortic CTA: The chest X-ray suggests a widened mediastinum, and the echocardiogram indicates a significantly enhanced aortic intimal echo. These findings point to a possible aortic dissection. Aortic CTA is the most effective imaging modality for confirming the diagnosis of aortic dissection.  Other Options:  A. Pulmonary Artery CTA: Primarily used for diagnosing pulmonary embolism and is not relevant for diagnosing aortic dissection.  B. Pulmonary Vein CTA: Not applicable for the diagnosis of aortic dissection.  C. Coronary CTA: Mainly used for diagnosing coronary artery disease and is not related to aortic dissection.  E. Cardiac MRI: While it can also be used to diagnose aortic dissection, Aortic CTA holds distinct advantages.  F. Cardiac ECT: Primarily used for diagnosing myocardial ischemia.  **Question 3:**  **Answer:** A, D, F  **Analysis:**  A. Cystic Medial Degeneration: Cystic medial degeneration (as seen in conditions like Marfan syndrome) is one of the common causes of aortic dissection.  D. Aortitis: Inflammatory conditions such as Takayasu's arteritis can also lead to aortic dissection.  F. Trauma: Trauma can cause aortic tearing or dissection, particularly blunt chest trauma.  Other Options:  B. Congenital Cardiovascular Disease: While certain congenital conditions like aortic coarctation are associated with dissection, the broad category of "Congenital Cardiovascular Disease" is not a precise fit in this specific etiological context, especially given the misclassification of Marfan syndrome here.  C. Atherosclerosis: Although atherosclerosis increases cardiovascular risk, the statement that it is "not a direct cause" is inaccurate. Atherosclerotic plaque ulceration is a recognized, direct mechanism for initiating aortic dissection.  **Question 4:**  **Answer:** A, B, C, D, F  **Analysis:**  A. Absolute Bed Rest: Patients with aortic dissection should remain quiet and avoid strenuous activity to prevent exacerbation of the condition.  B. Analgesia and Sedation: Analgesia and sedation help alleviate the patient's suffering and reduce blood pressure fluctuations caused by severe pain.  C. Blood Pressure Control: Controlling blood pressure reduces the risk of aortic dissection rupture; beta-blockers are typically used for this purpose.  D. Heart Rate Control: Reducing the heart rate lightens the load on the aorta, helping to minimize the extension of the dissection.  F. Surgical Evaluation for Indications and Timing: Patients with severe aortic dissection require surgical intervention, making timely assessment of the operative timing critically important.  **Question 5:**  **Answer:** C  **Analysis:**  C. Sodium Nitroprusside: Although sodium nitroprusside can lower blood pressure, it is a potent vasodilator. Its use carries a risk of causing reflex tachycardia, which can increase shear stress on the aortic wall and potentially worsen the dissection in the acute phase. Therefore, it should not be used as a first-line single agent and requires extreme caution with concomitant beta-blockade if utilized.  A. Morphine: Morphine has analgesic effects, which can relieve pain and reduce sympathetic nervous system activation, benefiting patients with aortic dissection.  B. Low Molecular Weight Heparin (LMWH): LMWH can be used to prevent thrombus formation, particularly when there is a need to prevent embolism in the false lumen during the acute phase. However, its use requires careful risk-benefit assessment.  D. Metoprolol: Metoprolol effectively lowers both heart rate and blood pressure and is a first-line medication in the acute phase of aortic dissection.  E. Aspirin + Clopidogrel / F. Ticagrelor: These antiplatelet agents are typically used in acute coronary syndromes. However, in patients with aortic dissection, they are generally contraindicated due to the significantly increased risk of bleeding, especially if surgical intervention is anticipated. |

***The answers and analyses in the Table S2 have been translated into English for better comprehension.**

**Basic Concepts**

1. The coagulation abnormality in hemophilia is:

A. Shortened APTT, normal PT
B. Normal APTT, prolonged PT
C. Prolonged APTT, normal PT
D. Normal APTT, normal PT
E. Prolonged APTT, prolonged PT

1. The standard for massive upper gastrointestinal bleeding is:

A. Single blood loss exceeding 5% of total blood volume
B. Single blood loss exceeding 10% of total blood volume
C. Single blood loss exceeding 15% of total blood volume
D. Single blood loss exceeding 20% of total blood volume
E. Multiple blood losses exceeding 10% of total blood volume

1. Under normal circumstances, the site where lymph nodes can be palpated is:

A. Submandibular
B. Neck
C. Supraclavicular fossa
D. Axillary fossa
E. Postauricular lymph nodes

1. The lower edge of the liver is generally not palpable below the costal margin; the lower edge of the liver palpable below the xiphoid process is mostly:

A. 3cm
B. 4cm
C. 5cm
D. 1cm
E. 2cm

1. Which of the following does not belong to the nature of chest pain?

A. Burning pain
B. Knife-like pain
C. Referred pain
D. Colicky pain
E. Stabbing pain

1. The site where pleural friction rub is most easily heard on auscultation is:

A. Bilateral lung apices
B. Bilateral lung bases
C. Bilateral lower scapular regions
D. Bilateral upper breast areas
E. Anterolateral chest wall

1. Regarding the description of spleen size and position, the correct one is:

A. The normal dullness area of the spleen is located between the 8th and 11th intercostal spaces on the left midaxillary line.
B. Palpating the lower edge of the spleen below the left costal arch indicates splenomegaly.
C. Spleen palpation requires bimanual palpation, left lateral decubitus position, and exclusion of ipsilateral kidney position and developmental abnormalities.
D. The lower edge of the spleen exceeding 2cm below the costal margin but above the umbilical level suggests severe splenomegaly.
E. Normally, the anterior part of the spleen does not exceed the anterior axillary line.

1. The cause of massive hemoptysis in mitral stenosis is:

A. Pulmonary congestion
B. Bronchial mucosal microvascular rupture
C. Bronchial venous varices rupture
D. Pulmonary embolism
E. Bronchial artery rupture

1. The common site of aspiration lung abscess is:

A. Lateral basal segment of the left lower lobe
B. Right middle lobe
C. Anterior basal segment of the right lower lobe
D. Left lingular lobe
E. Posterior segment of the right upper lobe

1. The disease that can present with peripheral cyanosis is:

A. Chronic obstructive pulmonary emphysema
B. Pulmonary edema
C. Massive pleural effusion
D. Right heart failure
E. Tetralogy of Fallot

1. The most characteristic sign of pyramidal tract damage is:

A. Quadriplegia
B. Increased muscle tone
C. Hyperactive tendon reflexes
D. Absent abdominal reflex
E. Positive Babinski sign

1. Glove and stocking sensory disturbance suggests the lesion is at:

A. Peripheral nerve endings
B. Single nerve root
C. Spinal cord
D. Brainstem
E. Cerebral hemisphere

1. Signs related to pulmonary emphysema do not include:

A. Decreased vocal resonance
B. Descended liver dullness border
C. Barrel chest
D. Dullness on percussion
E. Decreased respiratory movement

1. Regarding the description of SLE joint involvement, the incorrect one is:

A. Articular cartilage destruction, joint deformity
B. Often involves proximal interphalangeal joints
C. Joint swelling and pain
D. Presents as symmetric polyarticular damage
E. Large joints are rarely involved

1. The principles of glucocorticoid therapy for nephrotic syndrome do not include:

A. Initial dose should be sufficient
B. Full dose throughout the course, combined with immunosuppressants
C. Slow tapering of medication
D. Course of treatment should be long enough
E. Low-dose maintenance therapy

1. The lesion site of locked-in syndrome is located at:

A. Medulla oblongata
B. Pons
C. Midbrain
D. Cerebellum
E. Diencephalon

1. The circle of Willis plays a very important role in cerebral circulation, communicating blood supply between anterior, posterior, left, and right parts of the brain. Which artery does *not* participate in the formation of the circle of Willis?

A. Anterior communicating artery
B. Vertebral artery
C. Internal carotid artery
D. Anterior cerebral artery
E. Posterior cerebral artery

1. The most characteristic pathological change of epidemic encephalitis B is:

A. Neuronophagia
B. Satellitosis
C. Reticular foci of encephalomalacia
D. Lymphocytic cuffing
E. Glial nodules

1. Regarding the description of chronic pancreatitis, the incorrect one is:

A. Hyperlipidemia is related to the pathogenesis of chronic pancreatitis.
B. Long-term alcoholism is the main cause of chronic pancreatitis in Western countries.
C. Biliary tract diseases are the main cause of chronic pancreatitis in China.
D. More common in middle-aged and elderly people.
E. Biliary chronic pancreatitis lesions are mainly located in the body and tail of the pancreas.

1. The most common adverse reaction of cyclophosphamide is:

A. Bone marrow suppression
B. Gastrointestinal adverse reactions
C. Abnormal liver function
D. Gonadal suppression
E. Nervous system damage

1. Regarding the description of the pathogenesis characteristics of primary pulmonary tuberculosis, the incorrect one is:

A. Predilection for lower parts of upper lobes and lower lobes.
B. More common in children.
C. Most can heal spontaneously.
D. The body relies on specific immunity to clear Mycobacterium tuberculosis.
E. Necrotic lesions invading blood vessels evolve into hematogenous disseminated tuberculosis.

1. The most common route of metastasis for liver cancer is:

A. Implantation metastasis
B. Extrahepatic hematogenous metastasis
C. Lymphatic metastasis
D. Direct invasion
E. Intrahepatic hematogenous metastasis

1. The main pathogenic bacteria of acute infective endocarditis is:

A. Pneumococcus
B. Group A Streptococcus
C. Gonococcus
D. Staphylococcus aureus
E. Haemophilus influenzae

1. Related to both the colonization and tissue damage of Helicobacter pylori is:

A. Flagella
B. Urease
C. Adhesin
D. Vacuolating cytotoxin
E. Phospholipase A

1. Regarding the effects of acidosis on the body, the correct one is:

A. Normal oxygen dissociation curve, no shift.
B. Causes hypokalemia.
C. Constricts cerebral vessels.
D. Enhances myocardial contractility.
E. Reduces tissue oxygen utilization.

1. The following is *not* part of the pathogenesis of COPD:

A. Acid-base imbalance mechanism
B. Autonomic nervous dysfunction mechanism
C. Inflammatory mechanism
D. Protease-antiprotease imbalance mechanism
E. Oxidative stress mechanism

1. The effective alveolar ventilation volume in normal adults at rest is approximately:

A. 3.5 L/min
B. 4 L/min
C. 2 L/min
D. 2.5 L/min
E. 3 L/min

1. For respiratory muscle paralysis caused by organophosphorus pesticide poisoning, one should use:

A. Neostigmine
B. Atropine
C. Nikethamide
D. Sodium bicarbonate
E. Pralidoxime iodide

1. Regarding the description of the pathogenesis of carbon monoxide poisoning, the incorrect one is:

A. The affinity of carbon monoxide for hemoglobin is greater than that of oxygen.
B. After forming carboxyhemoglobin, its dissociation rate is faster than that of oxyhemoglobin.
C. The presence of carboxyhemoglobin affects the dissociation of oxyhemoglobin.
D. Affects oxygen diffusion from capillaries to intracellular mitochondria.
E. Blocks the electron transport chain, inhibiting cellular respiration.

1. The incorrect match between leukemia and its typical chromosomal change is:

A. M3 --- t (15;17)
B. M2 --- t (8;21)
C. CMML --- t (9;21)
D. M4 --- 16 chromosome deletion
E. L3 --- t (8;14)

1. If clinical jaundice is detectable, the total bilirubin concentration exceeds:

A. 1.7 µmol/L
B. 3.4 µmol/L
C. 13.68 µmol/L
D. 17.1 µmol/L
E. 34.2 µmol/L

1. The Austin-Flint murmur is:

A. Organic aortic stenosis murmur
B. Relative aortic insufficiency murmur
C. Relative mitral insufficiency murmur
D. Relative mitral stenosis murmur
E. Organic mitral stenosis murmur

1. Displacement of the apical impulse to the left and inferiorly is seen in:

A. Normal person in left lateral decubitus position
B. Left ventricular enlargement
C. Left atrial enlargement
D. Right atrial enlargement
E. Right ventricular enlargement

1. The most common cause of iron deficiency anemia in adult males is:

A. Chronic gastrointestinal blood loss
B. Increased iron requirement
C. Major traumatic bleeding
D. Insufficient iron intake
E. Decreased bone marrow hematopoietic function

1. The normal position of the apical impulse is at:

A. 5th intercostal space, 2.0-2.5cm medial to the left midclavicular line
B. 6th intercostal space, 1.0-2.0cm medial to the left midclavicular line
C. 5th intercostal space, 0.5-1.0cm medial to the left midclavicular line
D. 6th intercostal space, 0.5-1.0cm medial to the left midclavicular line
E. 4th intercostal space, 0.5-1.0cm medial to the left midclavicular line

1. Male patient, 56 years old. Tuberculous pleurisy. Right massive pleural effusion. Upon physical examination, the apical impulse of this patient might:

A. Shift to the right
B. Shift to the left
C. Shift upward
D. Shift downward
E. No change

1. Male patient, 23 years old. Cough recently, expectorating yellow-green sputum. The possible diagnosis for this patient is:

A. Mycoplasma pneumonia
B. Pseudomonas aeruginosa pneumonia
C. Lobar pneumonia
D. Mitral stenosis
E. Bronchiectasis

1. Female patient, 60 years old. Cough accompanied by dyspnea. Physical examination: Dullness on percussion over the right lower lung, absent breath sounds in the right lower lung. The possible diagnosis for this patient is:

A. Spontaneous pneumothorax
B. Pleural effusion
C. Pericarditis
D. Bronchiectasis
E. Pulmonary emphysema

1. Male patient, 64 years old. Cardiac auscultation reveals a summation gallop. The possible diagnosis for this patient is:

A. Complete atrioventricular block
B. Atrial fibrillation
C. Cardiomyopathy with normal heart rate
D. Atrial premature beats
E. Heart failure with tachycardia

1. Male patient, chest tightness. Physical examination: Heart dullness borders are water-bottle shaped. The diagnosis for this patient is:

A. Pleural effusion
B. Left and right ventricular enlargement
C. Left and right atrial enlargement
D. Pericardial effusion
E. Left atrial enlargement

1. Female patient, 92 years old. Palpitations, shortness of breath. Physical examination: Diastolic thrill palpable over the apical area. The diagnosis for this patient is:

A. Aortic valve insufficiency
B. Pulmonary valve stenosis
C. Mitral stenosis
D. Aortic valve stenosis
E. Mitral valve insufficiency

1. Female patient, 76 years old. Physical examination: Heart dullness borders are smaller. The possible diagnosis for this patient is:

A. Pleural effusion
B. Massive ascites
C. Pulmonary consolidation
D. Pleural adhesions
E. Pulmonary emphysema

1. Male patient, 28 years old. Presents with right lower abdominal colicky pain, radiating to the right groin and lumbar region. The diagnosis for this patient is:

A. Acute enteritis
B. Acute appendicitis
C. Acute cholecystitis
D. Ureteral stone
E. Acute gastritis

1. Male patient, 77 years old. Upper abdominal distension and pain for 6 days, came for consultation this morning on an empty stomach. Physical examination revealed a succussion splash in the upper abdomen. The possible diagnosis for this patient is:

A. Normal
B. Large amount of gas in the abdominal cavity
C. Large amount of gas in the stomach
D. Large amount of fluid in the abdominal cavity
E. Large amount of fluid retention in the stomach

1. Male patient, 22 years old. After intravenous drip of penicillin for an upper respiratory tract infection, multiple purplish-red patchy lesions are seen on the skin, varying in size, slightly raised, not blanching on pressure, diameter about 5mm. Diagnosis is:

A. Purpura
B. Urticaria
C. Hematoma
D. Petechiae
E. Erythematous rash

1. Female patient, 45 years old. Chills, fever for 3 days, right upper quadrant pain for 1 day. Physical examination: Liver palpable 4cm below costal margin, soft, tender, with percussion pain, edges regular. The most likely diagnosis for this patient is:

A. Hepatitis
B. Liver abscess
C. Polycystic liver
D. Liver cancer
E. Hepatic echinococcosis

1. Female patient, 56 years old. History of diabetes for 15 years. 1 day ago, she developed coma, dyspnea, urine ketones (++++). The dyspnea in this patient belongs to:

A. Pulmonary origin
B. Cardiac origin
C. Toxic origin
D. Hematogenous origin
E. Neurogenic origin

1. Male patient, 22 years old. Admitted due to high fever, abdominal pain, diarrhea for 3 days, and dizziness for 1 day. Physical examination: T 39.4℃, flushed face, conjunctival congestion with hemorrhagic spots and edema, small petechiae on skin. BP 60/40mmHg, WBC 16×10⁹/L, Hb 162g/L, urine protein (+++). The cause of shock in this patient is:

A. Diarrhea
B. Increased vascular permeability
C. Hemoconcentration
D. Hemorrhage
E. Sepsis

1. Male patient, 67 years old. History of chronic atrophic gastritis for 30 years. In the past 6 months, irregular upper abdominal pain, poor appetite, weight loss, black stools. Physical examination: Anemic appearance, upper abdominal tenderness. The possible diagnosis for this patient is:

A. Gastric cancer
B. Liver cirrhosis
C. Peptic ulcer
D. Gallstones
E. Chronic pancreatitis

1. Male patient, 25 years old. Visited due to shortness of breath on exertion. Physical examination: Systolic ejection murmur heard at the left sternal border 3rd-4th intercostal spaces. Echocardiography shows ventricular septum and left ventricular posterior wall thickening, ratio >1.3. The most likely diagnosis is:

A. Ventricular septal defect
B. Hypertrophic cardiomyopathy
C. Coronary heart disease
D. Congenital heart disease, aortic stenosis
E. Dilated cardiomyopathy

1. Male patient, 28 years old. Periodic mid-upper abdominal pain for over 5 years. Often occurs when hungry, sometimes wakes up at night with pain, relieved by food, often with acid reflux and belching. In the past 2 days, tarry stools, accompanied by dizziness and fatigue. Physical examination: BP 90/60mmHg, HR 104 bpm. Soft abdomen, mild tenderness in upper abdomen, liver and spleen not palpable below costal margin, active bowel sounds. The most likely diagnosis is:

A. Gastric ulcer with bleeding
B. Duodenal bulb ulcer with bleeding
C. Gastric cancer with bleeding
D. Esophageal varices rupture and bleeding
E. Acute gastric mucosal lesions

1. Male patient, 25 years old. Came to emergency department with severe asthma attack. Physical examination: Patient has orthopnea, profuse sweating, speech is not coherent. The sign indicating a severe condition is:

A. Extensive high-pitched wheezing sounds in the lungs
B. Widened intercostal spaces
C. Hyperresonance on lung percussion
D. Decreased breath sounds in both lungs, occasional scattered wheezes
E. Decreased absolute cardiac dullness

1. Female patient, 26 years old. Recurrent oral ulcers, facial rash for 3 years. Lab tests: Peripheral blood leukopenia and thrombocytopenia, urine protein (+), ANA 1:640, C3 decreased. The most appropriate current diagnosis is:

A. Overlap syndrome
B. Behçet's disease
C. Systemic lupus erythematosus
D. Recurrent oral ulcer
E. Mixed connective tissue disease

1. Male patient, 69 years old. Lower limb edema 4 months ago. Hemoglobin 150g/L, urine routine protein positive, 24-hour urine protein quantification 5.9g, plasma albumin 19.2g/L, serum creatinine 108µmol/L. Now the patient has no lower limb edema, but ascites has significantly increased. Hemoglobin 180g/L, 24-hour urine protein quantification 4.8g, plasma albumin 16.7g/L, serum creatinine 268µmol/L. The first considered complication is:

A. Inferior vena cava thrombosis and embolism
B. Schistosomiasis
C. Peritonitis
D. Intestinal perforation
E. Liver cirrhosis

1. Male patient, young adult. Sudden headache for 2 hours, accompanied by nausea and vomiting. Physical examination: Motor aphasia, right limb hemiparesis, right pupil 3mm, left pupil 4mm, sluggish light reflex. The incorrect management is:

A. Perform cerebral angiography if condition allows
B. Keep patient's airway clear to prevent suffocation
C. Intravenous push 20% mannitol 250ml, while preparing for surgery
D. Immediate head CT scan
E. Lumbar puncture, cerebrospinal fluid routine examination

1. Patient with mitral stenosis. Physical examination: HR 102 bpm, diastolic thrill palpable at apex, opening snap audible at apex, ECG shows atrial fibrillation. The sign indicating good valve elasticity and mobility is:

A. Gallop rhythm
B. Pericardial knock
C. Splitting of heart sounds
D. Ejection sound
E. Opening snap

1. Female patient, 50 years old. Upper abdominal pain for over 3 months. 2 months ago, barium meal showed ulcer on the posterior wall of the gastric antrum. After 8 weeks of anti-ulcer medication, there was transient relief. The further preferred management is:

A. Gastroscopy
B. Repeated stool occult blood tests
C. Surgical treatment
D. Continue medication
E. Barium meal examination

1. Female patient, 51 years old. Swelling and pain in both wrist joints for 3 months. Swelling and pain in the 3rd and 4th interphalangeal joints of both hands, swelling and pain in the 2nd metacarpophalangeal joints of both hands for 2 months. Morning stiffness for 2 hours, low-grade fever. The most likely diagnosis is:

A. Systemic lupus erythematosus
B. Rheumatic arthritis
C. Osteoarthropathy
D. Rheumatoid arthritis
E. Spondyloarthropathy

1. The patient suddenly developed coma, convulsions, pinpoint pupils, cold clammy skin, excessive sweating, and dyspnea. The most likely diagnosis is:

A. CO poisoning
B. Barbiturate poisoning
C. Heat stroke
D. Atropine poisoning
E. Organophosphorus pesticide poisoning

1. Female patient, 28 years old. Found unconscious and in shock. There was a stove in the room, and empty bottles of dichlorvos were found. Physical examination: T 36℃, BP 90/60mmHg. Cold extremities, absent tendon reflexes. ECG shows first-degree AV block. Urine sugar (+), urine protein (+), blood COHb 60%. After diagnosing this condition, the primary treatment method is:

A. Hemodialysis
B. Hibernation therapy
C. Oxygen therapy
D. Energy mixture
E. Rapid intravenous drip of 20% mannitol 250ml

1. Male patient, 36 years old. Cough, purulent sputum, fever. Body temperature often above 39℃, temperature fluctuation within 24 hours does not exceed 1℃. Physical examination: Increased vocal fremitus over right upper lung, dullness on percussion, increased vesicular breath sounds. The most likely diagnosis for this patient is:

A. Lobar pneumonia
B. Pulmonary tuberculosis
C. Lung abscess
D. Chronic bronchitis
E. Bronchiectasis

1. Male patient, 33 years old. Fever, chest pain. Physical examination reveals pleural friction rub. The respiratory rhythm of this patient is:

A. Mainly abdominal breathing
B. Suppressed respiration
C. Biot's respiration
D. Cheyne-Stokes respiration
E. Sighing respiration

1. Fever patient, body temperature has remained at 39-41℃ in the last 5 days, temperature fluctuation within 24 hours does not exceed 1℃. Physical examination: Rose spots on abdomen, hepatosplenomegaly. The fever type of this patient is:

A. Intermittent fever
B. Undulant fever
C. Relapsing fever
D. Continuous fever
E. Remittent fever

1. Continuous fever is commonly seen in:

A. Lobar pneumonia
B. Malaria
C. Sepsis
D. Pulmonary tuberculosis
E. Rheumatic fever

1. Male patient, 28 years old. High fever for 5 days, then developed cough, accompanied by left chest pain, and expectorated rusty sputum. Possible diagnosis is:

A. Influenza
B. Lobar pneumonia
C. Pulmonary tuberculosis
D. Sepsis
E. Acute myocarditis

1. Male patient, 54 years old. Diagnosed with hepatitis B-related cirrhosis for 6 years, abdominal distension with bilateral lower limb edema for 1 month, worsened with anuria for 2 days. Physical examination: Frog belly, positive shifting dullness, pitting edema of both lower limbs. Lab tests: Blood sodium 122mmol/L, BUN 19mmol/L. The most likely current diagnosis is:

A. Rapidly progressive glomerulonephritis
B. Hepatic encephalopathy
C. Hepatorenal syndrome
D. Hepatopulmonary syndrome
E. Chronic renal failure

1. The mechanism unrelated to the formation of ascites in liver cirrhosis is:

A. Increased portal venous pressure
B. Hypoalbuminemia
C. Secondary hyperaldosteronism
D. Insufficient effective circulating volume
E. Increased capillary permeability

**B Type Questions**

(Questions 68-71 share the same answer choices)
A. Paroxysmal nocturnal dyspnea with pink frothy sputum
B. Blood-streaked sputum during severe coughing
C. Over 45 years old with a history of heavy long-term smoking, persistent blood-streaked sputum
D. Low-grade fever, night sweats, coughing fresh bloody sputum
E. Long-term cough, large amount of purulent sputum, recurrent massive hemoptysis

1. Characteristic of pulmonary tuberculosis is
2. Characteristic of lung cancer is
3. Characteristic of bronchiectasis is
4. Characteristic of acute left heart failure is

(Questions 72-76 share the same answer choices) Regarding associated symptoms for differential diagnosis of hematochezia:
A. Accompanied by cyclical abdominal pain
B. Accompanied by tenesmus
C. Accompanied by clusters of skin/mucosa telangiectasias
D. Accompanied by abdominal mass
E. Skin and mucosal bleeding

1. Characteristic of hereditary hemorrhagic telangiectasia is
2. Characteristic of rectal cancer and dysentery is
3. Characteristic of epidemic hemorrhagic fever is
4. Characteristic of intestinal malignant lymphoma is
5. Characteristic of peptic ulcer is

(Questions 77-81 share the same answer choices)
A. Coronary atherosclerosis stenosis ≥75%
B. Coronary atherosclerosis stenosis ≤50%
C. Coronary artery spasm
D. Complete occlusive thrombosis in the coronary artery
E. Coronary plaque rupture with incomplete thrombosis

1. Coronary atherosclerosis
2. Characteristic of variant angina pectoris is
3. Characteristic of acute myocardial infarction is
4. Characteristic of stable angina pectoris is
5. Characteristic of unstable angina pectoris is

(Questions 82-86 share the same answer choices)
A. Abnormal blood vessel wall function
B. Thrombocytopenia
C. Thrombocytopenia with coagulation dysfunction
D. Abnormal platelet function
E. Coagulation dysfunction

1. Bleeding in allergic purpura is due to
2. Bleeding in aplastic anemia is due to
3. Bleeding in disseminated intravascular coagulation is due to
4. Bleeding in thrombasthenia is due to
5. Bleeding in hemophilia is due to

(Questions 87-90 share the same answer choices)
A. Sudden, severe mid-epigastric cutting or burning pain
B. Persistent severe mid-epigastric pain or progressively worsening
C. Paroxysmal substernal colicky pain
D. Persistent, widespread severe abdominal pain with abdominal wall muscle rigidity or board-like rigidity
E. Right lower quadrant tenderness

1. Biliary ascariasis may present with
2. Acute diffuse peritonitis may present with
3. Acute gastritis or pancreatitis may present with
4. Duodenal ulcer perforation may present with

(Questions 91-95 share the same answer choices)
A. Undulant fever
B. Remittent fever
C. Intermittent fever
D. Irregular fever
E. Continuous fever

1. The typical fever type for typhoid fever is
2. The typical fever type for malaria is
3. The typical fever type for tuberculosis is
4. The typical fever type for brucellosis is
5. The typical fever type for suppurative inflammation is

(Questions 96-100 share the same answer choices)
A. Rotten apple odor
B. Urine-like odor
C. Fetor hepaticus
D. Pungent garlic odor
E. Strong alcohol odor

1. The respiratory odor in diabetic ketoacidosis patients is
2. The respiratory odor after alcohol consumption or in drunk individuals is
3. The respiratory odor in dichlorvos poisoning is
4. The respiratory odor in hepatic coma patients is
5. The respiratory odor in uremia patients is

**Relative Expertise**

1. The normal mitral valve orifice area is:

A. <1.0 cm²
B. 1.0-1.9 cm²
C. 2.0-3.0 cm²
D. 3.1-3.9 cm²
E. 4.0-6.0 cm²

1. The most important drug for improving symptoms of acute left heart failure is:

A. Diuretics
B. Digitalis
C. Calcium channel blockers
D. Beta-adrenergic receptor blockers
E. Angiotensin-converting enzyme inhibitors

1. Regarding the changes in the systolic murmur at the left sternal border in obstructive hypertrophic cardiomyopathy, the correct statement is:

A. It decreases when left ventricular outflow tract obstruction worsens.
B. It decreases during breath-holding.
C. It intensifies when left ventricular volume decreases.
D. It decreases when myocardial contractility increases.
E. It intensifies when squatting.

1. Female patient, 45 years old. Frequent substernal pain in the past 2 years, relieved by nitroglycerin each time, suspected coronary heart disease with angina pectoris. To confirm the diagnosis, the most economical and simple examination is:

A. ECG exercise stress test
B. Heart X-ray
C. Coronary angiography
D. Radionuclide examination
E. Dynamic ECG

1. The most common early arterial blood gas analysis finding in an acute asthma attack is:

A. Decreased PaO₂, increased PaCO₂, increased pH
B. Decreased PaO₂, increased PaCO₂, decreased pH
C. Normal PaO₂, increased PaCO₂, decreased pH
D. Decreased PaO₂, normal PaCO₂, decreased pH
E. Decreased PaO₂, decreased PaCO₂, increased pH

1. The following echocardiogram finding does *not* support the diagnosis of pulmonary heart disease:

A. Right ventricular outflow tract diameter ≥30mm
B. Right ventricular anterior wall thickening
C. Right ventricular internal diameter ≥20mm
D. Ratio of left/right ventricular internal diameters >2
E. Enlarged right pulmonary artery internal diameter and right atrium

1. The main clinical feature of bronchial asthma is:

A. Inspiratory dyspnea
B. Recurrent, paroxysmal, expiratory dyspnea
C. Recurrent, mixed dyspnea
D. Paroxysmal nocturnal dyspnea
E. Multiple wheezes in lungs with basal wet rales

1. Among community-acquired pneumonias, the main pathogen is:

A. Klebsiella pneumoniae
B. Haemophilus influenzae
C. Staphylococcus aureus
D. Streptococcus pneumoniae
E. Mycoplasma

1. Dull-tympanic percussion note on the chest can be seen in:

A. Cavitary pulmonary tuberculosis
B. Congestive phase of pneumonia
C. Pleural thickening
D. Pulmonary emphysema
E. Pneumothorax

1. The exophthalmos in non-infiltrative exophthalmos generally does not exceed (for females):

A. 1mm
B. 15mm
C. 16mm
D. 18mm
E. 19mm

1. The most common cause of upper gastrointestinal bleeding is:

A. Peptic ulcer
B. Acute gastric mucosal lesion
C. Esophageal and gastric varices
D. Gastric cancer
E. Gastritis

1. Among the following Helicobacter pylori detection methods, the preferred invasive method is:

A. Mucosal tissue staining
B. ¹⁴C urea breath test
C. Serum Hp antibody detection
D. Rapid urease test
E. Culture isolation method

1. When treating ascites in liver cirrhosis, it is generally *not* recommended to:

A. Have a low-protein diet
B. Frequently move around/walk
C. Have a low-salt diet
D. Use diuretics
E. Supplement albumin

1. Male patient, 40 years old. Cirrhosis after hepatitis C for 7 years. Significant weight loss, fatigue in the past 3 months, frequent nosebleeds, progressively worsening abdominal distension, oliguria. Collapsed 2 hours ago after activity. The most likely diagnosis is:

A. Hemorrhagic shock
B. Primary liver cancer
C. Liver cancer nodule rupture and bleeding
D. Hypoglycemic coma
E. Cirrhosis complicated by gastrointestinal bleeding

1. Female patient, 29 years old. Passed 9 tarry stools in 3 days, fainted this morning and sent to hospital. No previous history of upper abdominal pain or liver disease, no recent medication history. Physical examination: BP 80/50mmHg, P 130 bpm. The preferred measure is:

A. Oral acid-suppressing drugs
B. Fluid resuscitation
C. Gastric lavage with ice saline
D. Oral norepinephrine
E. Endoscopic hemostasis

1. Female patient, 63 years old. Suffered from latent glomerulonephritis for 12 years. Often has red blood cells in urine. This time visited due to urinary frequency, urgency, dysuria, and gross hematuria. Urinalysis: PRO (++), occult blood (+++), sediment RBC 25-30/HP, WBC 40-50/HP. Initial diagnosis is urinary tract infection. The test that most supports this diagnosis is:

A. Positive urinary tract irritation signs
B. Positive urine protein
C. Urinary red blood cell dysmorphic rate 20%-30%
D. Increased urinary white blood cells
E. Positive midstream urine bacterial culture

1. In chronic kidney disease stage 5, the glomerular filtration rate is at least less than:

A. 20 ml/min
B. 15 ml/min
C. 25 ml/min
D. 10 ml/min
E. 30 ml/min

1. The marker for the end of the oliguric phase in acute renal failure is when the 24-hour urine volume increases to at least:

A. 250 ml
B. 300 ml
C. 350 ml
D. 400 ml
E. 500 ml

1. The most common secondary kidney disease causing chronic renal insufficiency is:

A. Hepatitis B-associated glomerulonephritis
B. Amyloid nephropathy
C. Diabetic nephropathy
D. Benign renal arteriolosclerosis
E. Systemic lupus erythematosus

1. The drug that can control the progression of rheumatoid arthritis is:

A. Aspirin
B. Nonsteroidal anti-inflammatory drugs
C. Methotrexate
D. Prednisone
E. High-dose intravenous immunoglobulin

1. The primary first aid measure for a patient having an epileptic seizure is:

A. Rapidly administer medication to control the seizure
B. Press the philtrum (Renzhong point)
C. CT scan to find the cause
D. Maintain airway patency, prevent suffocation
E. Take a detailed history

1. Injection of MPTP (1-methyl-4-phenyl-1,2,3,6-tetrahydropyridine) can cause disease in humans and animals resembling:

A. Alzheimer's disease
B. Epilepsy
C. Parkinson's disease-like symptoms
D. Brain malformation
E. Congenital hydrocephalus

1. Symptoms that should *not* be present in idiopathic facial nerve palsy (Bell's palsy) are:

A. Loss of forehead wrinkles
B. Bell's phenomenon
C. Pain behind the ear or at the mandibular angle
D. Loss of taste on anterior 2/3 of tongue
E. Pain in the external auditory canal or tympanic membrane

1. For idiopathic generalized tonic-clonic seizures, the first-choice drug is:

A. Sodium valproate
B. Carbamazepine
C. Phenytoin sodium
D. Ethosuximide
E. Phenobarbital

1. The preferred treatment for rescuing a patient in status epilepticus is:

A. Chloral hydrate enema
B. Intravenous phenytoin sodium
C. Intravenous diazepam
D. Intramuscular chlorpromazine
E. Intramuscular phenobarbital sodium

1. The cause of Guillain-Barré syndrome may be:

A. Viral infection of peripheral nerves
B. Viral infection of the spinal cord
C. Autoimmune inflammation of the spinal cord
D. Autoimmune inflammation of peripheral nerves
E. Bacterial infection of peripheral nerves

1. Regarding the description of meningeal changes in tuberculous meningitis, the incorrect one is:

A. Congestion and exudation of the leptomeninges with formation of many tubercles.
B. Inflammation tends to accumulate in the subarachnoid space.
C. Direct spread from cranial or middle ear/mastoid tuberculous foci invading the meninges.
D. Exudate may show epithelioid cells, Langhans cells.
E. Large accumulation of exudate in the subarachnoid space.

1. The commonly used course of preventive chemotherapy for tuberculosis is:

A. 3-6 months
B. 6-9 months
C. 12-18 months
D. 18-24 months
E. 24-30 months

1. Female child, 8 years old. Fever, rash for 3 days, cough for 2 days. Physical examination: T 39.5℃, flushed face, maculopapular rash on trunk, enlarged tonsils, congested pharynx, bilateral lungs with scattered moist rales. The most likely diagnosis is:

A. Measles
B. Rubella
C. Drug eruption
D. Scarlet fever
E. Chickenpox

1. Male child, 3 years old. Diagnosed with systemic miliary tuberculosis. After treatment for a period, intracranial pressure increased with projectile vomiting, initially considered hydrocephalus. Based on pathological changes, the most likely cause is:

A. Cranial nerve damage
B. Brain parenchymal lesions
C. Hydrocephalus and ependymitis
D. Embolic endarteritis
E. Spinal cord lesions

1. The human immunodeficiency virus mainly attacks which cells in the human body?

A. T lymphocytes
B. B lymphocytes
C. Suppressor T cells
D. Helper T cells
E. Macrophages

1. The laboratory test that can be used for early diagnosis of epidemic encephalitis B is:

A. Complement fixation test
B. Hemagglutination inhibition test
C. Neutralization test
D. Specific IgM antibody detection
E. Virus isolation

1. The manifestations (typical) of malaria do *not* include:

A. Timed, periodic chills, fever, and sweating episodes
B. Complete remission intervals
C. Splenomegaly
D. Leukocytosis, elevated neutrophils
E. Anemia

1. The main pathological characteristic of typhoid fever is:

A. Swelling of small vascular endothelial cells
B. Myocardial necrosis
C. Bone marrow suppression
D. Hyperplastic reaction of the systemic mononuclear-phagocyte system
E. Extensive hepatocyte necrosis

1. Which provides protection against hepatitis B virus infection?

A. Anti-HBe
B. Anti-HBs
C. DNA polymerase
D. Antinuclear antibody
E. Anti-HBc

1. Male patient, 40 years old. Works in steelmaking. Suddenly fainted at work, discovered by colleagues and immediately sought medical attention. Physical examination: Flushed skin, normal pupil size, cherry-red lips. The most likely diagnosis is:

A. Atropine poisoning
B. Carbon monoxide poisoning
C. Ethanol poisoning
D. Organophosphorus pesticide poisoning
E. Sedative-hypnotic drug poisoning

1. For a patient with acute organophosphorus pesticide poisoning, the immediate measure to take is:

A. Terminate exposure to the poison and maintain vital signs
B. Gastric lavage to rapidly remove poison from the digestive tract
C. Catharsis to rapidly remove poison from the body
D. Oxygen therapy to protect brain tissue
E. Use specific antidote

1. Male patient, 30 years old. Suicide by drug ingestion, discovered and urgently sent to hospital. Physical examination: Comatose, rapid respiration, garlic odor, cold clammy skin, bilateral pupils pinpoint size. The preferred antidote is:

A. Pralidoxime iodide
B. Atropine
C. Obidoxime
D. Pralidoxime chloride
E. Mannitol

**A3/A4 Type Questions**

(Questions 39-41 share the same case description)
Female patient, 63 years old. For 3 years, palpitations and shortness of breath on exertion, sometimes waking up at night short of breath, cough. For 1-year, abdominal distension, oliguria, edema. Has been taking furosemide and digoxin. Symptoms worsened 1 week ago after an upper respiratory tract infection, with palpitations and loss of appetite. Physical examination: BP 130/80mmHg. Enlarged heart border, diastolic rumbling murmur and grade 3/6 systolic blowing murmur at the apex, HR 68 bpm, premature beats, bigeminy, few moist rales at lung bases. Distended neck veins, liver palpable 3cm below costal margin, spleen not palpable, bilateral lower limb edema.

1. The diagnosis for this case is:

A. Congenital heart disease
B. Rheumatic heart disease
C. Coronary heart disease
D. Dilated cardiomyopathy
E. Senile degenerative heart valve disease

1. ECG showed ventricular bigeminy. The most likely cause of this arrhythmia is:

A. Myocardial ischemia
B. Heart failure
C. Electrolyte disorder
D. Digitalis toxicity
E. Infection

1. The treatment measure for this case is:

A. Continue the above treatment, add lidocaine
B. Continue the above treatment, add metoprolol
C. Continue digitalis, stop diuretics
D. Stop digitalis, supplement potassium
E. Continue the above treatment, strengthen anti-infection

(Questions 42-44 share the same case description)
Male patient, 74 years old. 20 years ago, began having paroxysmal dizziness, headache, occasional BP up to 170/105mmHg. Did not take antihypertensive medication. In the past 3 years, physical strength gradually declined, exertional dyspnea relieved by rest, occasional bilateral lower limb edema. Half an hour ago, after lifting a heavy object, sudden headache, blurred vision, palpitations, shortness of breath, inability to lie flat, profuse sweating, took nifedipine without relief. Denies history of diabetes, coronary heart disease. Physical examination: P 130 bpm, BP 260/130mmHg, R 36 bpm. Sitting position, cold clammy skin, cyanotic lips, no neck vein distension, dense bubbling rales in lower lung fields, expiratory wheezes. Heart enlarged to left lower, regular rhythm, grade 2/6 systolic blowing murmur at apex, ventricular gallop. Soft abdomen, no lower limb edema. Auxiliary tests: Head CT shown no significant abnormality, urine routine RBC(+++), renal blood flow reduced bilaterally, especially right more than left, right kidney size 5.6cm×8.5cm, left kidney size 10cm×7cm.

1. The patient's diagnosis is:

A. Coronary heart disease, cardiac insufficiency
B. Dilated cardiomyopathy, cardiac insufficiency
C. Hypertension, cardiac insufficiency
D. Pheochromocytoma, acute left heart failure
E. Renal artery stenosis, acute left heart failure

1. The "gold standard" for diagnosing this disease is:

A. Adrenal enhanced CT
B. Renal vascular ultrasound
C. Renal angiography
D. Cardiac ultrasound
E. Plasma renin, angiotensin, aldosterone measurement

1. After treatment, the patient's dyspnea resolved. The relatively contraindicated drug for this disease is:

A. Beta-blockers
B. Alpha-blockers
C. Diuretics
D. Calcium channel blockers
E. ACEI class

(Questions 45-47 share the same case description)
Male patient, 70 years old. History: Cough, sputum for 30 years, shortness of breath on activity for 10 years, bilateral lower limb edema in the last year. Pulmonary function shows obstructive ventilation disorder, 50% of predicted value.

1. The type of obstructive emphysema in this patient is mostly:

A. Centrilobular
B. Panlobular
C. Mixed
D. Paraseptal
E. Interstitial

1. The main factor for the development of pulmonary hypertension in this patient is:

A. Reduction of pulmonary capillary bed
B. Increased blood viscosity
C. Increased blood volume
D. Formation of microemboli in pulmonary capillaries
E. Hypoxia and carbon dioxide retention causing pulmonary arteriolar spasm

1. Which pulmonary function test is most valuable for the diagnosis of obstructive emphysema?

A. Tidal volume
B. Vital capacity
C. Arterial oxygen partial pressure
D. Residual volume and residual volume/total lung capacity (%)
E. Minute ventilation at rest

(Questions 48-50 share the same case description)
Female patient, 32 years old. Dry cough, low-grade fever for over 1 month. Chest X-ray shows scattered nodular shadows in both lower lungs. Blood WBC and differential normal. Repeated use of multiple antibiotics, clinical presentation and lab tests show no significant change. Physical examination no abnormality found.

1. The test that is less meaningful for diagnosis is:

A. Lung biopsy
B. Bronchoalveolar lavage
C. Chest HRCT
D. Sputum culture
E. Cold agglutinin test

1. The patient's cerebrospinal fluid India ink stain smear microscopy revealed round pathogens with transparent capsules. The most likely diagnosis is:

A. Candida albicans infection
B. Pneumocystis infection
C. Cryptococcus neoformans infection
D. Pneumocystis carinii infection
E. Fungal infection

1. The preferred treatment drug is:

A. Flucytosine
B. Tetracycline
C. Amphotericin B
D. Fluconazole
E. Glucocorticoids

(Questions 51-52 share the same case description)
Male patient, 35 years old. Thirst, polydipsia, polyuria for 1 month. Fasting blood glucose 18mmol/L, urine sugar (+++), ketones (±); his mother has diabetes for 5 years. Physical examination: Height 167cm, Weight 75kg, heart, lungs, abdomen (-).

1. To clarify the type of diabetes, the tests to be done include:

A. Fasting insulin measurement
B. Blood ketone measurement
C. Oral glucose tolerance test
D. ICA, GAD
E. Glucagon stimulation test

1. Based on this patient's condition, the best treatment plan is:

A. Short-acting insulin 3 times + intermediate-acting insulin
B. Intermediate/Long-acting insulin + sulfonylurea oral hypoglycemic
C. Sulfonylurea oral hypoglycemic + glucosidase inhibitor
D. Sulfonylurea oral hypoglycemic + biguanide oral hypoglycemic
E. Short/Intermediate mixed insulin + biguanide oral hypoglycemic

(Questions 53-55 share the same case description)
Male patient, 31 years old. Sudden severe abdominal pain 3 hours after department dinner yesterday, nausea, vomiting, vomited large number of gastric contents. 3 months ago, company physical found slightly enlarged gallbladder. Physical examination: T 38℃, P 95 bpm, BP 98/65mmHg. Flat soft abdomen, mild tenderness at xiphoid, Murphy's sign positive. Blood WBC 10.5×10⁹/L, segmented neutrophils 0.88, blood amylase 1000 Somogyi units.

1. The most likely diagnosis for this patient is:

A. Duodenal bulb ulcer with pyloric obstruction
B. Gastric ulcer with perforation
C. Acute edematous pancreatitis and cholecystitis
D. Acute incomplete intestinal obstruction
E. Chronic calculous cholecystitis

1. The preferred rapid examination method for confirming the diagnosis is:

A. Endoscopic ultrasound
B. Upper abdominal CT
C. B-ultrasound
D. Full gastrointestinal barium contrast
E. ERCP

1. The most ideal treatment method is:

A. Supportive treatment
B. Traditional Chinese medicine treatment
C. Anti-infective treatment
D. Endoscopic treatment
E. Active symptomatic and supportive therapy, elective cholecystectomy after acute symptoms resolve

(Questions 56-58 share the same case description)
Male patient, 55 years old. Ulcer history 15 years. Pain gradually worsened in the past 3 months, no rhythmicity, medication ineffective. Physical examination: No enlarged superficial lymph nodes, flat soft abdomen, upper abdominal tenderness, palpable mass.

1. The preferred examination is:

A. Serum gastrin measurement
B. Gastroscopy
C. Abdominal B-ultrasound
D. Gastrointestinal contrast
E. Abdominal CT

1. The most likely current diagnosis is:

A. Gastric ulcer with scar formation
B. Gastrinoma
C. Gastric ulcer with pyloric obstruction
D. Gastric ulcer perforation
E. Gastric ulcer canceration

1. The preferred treatment to consider is:

A. Radiofrequency therapy
B. Endoscopic lesion resection
C. Radiotherapy
D. Chemotherapy
E. Surgical treatment

(Questions 59-61 share the same case description)
Male patient, 60 years old. Gastric history 8 years. Upper abdominal pain in the past 3 months, accompanied by decreased appetite and weight loss.

1. Gastroscopy showed pale, thin mucosa, visible vessels. Hp test negative. Diagnosis should be:

A. Gastric ulcer
B. Compound ulcer
C. Gastric cancer
D. Chronic superficial gastritis
E. Chronic atrophic gastritis

1. Regarding the pathological histology of this patient, the incorrect description is:

A. Partial or complete atrophy of gastric glands
B. Inflammatory cell infiltration only in superficial mucosal layer
C. Common pyloric gland metaplasia
D. Severe intestinal metaplasia
E. Often accompanied by dysplasia

1. The chosen management measure is:

A. Surgical treatment
B. Prevent complications
C. Antibiotic treatment
D. Gastric acid analysis
E. Symptomatic treatment, regular gastroscopy follow-up

(Questions 62-63 share the same case description)
Female patient, 25 years old. Joint pain, bilateral lower limb edema for 2 weeks. Urine protein 4g/d, urine sediment RBC 10-20/HP, WBC 5-10/HP, urine culture negative, serum creatinine 102µmol/L, ANA (+).

1. The indicator still needed to diagnose SLE is:

A. Positive anti-Sm antibody
B. Positive anti-SSA antibody
C. Positive anti-RNP antibody
D. Positive anti-GBM antibody
E. Positive ANCA

1. The main significance of renal biopsy in this patient is:

A. Determine if it is lupus nephritis
B. Clarify the type of lupus nephritis and its pathological activity indicators
C. Whether it is combined with small vessel vasculitis
D. Whether there is an overlap syndrome
E. Whether acute renal failure is due to combined acute tubular necrosis

(Questions 64-66 share the same case description)
Female patient, 24 years old. Gingival bleeding with menorrhagia for 1-year, scattered petechiae and ecchymoses on lower limb skin. Hb 72g/L, WBC 4.6×10⁹/L, PLT 42×10⁹/L.

1. The most likely diagnosis is:

A. Acute leukemia
B. Allergic purpura
C. Idiopathic thrombocytopenic purpura
D. Aplastic anemia
E. Iron deficiency anemia

1. After half a year of hormone therapy, the effect is poor. The first consideration should be:

A. Increase glucocorticoid dose and continue
B. Switch to danazol
C. Antifibrinolytic therapy
D. Plasma exchange
E. Splenectomy

1. If the patient develops severe bleeding during treatment, the preferred treatment is:

A. Cimetidine
B. Yunnan Baiyao
C. High-dose intravenous immunoglobulin
D. Corticosteroids
E. Vincristine

(Questions 67-69 share the same case description)
Female patient, 26 years old. Gingival bleeding with fatigue for 7 days, skin patchy ecchymoses in the past 2 days. Lab tests: WBC 3.4×10⁹/L, PLT 25×10⁹/L, Hb 70g/L, plasma fibrinogen 1.5g/L; bone marrow nucleated cells hyperplastic, abnormal promyelocytes 0.85, cytoplasm filled with coarse, purplish red azurophilic granules, visible Auer rods.

1. The main cause of bleeding in this patient is:

A. ITP
B. Thrombocytopenia
C. Coagulation factor deficiency
D. Thrombocytopenia with dysfunctional platelets
E. Platelet reduction and coagulation abnormalities caused by acute promyelocytic leukemia

1. The preferred treatment regimen is:

A. Low-dose cytarabine
B. DA regimen
C. HA regimen
D. DA regimen + low-dose heparin
E. Tretinoin + low-dose heparin

1. The chromosomal gene finding that matches this patient is:

A. t (8;21) (q22; q22)
B. t (15;17) (q22; q21)
C. t (6;9) (p23; q34)
D. t (8;14) (q24; q32)
E. t (9;22) (q34; q11)

(Questions 70-72 share the same case description)
Female patient, 24 years old. Visited due to sore throat for 2 weeks, gross hematuria for 1 day. Physical examination: BP 150/100mmHg, anemic appearance, bilateral lower limb edema. Auxiliary tests: Urinalysis: urine protein (+++), red blood cells full field, white blood cells 5-8/HP, normal renal function. Serum complements significantly decreased, ASO normal, ANA(+). Color ultrasound shows both kidneys normal size.

1. The most likely diagnosis for this patient is:

A. Lupus nephritis
B. Acute poststreptococcal glomerulonephritis
C. Rapidly progressive glomerulonephritis
D. Mesangiocapillary glomerulonephritis
E. Acute exacerbation of chronic glomerulonephritis

1. The most guiding examination for treatment is:

A. Bone marrow search for LE cells
B. Skin lupus band test
C. Renal biopsy
D. Anti-Sm antibody and anti-dsDNA antibody detection
E. Anticardiolipin antibody test

1. Assuming renal pathology shows 60% crescent formation, the most appropriate treatment plan is:

A. Full-dose steroids combined with mycophenolate mofetil orally
B. Plasma exchange alone
C. Hydroxychloroquine supplemented with NSAIDs
D. Methylprednisolone pulse therapy
E. Oral steroids combined with cyclophosphamide

(Questions 73-75 share the same case description)
Male patient, 65 years old. Inability to move left limb for 2 hours. Physical examination: BP 150/90mmHg. Clear consciousness, pupils equal and round, showing teeth reveals mouth deviated to right, left upper and lower limb muscle strength grade II, positive pathological reflex. Head CT no abnormality found.

1. The diagnosis considered is:

A. Cerebral infarction
B. Acute myelitis
C. Subarachnoid hemorrhage
D. Cerebral venous sinus thrombosis
E. Cerebral hemorrhage

1. The first treatment to consider is:

A. Dehydration to lower intracranial pressure
B. Antihypertensive treatment
C. Glucocorticoid treatment
D. Antibiotics to prevent infection
E. Thrombolytic therapy

1. The lesion is likely located at:

A. Right cerebral frontal-parietal lobe
B. Left cerebral frontal-parietal lobe
C. Right frontal pole
D. Right occipital lobe
E. Right cerebellar hemisphere

(Questions 76-78 share the same case description)
Male patient, 32 years old. Progressive limb weakness for 8 days, accompanied by choking on swallowing. Physical examination: Clear consciousness, low hoarse voice with nasal tone, bilateral poor palatal elevation, absent gag reflex, soft neck, low limb muscle tone, muscle strength I-II, limb tendon reflexes absent, decreased pinprick sensation below elbows/knees bilaterally, no plantar response.

1. The first considered diagnosis is:

A. Generalized myasthenia gravis
B. Guillain-Barré syndrome
C. Brainstem thrombosis
D. Periodic paralysis
E. Polymyositis

1. The main auxiliary test that aids diagnosis is:

A. Head CT
B. Serum creatine kinase
C. ECG and blood potassium
D. Neostigmine test
E. Cerebrospinal fluid examination

1. The lesion site in this disease is at:

A. Spinal cord
B. Skeletal muscle
C. Neuromuscular junction
D. Peripheral nerves
E. Brainstem

(Questions 79-80 share the same case description)
Female patient, 35 years old. Usually asymptomatic, HBsAg (+) found on physical examination 3 years ago. To clarify whether hepatitis B exists.

1. The most meaningful examination is:

A. HBV-DNA, anti-HBc-IgM measurement
B. Liver/spleen B-ultrasound
C. AFP, GGT, AKP measurement
D. Serum fibrosis markers (MAO, type III procollagen, etc.)
E. HBV serum markers and ALT

1. Abnormal findings at outpatient clinic, admitted. Liver biopsy showed piecemeal necrosis of hepatocytes, portal area destruction, and passive septum formation. The pathological diagnosis is:

A. Early cirrhosis
B. Chronic persistent hepatitis
C. Chronic severe hepatitis
D. Subacute severe hepatitis
E. Chronic active hepatitis

(Questions 81-84 share the same case description)
Male patient, 45 years old. Visited in mid-December, fever for 4 days, temperature 38-39.5℃, chills, headache, body aches, poor appetite, abdominal distension, sore throat, mild cough. Physical examination: Clear consciousness, flushed skin on face and upper chest, 2-3 petechiae in axillae, conjunctival injection, heart/lungs normal, liver/spleen not palpable, kidney area percussion pain (+). BP 80/60mmHg, urine protein (+++), red/white blood cells each 3-5/HP, granular casts 1-2/HP. Hb 156g/L, WBC 14.8×10⁹/L, N 0.79, L 0.21.

1. To confirm the diagnosis, the preferred examination is:

A. Specific IgM antibody test for epidemic hemorrhagic fever virus
B. Blood culture
C. Widal reaction and Weil-Felix reaction
D. Chest X-ray
E. Microscopic agglutination test

1. The first treatment measure to adopt is:

A. High-dose antibiotics for infection
B. Vasoactive drugs to raise blood pressure
C. Expand blood volume
D. Adrenal corticosteroids
E. Heparin anticoagulation

1. The main reason for kidney injury is:

A. Type I hypersensitivity
B. Type II hypersensitivity
C. Type III hypersensitivity
D. Type IV hypersensitivity
E. Type V hypersensitivity

1. The current cause of shock is:

A. Toxic shock
B. Septic shock
C. Hypovolemic shock
D. Cardiogenic shock
E. Neurogenic shock

(Questions 85-88 share the same case description)
Male patient, 48 years old, farmer. History: diabetes, hypertension, depression. Found unconscious at home by family, brought to emergency. Deeply comatose, profuse sweating, vomitus on head/face with rotten garlic odor. Obvious tremor of facial and limb muscles. Pinpoint pupils equal and small, rapid respiration, cyanotic lips, HR 180 bpm, BP 180/120mmHg, extensive wet rales in both lungs. Family found multiple empty pesticide bottles of varying sizes/colors, no labels.

1. The most likely diagnosis for this patient is:

A. Diabetic ketoacidosis
B. Hypoglycemic coma
C. Organophosphorus pesticide poisoning
D. Antidepressant poisoning
E. Massive cerebral hemorrhage

1. The immediate emergency treatment measure is:

A. Rapid IV drip of 20% mannitol
B. IV hypertonic glucose
C. Gastric lavage and IV atropine and pralidoxime chloride
D. IV insulin drip
E. Gastric lavage and IV naloxone drip

1. If gastric lavage is needed, the lavage solution for this patient should be:

A. Clear water
B. 1:5000 potassium permanganate solution
C. 5% sodium sulfate
D. 2% sodium bicarbonate solution
E. 0.3% hydrogen peroxide solution

1. After active rescue, the patient regained consciousness, condition stabilized. When treated with the original dosage, developed dilated pupils, confusion, restlessness, convulsions, and coma again. This should be considered as:

A. Worsening cerebral hemorrhage, cerebral herniation
B. Excessive insulin dose, hypoglycemic coma
C. Enhanced toxicity after drug metabolism in the body
D. Hepatic encephalopathy
E. Atropine poisoning

**B Type Questions**

(Questions 89-92 share the same answer choices)
A. Heart failure
B. Ventricular arrhythmia
C. Pulmonary edema
D. Embolism
E. Atrial fibrillation

1. The most common clinical manifestation of rheumatic heart disease with mitral stenosis is
2. The common cause of death in infective endocarditis is
3. The most important late complication of aortic insufficiency is
4. The most serious complication of rheumatic heart disease with mitral stenosis is

(Questions 93-95 share the same answer choices)
A. Alveolar cell carcinoma
B. Squamous cell carcinoma
C. Small cell undifferentiated carcinoma
D. Adenocarcinoma
E. Large cell undifferentiated carcinoma

1. Endocrine disorder syndrome is more common in
2. Clubbing or hypertrophic osteoarthropathy is more common in
3. Grows relatively slowly, metastasizes later, high surgical resection rate is

(Questions 96-100 share the same answer choices)
A. Fluid infusion
B. Intravenous glucocorticoids
C. Intravenous 50% glucose
D. Immediate thyroid hormone (TH) replacement
E. Immediate propylthiouracil (PTU)

1. The primary and crucial measure for rescuing diabetic ketoacidosis (DKA) is
2. The main measure for rescuing Addisonian crisis is
3. The main measure for rescuing hypoglycemic coma is
4. The primary measure for rescuing myxedema coma is
5. The primary measure for rescuing thyroid storm is

**Foundational Professional Knowledge**

1. The sputum characteristic in acute pulmonary edema is:

A. Purulent
B. Rust-colored
C. Brownish red
D. Pink frothy
E. Chocolate-like

1. The clinical situation most likely to cause acute left heart failure is:

A. Frequent ventricular premature beats
B. Mitral valve chordae tendineae rupture
C. Grade 1 hypertension
D. Recurrent pulmonary embolism
E. Chronic persistent atrial fibrillation

1. The preferred examination for diagnosing heart failure is:

A. Chest X-ray
B. Echocardiography
C. Left ventriculography
D. Exercise stress test
E. Electrocardiogram

1. The definitive treatment for dilated cardiomyopathy is:

A. Implanting a DDD pacemaker
B. Rest and vasodilators
C. Cardiotonic agents
D. Medication, rest, and low-salt diet
E. Heart transplantation

1. The main clinical manifestation of dilated cardiomyopathy is:

A. Congestive heart failure
B. Sudden death
C. Embolism
D. Loss of appetite
E. Pulmonary infection

1. Which of the following belongs to a secondary cardiomyopathy?

A. Dilated cardiomyopathy
B. Hypertrophic cardiomyopathy
C. Restrictive cardiomyopathy
D. Hyperthyroid heart disease
E. Obstructive hypertrophic cardiomyopathy

1. For a hypertensive patient experiencing variant angina, the drug of choice is:

A. Nifedipine
B. Metoprolol
C. Nitroglycerin
D. Isosorbide dinitrate
E. Furosemide

1. The clinical manifestations of stable angina do *not* include:

A. Pressure or squeezing sensation in the precordium or behind the sternum
B. Lasting several minutes, relieved by stopping activity or sublingual nitroglycerin
C. Pain is dull in nature
D. No radiating pain
E. Severe symptoms may be accompanied by sweating

1. Regarding the pathogenesis of stable angina, the incorrect statement is:

A. Most of the oxygen carried by venous blood through the coronary circulation is extracted and utilized by the myocardium.
B. The myocardial oxygen demand and coronary artery oxygen supply are in balance; it is difficult to meet the metabolic demands by increasing oxygen extraction when myocardial oxygen consumption increases.
C. Oxygen carrying capacity can be increased by increasing blood flow.
D. Caused by reduced coronary blood flow or increased myocardial oxygen demand.
E. Myocardial hypoxia occurs whenever the myocardial oxygen supply-demand balance is disrupted.

1. Nonparoxysmal junctional tachycardia is most seen in:

A. Digitalis toxicity
B. Inferior wall myocardial infarction
C. Myocarditis
D. Increased endogenous catecholamines
E. Normal individuals

1. The arrhythmia that does *not* belong to irregular heart rhythms is:

A. Second-degree atrioventricular block
B. Third-degree atrioventricular block
C. Ventricular fibrillation
D. Atrial flutter with fixed AV conduction ratio
E. Atrial fibrillation

1. The ECG finding consistent with second-degree type I AV block is:

A. Both PP and RR intervals are irregular
B. Successive PR intervals progressively shorten
C. The RR interval containing the blocked P wave is less than twice the normal PP interval
D. The most common conduction ratio is 4:2
E. The PR interval preceding the blocked P wave is shortened

1. Regarding the description of normal sinus rhythm, the incorrect one is:

A. Impulse originates from the sinoatrial node
B. Rate 60-100 beats/min
C. P wave upright in leads I, II, aVF, inverted in aVR
D. PR interval 0.12-0.20 seconds
E. Heart rate is regular

1. The ECG change that does *not* conform to acute pericarditis is:

A. ST segment elevation concave upward
B. T wave flattening or inversion
C. QRS low voltage
D. Electrical alternans
E. ST segment elevation convex upward

1. The main feature of acute cardiac tamponade is:

A. Distended neck veins
B. Beck's triad
C. Muffled heart sounds on auscultation
D. Weak pulse on palpation
E. Decreased systolic BP, unchanged diastolic BP

1. The typical sign of fibrinous pericarditis is:

A. Ewart's sign
B. Pulsus paradoxus
C. Enlarged cardiac border
D. Pericardial friction rub
E. Kussmaul's sign

1. Regarding the description of aortic dissection as a complication of primary hypertension, the incorrect one is:

A. Aortic dissection is one cause of sudden death.
B. Tachycardia and decreased blood pressure during pain episodes.
C. Sudden severe tearing chest pain is the main manifestation.
D. Can rapidly lead to dissection rupture.
E. Hypertension is an important factor leading to aortic dissection.

1. In the diagnostic evaluation of primary hypertension, the standard used for risk stratification does *not* include:

A. Level of elevated blood pressure
B. Presence of various cardiovascular risk factors affecting prognosis
C. Presence of target organ damage
D. Alcohol consumption
E. Presence of related clinical complications

1. The blood pressure in isolated systolic hypertension is:

A. Systolic ≥140 mmHg, Diastolic <90 mmHg
B. Systolic >140 mmHg, Diastolic <90 mmHg
C. Systolic >130 mmHg, Diastolic <100 mmHg
D. Systolic >130 mmHg, Diastolic <90 mmHg
E. Systolic ≥130 mmHg, Diastolic <90 mmHg

1. The diagnostic standard for hypertension is systolic and/or diastolic blood pressure:

A. ≥140/90 mmHg
B. ≥130/80 mmHg
C. ≥130/85 mmHg
D. ≥139/89 mmHg
E. ≥120/80 mmHg

1. The sign that does *not* conform to a large pericardial effusion is:

A. Distended neck veins
B. Weakened or absent apical impulse
C. Increased pulse pressure, water-hammer pulse
D. Distant heart sounds
E. Hepatomegaly, ascites

1. The complication prone to occur after atrial fibrillation is:

A. Severe heart failure
B. Pulmonary infection
C. Confusion, convulsions
D. Systemic arterial embolism
E. Cardiogenic shock

1. Female patient, 82 years old. Recurrent chest tightness for over 20 years, bilateral lower limb edema for 1 week. Physical examination: Distended neck veins, shifting dullness in abdomen, pitting edema of lower limbs. Abdominal ultrasound suggests slightly enlarged liver, medium texture, smooth surface. The most likely diagnosis is:

A. Cirrhotic ascites
B. Liver cancer with ascites
C. Tuberculous peritonitis
D. Nephrotic syndrome with ascites
E. Right heart failure, hepatic congestion with ascites

1. Female patient, 51 years old. Rheumatic heart disease with mitral stenosis and atrial fibrillation, long-term digoxin 0.25mg/d. 10 days ago, developed abdominal distension, loss of appetite without obvious cause, accompanied by bilateral lower limb edema, later worsened with facial edema, progressively worse loss of appetite. In the past week, developed diarrhea, nausea, vomiting, poor food intake. Examination: Supine, conscious, apathetic, lungs clear, HR 46 bpm, diastolic rumbling murmur audible at apex. The direct reason the patient came for consultation is:

A. Mitral stenosis
B. Diarrhea
C. Reduced food intake
D. Hypovolemia
E. Digitalis toxicity

1. Female patient, 24 years old. Found grade 3 systolic murmur at cardiac apex 3 years ago, fever for 2 weeks. Systolic mid-late click audibles, clubbing of fingers, painless small petechiae on soles. The diagnosis for this case is:

A. Rheumatic heart disease, mitral insufficiency with subacute infective endocarditis
B. Sepsis
C. Mitral valve prolapses
D. Systemic lupus erythematosus
E. Rheumatic heart disease, active rheumatic fever

1. Male patient, 77 years old. History of COPD for several years, exertional chest tightness and shortness of breath for over 10 years. Paroxysmal nocturnal dyspnea appeared 6 days ago, no cough or sputum. Physical examination: BP 130/90mmHg, HR 98 bpm. No neck vein distension, moist rales at lung bases, no lower limb edema. Lung CT suggests bilateral pleural effusion, enlarged heart. The most likely cause of this episode of breathlessness is:

A. Pneumonia
B. Left heart failure
C. Right heart failure
D. Myocarditis
E. Bronchial asthma

1. Female patient, 69 years old. Recurrent chest tightness and shortness of breath for over 1 year. Worsened 1 month ago after a cold, accompanied by cough, sputum, worse at night. 4 hours ago, sudden worsening of shortness of breath, accompanied by cough, pink frothy sputum, inability to lie flat. Hypertension history over 15 years. Physical examination: BP 170/100mmHg. HR 110 bpm, regular, dry and fine moist rales audible in both lungs. The incorrect treatment measure is:

A. Intravenous furosemide
B. Intravenous metoprolol
C. Intravenous sodium nitroprusside drip
D. Intravenous morphine
E. High-flow oxygen

1. The main basis for diagnosing fibrinous pericarditis is:

A. Cardiac dullness borders enlarged to both sides, absolute dullness
B. Weak apical impulse, located medial to the left border of cardiac dullness or impalpable
C. Precordial pain
D. Dyspnea
E. Pericardial friction rub

1. Male patient, 59 years old. Recurrent chest tightness, palpitations, sudden onset, each episode lasting half an hour to 1 hour, relieved by belching, occurs 3-4 times per year. 10 days ago, sudden palpitations again, visited local hospital. ECG indicated supraventricular tachycardia. The heart rate during tachycardia in this patient is generally:

A. 60-100 bpm
B. 150-250 bpm
C. 200-300 bpm
D. 250-300 bpm
E. 300-350 bpm

1. Female patient, 28 years old. Palpitations on exertion for 4 years, occasional precordial pain. Physical examination: BP 140/40mmHg, heart border enlarged to left lower, diastolic decrescendo murmur heard at left sternal border 3rd intercostal space. The most likely diagnosis is:

A. Myocarditis
B. Patent ductus arteriosus
C. Obstructive hypertrophic cardiomyopathy
D. Coronary heart disease, angina pectoris
E. Rheumatic heart disease, aortic insufficiency

1. Male patient, 18 years old. Recurrent palpitations with fatigue for over 1-year, sudden onset and termination, each episode lasting several minutes to over ten minutes, no chest tightness or shortness of breath, can stop suddenly after breath-holding. Palpitations recurred 1 day ago, persisted over ten minutes without relief, visited local hospital. Symptoms improved after IV cedilanid 0.2mg slow injection. The mechanism of cedilanid injection during palpitations in this patient is:

A. Suppress reentry within the sinus and AV nodes
B. Suppress reentry within the AV node
C. Slow conduction in most cardiac tissues
D. Prolong repolarization in most cardiac tissues
E. Slow accessory pathway conduction, reduce automaticity

1. Male patient, 56 years old. Recurrent chest tightness, palpitations for over 5 years, recurrence for 6 days. Physical examination: BP 115/78mmHg, HR 70 bpm. Distended neck veins, hepatomegaly, enlarged heart border, muffled heart sounds, moist rales at lung bases, bilateral lower limb edema. Cardiac ultrasound: EF 21%, all cardiac chambers enlarged, mainly left ventricle, diffuse hypokinesis. The unsuitable treatment for this patient is:

A. Metoprolol
B. Furosemide
C. Spironolactone
D. Sacubitril/valsartan
E. Diltiazem

1. Female patient, 67 years old. Exertional left chest pain for 5 years, radiating to left shoulder, episodes last 3-4 minutes, relieved by rest. Fainted suddenly this afternoon during labor, emergency visit. Physical examination: BP 90/50mmHg, conscious, HR 140 bpm, systolic ejection murmur with thrill audibles over aortic area, radiating to neck, clear lung sounds. The first considered diagnosis is:

A. Aortic dilation
B. Aortic stenosis
C. Aortic atherosclerosis
D. Hypertension
E. Aortic insufficiency

1. Female patient, 51 years old. Chest tightness, fatigue for 3 years, worsened for 15 days. Cardiac ultrasound: EF 28%, LV end-diastolic diameter 7.8cm, diffuse hypokinesis of LV wall. Coronary CTA showed no significant stenosis. If the patient has dilated cardiomyopathy, the manifestation generally *not* present is:

A. Grade 3/6 rough holosystolic blowing murmur at apex
B. Elevated BNP or NT-proBNP
C. Systolic ejection murmur over aortic area
D. Pulmonary moist rales
E. Distended neck veins

1. Male patient, 68 years old. Physical examination: Diastolic decrescendo murmur at heart base, loudest at right sternal border 2nd intercostal space, S2 accentuated, BP 170/90mmHg. Chest X-ray: widened tortuous aorta, boot-shaped heart shadow. The most likely diagnosis is:

A. Rheumatic aortic insufficiency
B. Congenital heart disease, bicuspid aortic valve
C. Aortic atherosclerosis, aortic insufficiency
D. Pulmonary hypertension, relative pulmonary insufficiency
E. Hypertensive heart disease

1. Female patient, 42 years old. Recurrent amaurosis fugax, chest tightness for over 10 years, worsened for 20 days. Poor effect with nitroglycerin during episodes. Physical examination: BP 99/50mmHg, HR 78 bpm. Rough systolic ejection murmur audible at left sternal border 3rd-4th intercostal spaces, intensifies when standing. Cardiac ultrasound: Normal LV cavity, IVS thickness 27mm, LV posterior wall not thick, LVOT peak pressure gradient 157mmHg. The most likely diagnosis is:

A. Coronary atherosclerotic heart disease
B. Mitral stenosis
C. Hypertrophic cardiomyopathy, non-obstructive type
D. Aortic stenosis
E. Hypertrophic cardiomyopathy, obstructive type

1. Male patient, 50 years old. Recurrent chest tightness, shortness of breath for 3 years. Rough systolic ejection murmur audible at left sternal border 3rd-4th intercostal spaces. Cardiac ultrasound: IVS thickness / LV posterior wall thickness = 1.7. Factors that do *not* worsen the patient's condition include:

A. Strenuous exercise
B. Use of positive inotropic drugs
C. Weightlifting
D. Taking beta-blockers
E. Hypertension

1. Female patient, 68 years old. Paroxysmal nocturnal dyspnea for over 1 month, breathlessness unable to lie flat for 2 days, no cough or sputum. History of old myocardial infarction. Physical examination: BP 130/90mmHg, HR 98 bpm. No neck vein distension, fine moist rales at lung bases, no lower limb edema. The most likely cause of breathlessness is:

A. Left heart failure
B. Right heart failure
C. Bronchial asthma
D. Myocarditis
E. Pneumonia

1. Male patient, 73 years old. Exertional chest tightness for over 6 years, dizziness for half a month. Physical examination: BP 155/73mmHg, HR 55 bpm. Mild cardiomegaly, S4 audible. Cardiac ultrasound: IVS thickness 16mm, LVOT pressure gradient 51mmHg. The unusable treatment for this patient is:

A. Digoxin
B. Surgical resection of hypertrophic septal myocardium
C. Diltiazem
D. Beta-blockers
E. Alcohol septal ablation

1. Male patient, 68 years old. Recurrent chest tightness and shortness of breath for 2 months, worsened with bilateral lower limb edema for 1 week. Cardiac ultrasound: LV end-diastolic diameter 58mm, generalized hypokinesis. Patient drank alcohol for 50 years, 500ml daily. The key to treatment lies in:

A. Early diagnosis, early anti-heart failure treatment
B. Early diagnosis, early myocardial protection therapy
C. Early diagnosis, immediate alcohol cessation
D. Early anti-heart failure treatment, gradual alcohol cessation
E. Early anti-heart failure treatment, myocardial protection

1. Male patient, 65 years old. Old extensive anterior wall myocardial infarction 7 years ago. Exertional chest tightness, palpitations, shortness of breath for 2 years, paroxysmal nocturnal dyspnea in the past week. Physical examination: Orthopnea, BP 160/90mmHg, P 120 bpm. P2 accentuated, no murmurs over valve areas. Fine moist rales at lung bases, scattered wheezes in both lungs. Soft abdomen, liver/spleen not palpable, no lower limb edema. Fasting blood glucose 4.2mmol/L. ECG: V1-V6 ST segment depression 0.05-0.1mV. Serum troponin normal. The most likely current diagnosis is:

A. Airway obstruction
B. Pulmonary embolism
C. Bronchial asthma
D. Acute myocardial infarction
E. Acute left heart failure

1. Male patient, 64 years old. Recurrent chest tightness with bilateral lower limb edema for 9 months. Physical examination: BP 99/60mmHg, HR 60 bpm. Pitting edema of lower limbs. ECG: Low voltage in limb leads. Cardiac ultrasound: All cardiac chambers enlarged except normal LV internal diameter, symmetric thickening of IVS and ventricular walls, specific strong ultrasonic echo spots under endocardial myocardium. The most likely diagnosis is:

A. Cardiac amyloidosis
B. Arrhythmogenic right ventricular cardiomyopathy
C. Constrictive pericarditis
D. Hypertrophic cardiomyopathy
E. Dilated cardiomyopathy

1. Male patient, 67 years old. Admitted due to fever for over 20 days. Cardiac ultrasound: Rupture of posterior mitral chordae tendineae causing posterior leaflet prolapse, moderate insufficiency, vegetation on posterior leaflet, blood culture suggests Streptococcus. The preferred anti-infective regimen is:

A. Gentamicin
B. Penicillin
C. Erythromycin
D. Streptomycin
E. Amikacin

1. Female patient, 56 years old. Admitted due to persistent palpitations for 5 days. Previously healthy. Physical examination: BP 142/80mmHg, normal heart size, HR 132 bpm, irregular rhythm. ECG: P waves absent, replaced by f waves, absolutely irregular ventricular rhythm. The preferred drug for rate control is:

A. Warfarin
B. Amiodarone
C. Propafenone
D. Adenosine
E. Bisoprolol

1. Male patient, 35 years old. Occasional atrial premature beats found on ECG during health checkup. Previously healthy. Physical examination: Normal heart size, HR 80 bpm, no murmurs over valve areas. The best management is:

A. IV lidocaine
B. Oral mexiletine
C. Oral propafenone
D. Find the cause, regular follow-up
E. Oral amiodarone

1. Female patient, 64 years old. Recurrent exertional shortness of breath for 10 years, worsened for over 1 year. Physical examination: Diastolic rumbling murmur at apex. Cardiac color ultrasound: Aortic and mitral valve echoes thickened, anterior mitral leaflet shows square-shaped pattern, posterior leaflet moves concordantly. The first considered diagnosis is:

A. Rheumatic heart disease, mitral stenosis
B. Left atrial myxoma
C. Hypertrophic obstructive cardiomyopathy
D. Rheumatic heart disease, aortic insufficiency
E. Congenital heart disease, ventricular septal defect

1. Female patient, 55 years old. Recurrent syncope for over 20 years. Preceded by abdominal pain with dizziness, nausea, vomiting, fainting occurs on the way to the bathroom or on the toilet, loss of consciousness during syncope, profuse sweating with incontinence, no significant chest tightness, pain, or palpitations, wakes up after a few minutes feeling tired, no significant sequelae. The most diagnostic test is:

A. Echocardiography
B. 24-hour Holter ECG
C. Head MRI
D. Tilt table test
E. Coronary angiography

1. Female patient, 19 years old. Fever for nearly 2 weeks, T around 38℃, with nausea, vomiting, diarrhea. Then developed palpitations, chest pain, dyspnea, syncope. Physical examination: Pale, lethargic. HR 40 bpm, regular rhythm, S1 diminished at apex, cannon a wave audible. Clinical diagnosis viral myocarditis. The most appropriate treatment is:

A. IV atropine
B. IV nitroglycerin drip
C. Subcutaneous epinephrine
D. Temporary cardiac pacemaker implantation
E. Cardioversion

1. Female patient, 15 years old. Fever for 5 days, chest pain for 2 days. Physical examination: Distended neck veins, weakened apical impulse, distant heart sounds, heart border enlarged to both sides, liver palpable 2cm below costal margin, pulsus paradoxus. ECG: ST segment elevation concave upward in leads I, aVL, V2-V6. The most likely diagnosis is:

A. Acute exudative pericarditis
B. Infective endocarditis
C. Constrictive pericarditis
D. Dilated cardiomyopathy
E. Acute heart failure

1. Female patient, 76 years old. Chest pain with shortness of breath for over 10 days. Echocardiography: Fluid dark area in pericardial cavity, 2cm behind posterior LV wall in diastole, 0.9cm anterior to RV wall, 2cm lateral to LV wall, 1.2cm at inferior pericardium. The absent sign is:

A. Absolute cardiac dullness borders enlarged to both sides
B. Pulse intensifies during inspiration
C. Dullness under left scapula, increased vocal fremitus
D. Dullness on percussion at right sternal border 3rd-6th intercostal spaces
E. Positive shifting dullness in abdomen

1. Male patient, 29 years old. Elevated BP for over 2 years, accompanied by paroxysmal headache, palpitations. Local hospital gave amlodipine, irbesartan, BP control fair. Our hospital: Norepinephrine 4911.40pg/ml (ref 19-121), Metanephrine 23.94pg/ml (ref 14-90). Whole abdomen enhanced CT: Hypervascular tumors at right renal hilum and left para-aortic area. For tumor resection, the preferred preoperative antihypertensive is:

A. Bisoprolol
B. Perindopril
C. Indapamide
D. Clonidine
E. Terazosin

1. Male patient, 43 years old. Admitted due to found hypokalemia for 6 years, dizziness for half a year. Hypertension history 13 years, usually poor BP control, recently taking amlodipine, valsartan, BP control fair. For this patient's diagnosis, the confirmatory test is:

A. Adrenal enhanced CT
B. Supine-standing test
C. Captopril suppression test
D. Dexamethasone suppression test
E. Water deprivation test

1. Male patient, 32 years old. Past history of hypertension for many years, self-reported irregular antihypertensive use, poor BP control. Tearing chest pain 1 hour ago without obvious cause, severe unbearable pain, our emergency BP 185/100mmHg, completed thoracic-abdominal aortic CTA: Aortic dissection, DeBakey type I. The BP management goal for this patient is:

A. Reduce SBP to 120mmHg within 30 minutes
B. Reduce SBP to 100mmHg within 30 minutes
C. Reduce SBP to 120mmHg within 2 hours
D. Reduce SBP to 100mmHg within 2 hours
E. To maintain peripheral circulation stability, cannot rapidly lower BP, BP should gradually normalize over 4-8 hours.

1. Male patient, 73 years old. Admitted due to left limb weakness for half a day. Emergency head MRI+DWI showed acute cerebral infarction near right lateral ventricle, emergency BP 188/90mmHg. The most suitable BP lowering plan is:

A. Close observation, temporarily no antihypertensive
B. IV labetalol
C. Sublingual nifedipine
D. IV esmolol
E. Oral captopril

1. Female patient, 63 years old. Recurrent substernal pain for 2 years without obvious cause, not severe, tolerable, lasts several minutes then relieves. Chest pain recurred 15 days ago, unbearable, given isosorbide mononitrate orally at local hospital with symptom relief. Now patient visits our outpatient for further examination. The examination that may have no positive result is:

A. Treadmill exercise test
B. Coronary CTA
C. Coronary angiography
D. Myocardial nuclear imaging
E. Cardiac ultrasound

1. Female patient, 77 years old. Substernal crushing pain on exertion 3 years ago, lasted hours without relief. Coronary angiography: Complete occlusion of LAD, 2 stents implanted. Occasional chest tightness on activity over 3 years, lasts 1 minute, relieves with rest. Chest pain with chest tightness recurred 2 days ago, severity increased. TnI: 0.01 µg/L. The first considered diagnosis is:

A. Stable angina
B. Unstable angina
C. Non-ST elevation myocardial infarction
D. Acute pericarditis
E. ST elevation myocardial infarction

1. Female patient, 42 years old. Chest pain 1 month ago, 2-3 times/day, lasts 4-5 minutes, relieves with rest. Frequency increased 3 days ago, 4-5 times/day, duration prolonged to 20-40 minutes. 76 minutes ago, patient at hospital. During blood draw, complained of anterior chest pain again, O2 sat 95%, TnT 0.27 µg/L. The inappropriate management measure is:

A. Revascularization
B. Cardiac monitoring
C. Analgesia and sedation
D. Oxygen therapy
E. Antiplatelet drugs

1. Male patient, 83 years old. Chest tightness after brisk walking about 200 meters over 2 months ago, relieved by rest. Chest tightness again on activity 3 days ago, area and severity increased, lasted 10 hours. 2 days ago, chest tightness again during recheck at hospital. Troponin 0.440 µg/L. The drug that does *not* reduce mortality with long-term use is:

A. Aspirin
B. Isosorbide mononitrate sustained-release
C. Atorvastatin
D. Metoprolol sustained-release
E. Perindopril

1. Male patient, 85 years old. Recurrent exertional chest tightness and shortness of breath for 1 week, relieved by rest. Precordial distending pain during night sleep 4 days ago, with chest tightness, relieved after hours. Then these symptoms recurred at rest. TnT 0.98. ECG see image below. Echocardiography no significant abnormality. BP 146/83mmHg, HR 90 bpm. Known GRACE score 149. The most suitable timing for PCI is:


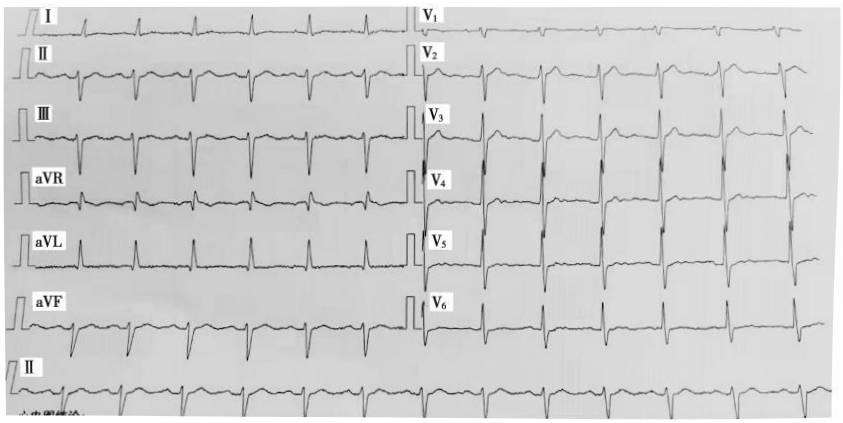


A. Within 2 hours
B. Within 24 hours
C. Within 72 hours
D. After 1 week
E. No need for interventional therapy

1. Male patient, 42 years old. Visited for recurrent dizziness, chest tightness for 3 months, bilateral lower limb edema for 2 months. Had syncope history. Physical examination: Enlarged heart border, HR 38 bpm. ECG suggests third-degree AV block. Cardiac ultrasound: LV enlargement, EF 60%. Myocardial enzymes normal range. The most appropriate management is:

A. IV isoproterenol drip
B. Atropine injection
C. IV hydrocortisone drip
D. Temporary pacemaker implantation
E. Permanent pacemaker implantation

1. Female patient, 68 years old. Has rheumatic heart disease, severe mitral stenosis, rapid ventricular rate atrial fibrillation, cardiac function class IV. When using digitalis, pay attention to contraindications. The disease that is a contraindication for digitalis is:

A. Congestive heart failure
B. Third-degree AV block
C. Atrial fibrillation
D. Supraventricular tachycardia
E. Atrial flutter

1. Male patient, 52 years old. Past history of WPW syndrome. Palpitations for 4 hours this time, BP 115/75mmHg. ECG: WPW syndrome with atrial fibrillation, ventricular rate 170 bpm. The usable treatment drug is:

A. Cedilanid
B. Verapamil
C. Diltiazem
D. Metoprolol
E. Procainamide

1. Female patient, 40 years old. Admitted due to recurrent chest tightness, palpitations for half a year, worsened for 3 days. ECG shows consecutive wide abnormal QRS complexes, visible ventricular capture. The most likely diagnosis is:

A. Paroxysmal supraventricular tachycardia
B. Atrial fibrillation with aberrancy
C. Atrial fibrillation with AV reentrant tachycardia
D. Sinus tachycardia
E. Ventricular tachycardia

1. Male patient, 45 years old. Admitted due to palpitations for over 4 years, recurrence for 3 months. Smoking, diabetes history. Outside hospital diagnosed paroxysmal ventricular tachycardia. The most common cause may be:

A. Heart failure
B. Coronary heart disease
C. Viral myocarditis
D. Atrial septal defect
E. Valvular heart disease

1. Male patient, 32 years old. Found elevated BP for 3 years. BP sustained at (170-200)/ (130-140) mmHg in the past year, headache, blurred vision in the past week. Fundoscopy found papilledema. The most likely diagnosis is:

A. Acute optic disc lesion
B. Cerebral hemorrhage
C. Malignant hypertension
D. Cerebral infarction
E. Hypertensive encephalopathy

1. Male patient, 16 years old. Nasal congestion with runny nose for 3 weeks, chest tightness, chest pain for 2 days. Physical examination: BP 116/60mmHg, HR 97 bpm. Myocardial enzymes elevated. ECG: Sinus tachycardia, ventricular premature beats. The first considered diagnosis is:

A. Sepsis
B. Acute myocarditis
C. Rheumatic heart disease
D. Acute pericarditis
E. Acute myocardial infarction

1. Male patient, 31 years old. Recurrent chest pain for 4 days. Had diarrhea 1 week ago. Dynamic ECG after admission: Sinus arrhythmia, total 9 atrial premature beats, 87 ventricular premature beats, R-R >2 seconds, total 2 times, longest 2.2 seconds. The suitable treatment measure after admission is:

A. Give prednisone
B. Implant temporary pacemaker
C. Give rest, supportive therapy
D. Implant permanent pacemaker
E. IV isoproterenol drip

**A3/A4 Type Questions**

(Questions 68-70 share the same case description)
Female patient, 40 years old. Palpitations, shortness of breath on exertion for 5 years, unable to lie flat at night for 2 weeks, past history of recurrent joint pain. Physical examination: Malar flush, diastolic murmur audible at apex.

1. The most helpful test for confirmation is:

A. Blood culture + sensitivity
B. Blood routine
C. Chest X-ray
D. Echocardiography
E. MRI

1. The most likely diagnosis is:

A. Aortic stenosis
B. Aortic insufficiency
C. Mitral stenosis
D. Mitral insufficiency
E. Tricuspid stenosis

1. The preferred treatment method is:

A. Medication
B. Mitral commissurotomy
C. Artificial valve replacement
D. Percutaneous balloon mitral valvuloplasty
E. Prevent embolism

(Questions 71-73 share the same case description)
Female patient, 59 years old. 5 years ago, began having precordial chest tightness and shortness of breath on exertion, relieved by rest, occasional dull chest pain, no dizziness, headache, cough, sputum, chills, fever, abdominal pain, distension. Patient did not pay attention or treat. Symptoms recurred over 5 years, nature and severity same. Coronary CTA 1 month ago: Right and left coronary arteries both originate above left sinus, considered congenital developmental abnormality; proximal RCA calcified plaque, mild lumen stenosis; distal left main mixed plaque with moderate-severe lumen stenosis, suggest DSA; LAD atherosclerotic plaque, mild lumen stenosis; mid LAD local myocardial bridge; proximal LCx localized calcified plaque with moderate lumen stenosis.

1. The first considered current diagnosis is:

A. Acute ST-elevation myocardial infarction
B. Unstable angina
C. Stable angina
D. Acute non-ST-elevation myocardial infarction
E. Silent coronary heart disease

1. The preferred treatment during chest tightness episodes is:

A. Nifedipine
B. Sodium nitroprusside
C. Verapamil
D. Warfarin
E. Nitroglycerin

1. The drug that cannot improve this patient's prognosis is:

A. Aspirin
B. Atorvastatin
C. Isosorbide mononitrate
D. Metoprolol sustained release
E. Perindopril

(Questions 74-75 share the same case description)
Female patient, 65 years old. Admitted due to paroxysmal chest tightness for 8 years, persistent chest pain for 8 hours. Admission BP 150/90mmHg, diagnosed acute anterior wall MI.

1. The ECG change supporting diagnosis is:

A. Abnormal Q waves in II, III, aVF with ST segment elevation convex upward
B. Abnormal Q waves in V1-V4 with ST segment elevation convex upward
C. Pulmonary P waves in I, aVL
D. Frequent ventricular premature beats
E. Widespread ST segment depression

1. On hospital day 2, patient developed chest tightness, profuse sweating, pale. Physical examination: HR 126 bpm, regular, dry/wet rales in lungs, BP 80/50mmHg, considered combined cardiogenic shock. At this time, it is unsuitable to use:

A. Intra-aortic balloon pump
B. IV furosemide
C. IV dopamine drip
D. IV propranolol
E. Sodium nitroprusside

(Questions 76-78 share the same case description)
Male patient, 60 years old. Precordial pain on exertion in the past month, dyspnea, with a sense of suffocation, pain unrelated to deep breathing.

1. The most likely cause is:

A. Chronic bronchitis with emphysema
B. Pleurisy
C. Pericardial effusion
D. Angina pectoris
E. Pulmonary tuberculosis

1. The test most likely to show abnormality at this time is:

A. Blood routine
B. ECG
C. Chest X-ray
D. Serum myocardial enzymes
E. Chest ultrasound

1. Based on the clinical presentation, many diseases need differentiation. The main distinguishing point between angina and acute MI is:

A. Pain location
B. Pain nature
C. Pain severity
D. Pain radiation site
E. Pain duration

(Questions 79-80 share the same case description)
Male patient, 60 years old. Recurrent palpitations, chest pain for 7 years. Diagnosed coronary heart disease, angina. Pain worsened last night, nitroglycerin ineffective, emergency admission. ECG showed acute MI.

1. The most common arrhythmia in this patient is:

A. Paroxysmal atrial fibrillation
B. Atrial premature beats
C. Junctional premature beats
D. Frequent ventricular premature beats
E. AV block

1. The preferred drug for ventricular arrhythmias in this patient is:

A. Amiodarone
B. Propranolol
C. Nifedipine
D. Verapamil
E. Digitalis

(Questions 81-85 share the same case description)
Female patient, 35 years old. Paroxysmal palpitations for over 10 years, each episode sudden, lasting half hour to 3 hours. This episode lasted half hour before visit. Examination: BP 90/60mmHg, HR 200 bpm, regular rhythm, no murmur, lungs (-).

1. The most likely arrhythmia this time is:

A. 2:1 atrial flutter
B. Paroxysmal ventricular tachycardia
C. Paroxysmal supraventricular tachycardia
D. Sinus tachycardia
E. Atrial fibrillation

1. The best treatment measure is:

A. IV adenosine
B. IV phenytoin
C. IV potassium chloride drip
D. IV lidocaine
E. IV propranolol

1. If ECG shows QRS width 0.14-0.16 seconds, slurred onset, RR intervals absolutely irregular. The most likely arrhythmia now is:

A. Paroxysmal ventricular tachycardia
B. WPW syndrome with atrial fibrillation
C. Atrial fibrillation with aberrancy
D. Atrial fibrillation with bundle branch block
E. Supraventricular tachycardia with aberrancy

1. If BP drops to 82/50mmHg, the best treatment plan is:

A. Electrical cardioversion
B. IV potassium chloride drip
C. IV lidocaine
D. IV cedilanid
E. IV verapamil

1. The contraindication for this type of arrhythmia is:

A. Propafenone
B. Electrical cardioversion
C. Quinidine
D. Digitalis preparations
E. Amiodarone

(Questions 86-88 share the same case description)
Male patient, 32 years old. Recurrent paroxysmal palpitations for 10 years. ECG during episodes diagnosed tachycardia. HR 188 bpm, symptoms quickly relieved after IV verapamil. Palpitations again today for half hour, with fatigue, urinary frequency. ECG: HR 180 bpm, regular rhythm, QRS morphology and duration normal, no significant ST-T change.

1. The most likely diagnosis is:

A. Sinus tachycardia
B. Atrial tachycardia
C. Ventricular tachycardia
D. Supraventricular tachycardia
E. Atrial flutter

1. The most meaningful examination is:

A. ECG
B. Electrophysiological study
C. Dynamic ECG
D. Cardiac ultrasound
E. Atropine test

1. The best treatment method is:

A. Intracoronary stent therapy
B. Radiofrequency ablation
C. Chemical ablation
D. Oral medication maintenance
E. No long-term medication, give IV verapamil etc. temporarily during symptomatic episodes

(Questions 89-91 share the same case description)
Female patient, 24 years old. Palpitations, chest tightness, fatigue, general joint pain for 2 weeks, chest pain after activity. Physical examination: HR 80 bpm, lungs normal, full heart border, regular rhythm, diminished S1 at apex, audible S3. Liver/spleen not enlarged. Upper respiratory infection 1 month ago. ECG: PR interval 0.24-0.28 seconds.

1. The most likely diagnosis is:

A. Rheumatic myocarditis
B. Viral myocarditis
C. Infective endocarditis
D. Cardiomyopathy
E. Coronary heart disease

1. The unsuitable treatment measure is:

A. IV myocardial nutritional agents
B. Oral ACEI
C. IV vitamin C
D. Oral digoxin
E. Quiet bed rest

1. Patient suddenly dizzy, then loss of consciousness, immediate BP 70/40mmHg, HR 36 bpm, regular rhythm. The most likely ECG finding now is:

A. Third-degree AV block
B. Ventricular tachycardia
C. Sinus bradycardia
D. Atrial fibrillation
E. Ventricular fibrillation

(Questions 92-94 share the same case description)
Male patient, 26 years old. Fever for 1 week, with muscle pain and chest pain. Pericardial friction rub audible. ECG: ST elevation in II, III, aVF, aVL, V2-V6.

1. The most likely diagnosis is:

A. Angina pectoris
B. Dilated cardiomyopathy
C. Acute pericarditis
D. Myocarditis
E. Infective endocarditis

1. The most common pathogen is:

A. Adenovirus
B. Coxsackie B virus
C. Herpes simplex virus
D. RS virus
E. Mycoplasma

1. If pericardial effusion develops, the most prominent symptom is:

A. Precordial pain
B. Fever
C. Dyspnea
D. Hoarseness
E. Dysphagia

(Questions 95-97 share the same case description)
Female patient, 38 years old. Progressive palpitations, shortness of breath with precordial pain for half a month. Afternoon low-grade fever, T 37.2-38.5℃, night sweats. Pericardial friction rub audible.

1. To confirm diagnosis, the first test is:

A. Cardiac ultrasound
B. 24-hour Holter ECG
C. Chest PA & lateral X-ray
D. Lung CT
E. MRI

1. The most likely diagnosis is:

A. Pneumonia
B. Pulmonary embolism
C. Angina pectoris
D. Myocardial infarction
E. Pericardial effusion (tuberculous)

1. If dyspnea worsens, BP 86/70 mmHg, palpable pulsus paradoxus, distant muffled heart sounds. The emergency measure should be:

A. Cardiopulmonary resuscitation
B. High-dose diuretics
C. Nitroglycerin
D. Emergency PCI
E. Pericardiocentesis

(Questions 98-100 share the same case description)
Male patient, 46 years old. Hypertension over 10 years, irregular nifedipine, propranolol, specific dose unknown. Usual BP (130-180)/ (90-110) mmHg, highest 190/120 mmHg. Physical examination: HR 100 bpm, BP 170/100 mmHg. Cardiac dullness enlarged to left lower, grade 2/6 blowing murmur at apex, A2>P2. Liver/spleen not palpable. No kidney area percussion pain. Auxiliary tests: Blood WBC 9.3×10⁹/L, N 65%, Hb 126g/L. ECG: Sinus rhythm 110 bpm, axis -25°, RI+RIII=3.2 mV, RV5=3.0 mV, V3-V6 ST segment horizontal depression 0.2mV with low T waves.

1. The patient's diagnosis is:

A. Hypertension grade 3, very high risk
B. Hypertension grade 3, high risk
C. Hypertension grade 2, high risk
D. Hypertension grade 2, very high risk
E. Dilated cardiomyopathy

1. The preferred treatment drugs do *not* include:

A. Nifedipine
B. Metoprolol
C. Captopril
D. Hydrochlorothiazide
E. Nitroglycerin

1. For hypertensive patients taking antihypertensive drugs, the BP goal is:

A. BP reduced to normal range, i.e., younger <120-130/80mmHg, elderly can be reduced to <140/90 mmHg
B. Reduced to borderline level, i.e., (140-160)/ (90-95) mmHg
C. SBP controlled at 160mmHg, DBP at 85 mmHg
D. BP controlled to patient's tolerable level
E. BP controlled at high end of normal low, i.e., (90-100)/ (60-70) mmHg

**Professional Practical Skills**

**【Case 1】**
Male patient, 48 years old. Palpitations started 4 years ago, occurring 4-5 times per year, feeling palpitations and chest tightness during episodes, lasting about 30 minutes and terminating spontaneously, sometimes lasting 3-6 hours. Now visited due to chest tightness and palpitations.
Physical examination: BP 95/60mmHg, varying intensity of S1, irregular heart rhythm, HR 116 bpm, irregular pulse, 102 bpm. Liver enlarged, pitting edema of both lower limbs.

**Question 1:** The diagnosis of his arrhythmia is:
A. Angina pectoris
B. Ventricular extrasystoles
C. Third-degree atrioventricular block
D. Supraventricular tachycardia
E. Paroxysmal atrial fibrillation
F. Frequent atrial premature beats

**[Hint]** Cardiac color ultrasound results: Diastolic left atrial diameter: 46mm, Diastolic left ventricular diameter: 65mm, Diastolic right atrial diameter: 39mm, Diastolic right ventricular diameter: 23mm, and indicated: Generalized hypokinesis of ventricular walls, mild mitral regurgitation, EF 0.34.

**Question 2:** The etiological diagnosis considered is:
A. Coronary heart disease
B. Rheumatic heart disease
C. Dilated cardiomyopathy
D. Hypertrophic cardiomyopathy
E. Pericardial effusion
F. Valvular heart disease

**Question 3:** Appropriate viewpoints regarding arrhythmia treatment are:
A. Alleviate and eliminate symptoms of arrhythmia
B. Correct hemodynamic disorders caused by arrhythmia
C. Terminate life-threatening arrhythmias
D. Prevent complications
E. Prevent arrhythmia recurrence
F. All arrhythmias can potentially threaten life and must be treated

**Question 4:** Necessary treatment measures applied to the patient include:
A. Digitalis drugs
B. Intravenous verapamil
C. Intravenous aminophylline
D. Intravenous furosemide push
E. Intravenous propafenone push
F. Beta-blockers
G. ACEI
H. Antiplatelet therapy
I. Myocardial metabolism improving drugs
J. Implant anti-arrhythmia pacemaker
K. Dopamine injection

**【Case 2】**
Male patient, 76 years old. Past history: Hypertension for over 20 years, diabetes for 8 years, good BP and glucose control in the past year, no angina attacks. Underwent gastric cancer surgery 1 week ago, received IV nutrition post-op, shortness of breath started 2 days ago, waking up at night short of breath, sudden dyspnea and orthopnea today during infusion.
Physical examination: BP 180/110mmHg, HR 132 bpm. Apathetic, cyanotic lips, widespread dry and wet rales in both lungs, varying heart sound intensity, irregular rhythm. Mild lower limb edema. ECG shows rapid atrial fibrillation, myocardial ischemia. Blood glucose 7.2mmol/L, troponin normal, D-dimer normal.

**Question 1:** The patient's current main diagnosis is:
A. Acute myocardial infarction
B. Acute right heart failure
C. Pulmonary infection
D. Acute left heart failure
E. Diabetic hyperosmolar coma
F. Hypertension grade 3, very high-risk group
G. Hypertension grade 3, high-risk group

**Question 2:** The cause of dyspnea is:
A. Impaired gas exchange due to pulmonary infection
B. Acute left heart failure due to excessive/rapid IV fluid
C. Worsening coronary heart disease
D. Pulmonary infarction due to prolonged bed rest post-op
E. Central nervous system affectation from diabetic hyperosmolar coma
F. Diabetic nerve damage

**Question 3:** Appropriate treatments include:
A. Control infusion volume and speed
B. IV diltiazem drip for BP lowering
C. Change to higher-level antibiotics
D. IV diuretics
E. Oral hydrochlorothiazide diuresis
F. IV beta-blockers for heart rate control
G. IV sodium nitroprusside drip
H. IV cedilanid injection

**Question 4:** After the patient's condition stabilizes, drugs that can be used to control ventricular rate in atrial fibrillation include:
A. Dihydropyridine calcium channel blockers
B. Non-dihydropyridine calcium channel blockers
C. Beta-blockers
D. Digitalis
E. Amiodarone
F. Sotalol

**Question 5:** Drugs for cardioversion in AF patients include:
A. Flecainide
B. Digoxin
C. Propafenone
D. Amiodarone
E. Dofetilide
F. Sotalol

**【Case 3】**
Male patient, 66 years old. Claims being told about a possible heart problem during a physical exam over 50 years ago, specific diagnosis unknown. Started having exertional chest tightness and shortness of breath over 10 years ago, significantly decreased physical capacity, never received specific treatment. BP found up to 150/90mmHg during exams, usual monitoring BP around 120/80mmHg, no formal antihypertensive treatment. No past chronic respiratory disease. Precipitated this time by catching a cold due to weather change, then developed cough, sputum (white sticky), no fever, no antibiotics used; also developed nocturnal chest tightness, shortness of breath, and dyspnea when lying flat, slightly relieved by sitting up, not clearly related to physical activity; then developed bilateral symmetric pitting lower limb edema, with decreased urine output, no facial or sacral edema, significantly decreased physical capacity, didn't seek medical attention. Worsening symptoms led to visit.
Physical examination: T 36.3℃, R 24 bpm, P 68 bpm, BP 185/95mmHg. Conscious, chronic mitral facies, automatic posture, no lip cyanosis, trachea midline, no skin/sclera jaundice, no enlarged superficial lymph nodes, distended neck veins. Symmetric chest, coarse breath sounds bilaterally, right lower lung breath sounds slightly weaker than contralateral, fine moist rales audible in left lower lung. Globally enlarged heart, strong heart sounds, HR 76 bpm, irregular rhythm, atrial fibrillation rhythm, biphasic murmur audible at apex, mainly systolic. Soft abdomen, liver 3-4 fingerbreadths below costal margin, no obvious tenderness, shifting dullness negative. Bilateral pitting edema of lower limbs.

**Question 1:** The most likely precipitating factor for this exacerbation is:
A. Respiratory tract infection
B. Pulmonary embolism
C. Arrhythmia
D. Myocardial infarction
E. Increased blood volume
F. Rheumatic activity

**Question 2:** The treatment this patient should receive is:
A. Anti-infective therapy
B. Anti-heart failure therapy
C. Anticoagulation therapy
D. Thrombolytic therapy
E. Antihypertensive therapy
F. Supportive symptomatic treatment (e.g., correct hypoalbuminemia, hypokalemia)

**Question 3:** Appropriate anti-heart failure treatments for this patient include:
A. Diuresis: Furosemide
B. Vasodilation: Nitroglycerin
C. Neuroendocrine antagonism: Spironolactone
D. Inotropy: Digitalis
E. Oxygen therapy
F. Control water/sodium intake, bed rest

**【Case 4】**
Male patient, 70 years old. Sudden crushing substernal pain for 4 hours, radiating to left shoulder, accompanied by profuse sweating and fear.

**Question 1:** The most valuable examination for initial diagnosis is:
A. Chest X-ray
B. Chest CT
C. Electrocardiogram
D. Echocardiography
E. Radionuclide imaging
F. Cardiac MRI

**Question 2:** Lab tests show CK-MB over 2 times normal. The most likely diagnosis is:
A. Angina pectoris
B. Acute myocardial infarction
C. Aortic dissection
D. Acute heart failure
E. Sinus arrest
F. Acute pulmonary embolism

**Question 3:** Causes that can lead to this disease include:
A. Intracoronary thrombosis
B. Shock
C. Dehydration
D. Pregnancy
E. Severe arrhythmia
F. Infection

**Question 4:** Complications of this disease include:
A. Cardiac rupture
B. Infective endocarditis
C. Aortic dissection
D. Papillary muscle dysfunction
E. Embolism
F. Cardiac neurosis

**Question 5:** Drugs that can be used in the acute phase of this disease treatment (if no contraindications) are:
A. Heparin
B. Aspirin
C. Beta-blockers
D. Calcium channel blockers
E. Simvastatin
F. Angiotensin-converting enzyme inhibitors

**【Case 5】**
Female patient, 74 years old. Found high BP 8 years ago, highest BP 174/110mmHg, usually did not take medication regularly, BP usually (140-150)/(90-100)mmHg. No stroke, hemiplegia, no exertional shortness of breath or fatigue, no nocturnal orthopnea, etc. 7 days ago, developed precordial pain while going downstairs, persisted after rest, relieved slightly after about 20 minutes. No nausea, vomiting, syncope. 2 days ago, chest pain recurred after bowel movement, accompanied by dyspnea, palpitations, BP unmeasurable at local hospital, considered acute right ventricular MI, given BP raising treatment (details unknown), poor effect, still had dyspnea, palpitations, and developed abdominal distension, poor appetite, ankle edema, came to our hospital for further diagnosis. Since onset, poor spirit, appetite, decreased physical capacity. No past diabetes or hyperlipidemia denies smoking history, drug allergies, or similar family history.

**Question 1:** The patient's possible diagnosis is:
A. Hypertension complicated by heart failure
B. Acute coronary syndrome
C. Acute right ventricular myocardial infarction
D. Right heart failure
E. Acute pulmonary embolism
F. Acute infection-induced heart failure

**[Hint]** T 36.6℃, R 26 bpm, P 116 bpm, BP 82/54mmHg. Conscious, mild lip cyanosis, trachea midline, no skin/sclera jaundice, no enlarged superficial lymph nodes, distended neck veins. Tachypneic, symmetric chest, coarse breath sounds bilaterally, no significant dry/wet rales in lungs. Normal heart size, strong heart sounds, HR 116 bpm, regular rhythm, grade 2/6 systolic murmur audible at tricuspid area. Soft abdomen, no obvious tenderness/rebound tenderness, liver/spleen not palpable below costal margin, hepatojugular reflux sign (+), shifting dullness negative, bilateral pitting edema of lower limbs, prominent bilateral lower limb varicose veins.

**Question 2:** The possible cause of this patient's heart failure is:
A. Acute right ventricular myocardial infarction
B. Hypertrophic cardiomyopathy
C. Acute pulmonary embolism
D. Hypertension
E. Valvular heart disease with insufficiency
F. Dilated cardiomyopathy

**[Hint]** ① Blood routine: WBC 11.2×10⁹/L, N 79.26%, RBC 4.3×10¹²/L, Hb 116g/L, PLT 183×10⁹/L. ② Urinalysis: Specific gravity >1.03, protein (+), rest negative. ③ Liver function: AST 57 U/L↑, ALT 48 U/L↑, rest normal. ④ Renal function/electrolytes: Urea nitrogen 6.19 mmol/L, Creatinine 74.5 µmol/L, Uric acid 405.1 µmol/L, K⁺ 4.2 mmol/L, Na⁺ 142 mmol/L. ⑤ Fasting glucose 5.39 mmol/L, Total cholesterol 3.74 mmol/L, Triglycerides 1.50 mmol/L, HDL-C 1.10 mmol/L, LDL-C 1.83 mmol/L. ⑥ TnI 0.882 ng/ml↑, D-dimer 1.6 mg/L↑. ⑦ NT-pro BNP 11884 pg/ml↑. ⑧ Coagulation: PT 14.5 sec, INR 1.16, APTT 37.5 sec. ⑨ Arterial blood gas: pH 7.464, PaO₂ 71.6 mmHg, PaCO₂ 30.3 mmHg, SO₂ 95.9%. ⑩ Cardiac ultrasound: Right ventricular enlargement, severe tricuspid regurgitation, mild pulmonary hypertension. Small pericardial effusion. ⑪ ECG: Suggests sinus tachycardia, occasional ventricular premature beats, S₁Q₃T₃ pattern, T wave inversion or flattening in V₁-V₄, T wave inversion in II, III, aVF, Q waves in V₃R-V₅R. ⑫ Coronary CTA & Pulmonary CTA: Coronary atherosclerosis plaques visible, no significant stenosis. Bilateral basal trunk and multiple segmental pulmonary emboli, complete occlusion of right middle lobe artery and parts of bilateral lower lobe pulmonary arteries; pulmonary artery dilated, right heart shadow enlarged, reflux contrast in azygos and vena cava suggesting right heart dysfunction; bilateral pleural and small pericardial effusion. Carotid and bilateral lower limb arterial/venous color Doppler: Carotid atherosclerosis plaques visible, no significant stenosis. Thrombosis in right posterior tibial and popliteal veins, scattered small atherosclerosis plaques in bilateral lower limb arteries, blood flow patent.

**Question 3:** The patient's diagnoses include:
A. Acute pulmonary embolism
B. Acute right heart failure
C. Lower limb deep vein thrombosis
D. Hypertension grade 3, very high-risk group
E. Coronary atherosclerosis
F. Peripheral arterial atherosclerosis

**Question 4:** Treatment measures to be taken include:
A. Oxygen therapy
B. Thrombolytic therapy
C. Volume expansion
D. Raise blood pressure
E. Lower pulmonary artery pressure
F. Bronchoscopic embolectomy

**【Case 6】**
Male patient, 54 years old. Chest tightness, exertional chest pain for 4 years. Suspected hypertrophic obstructive cardiomyopathy, came for consultation.

**Question 1:** What helps differentiate old myocardial infarction from hypertrophic obstructive cardiomyopathy is:
A. Exertional angina
B. Precordial murmur can be influenced by drugs and maneuvers
C. Left ventricular hypertrophy
D. Pathological Q waves on ECG
E. Positive late potentials
F. Decreased EF on echocardiography

**Question 2:** When diagnosing obstructive hypertrophic cardiomyopathy, the disease that does *not* need to be differentiated by cardiac auscultation is:
A. Old myocardial infarction
B. Ventricular septal defect
C. Congenital aortic stenosis
D. Rheumatic mitral insufficiency
E. Aortic insufficiency
F. Rheumatic aortic stenosis

**Question 3:** Incorrect descriptions regarding factors affecting outflow tract obstruction in hypertrophic obstructive cardiomyopathy are:
A. Obstruction decreases when peripheral resistance drops
B. Obstruction worsens when myocardial contractility strengthens
C. Obstruction worsens when pressure gradient between LV cavity and LVOT increases
D. Obstruction worsens when anterior mitral leaflet anterior motion is significant
E. Obstruction lessens when systolic volume increases
F. Obstruction worsens after starting (exercise?)

**Question 4:** To intensify the systolic murmur at the left lower sternal border in hypertrophic obstructive cardiomyopathy, one should choose:
A. IV norepinephrine drip
B. Propranolol injection
C. IV isoproterenol drip
D. Squatting position
E. Sublingual nitroglycerin
F. Oral metoprolol

**Question 5:** Correct statements about the treatment of hypertrophic cardiomyopathy are:
A. Enhance myocardial contractility
B. Mainly myocardial relaxation
C. Maintain sinus rhythm
D. Reduce cardiac preload
E. Severe obstructive patients may undergo interventional therapy
F. Mainly vigorous diuresis

**【Case 7】**
Female patient, 23 years old, accountant. Blurred vision for nearly 15 days, occasional dizziness, vomiting. Blurred vision worsened 5 days ago, with persistent dizziness, vomiting, unrelieved. No syncope, blackouts, no fever, cough, sputum, no chest tightness, pain, dyspnea. Local visit found BP 220/130mmHg, given Beijing降压片0号 (hypotensive agent) 1 tablet, 3 times/day, transferred due to worsening symptoms. Since onset, normal spirit, diet, sleep, bowel/bladder, no significant change in physical capacity or weight. Denies diabetes, hypertension history, denies TB, hepatitis. No gross hematuria, no urinary frequency, urgency, dysuria. Denies fever, joint pain. Normal menstrual flow. No family hypertension history.
Physical examination: T 36.5℃, P 110 bpm, R 22 bpm, left arm BP 210/120mmHg, Right arm BP 200/105mmHg, Left leg BP 230/130mmHg, Right leg BP 220/120mmHg. Conscious, symmetric facial muscles, tongue midline, steady breathing, no lip cyanosis, trachea midline, no thyroid enlargement. Clear lung sounds bilaterally, no dry/wet rales. HR 110 bpm, regular, no murmurs. Soft abdomen, liver/spleen not palpable below costal margin, bilateral kidney area percussion reveals sour distending sensation, no abdominal vascular bruits. No lower limb edema, neurological exam normal. Fundoscopy: Optic disc borders clear, perivascular patchy hemorrhages and cotton-wool spots near disc, hypertensive retinopathy stage III.

**Question 1:** Correct descriptions about this patient's condition are:
A. Cerebrovascular disease can be temporarily ruled out
B. Cardiac damage from hypertension can be temporarily ruled out
C. Need to further clarify target organ function damage
D. Renal artery stenosis can be temporarily ruled out
E. This patient is likely to have malignant hypertension
F. Should be alert for hypertensive emergency

**[Hint]** ① Urinalysis: Urine protein (+++), Occult blood (+++), Urine WBC (++); Sediment quant: Urine RBC 424.8/µl, Urine WBC 36.2/µl, Urine epithelial cells 34.0/µl, Urine casts 2.89/µl. ② Blood routine: RBC 2.64 T/L, Hb 82 g/L, HCT 23.5%, N 74.5%, Eosinophils 0.4%, PLT 12×10⁹/L. ③ Liver/renal function electrolytes: Direct bilirubin 1.4 µmol/L, GGT 5 U/L, Total protein 57.7 g/L, Albumin 33.0 g/L, A/G ratio 1.3; BUN 18.86 mmol/L, Cr 456.4 µmol/L, Uric acid 432.9 µmol/L; Sodium 135.0 mmol/L, Potassium 4.7 mmol/L, Magnesium 1.01 mmol/L. ④ ENA(-), Thyroid function trio (-); Cortisol 8am 128 µg/L, 4pm 51 µg/L, 0am 194 µg/L; CA 3.45 nmol/L; RAS: PRA 7474 pmol/(L·h), Ang II 138 pmol/L, ALD 213 pmol/L; Anti-GPCR antibodies: Anti-α1 receptor Ab 1:80, Anti-AT1 receptor Ab 1:40. ⑤ ECG: Sinus tachycardia. ⑥ CT plain scan: Brain no significant abnormality. Bilateral adrenals normal. ⑦ Ultrasound: Poor demarcation of renal cortex/medulla and collecting system bilaterally, nephropathy pattern. High renal artery resistance index bilaterally, decreased renal blood flow signal.

**Question 2:** The patient's diagnoses include:
A. Malignant hypertension
B. Stage III fundus changes
C. Renal impairment (azotemia stage)
D. Primary hypertension with acute kidney injury
E. Glomerulonephritis complicated by malignant hypertension
F. Secondary hypertension related to adrenals

**[Hint]** Renal biopsy and pathology: Glomerular ischemia with segmental endothelial lesions, severe. Tubulointerstitial lesions; Arterial hyalinization, luminal stenosis/occlusion, and arterial mucoid edema, necrosis, and onion-skin lesions. Immunofluorescence: 2 enlarged glomeruli: IgM (+ ~ 4+), C3 (+ ~ 4+), C1q (+ ~ 4+), C4 (+ ~ 3+) granular deposits in segmental mesangial and some capillary loop areas; IgG (-) and IgA (-). Electron microscopy: Irregular glomerular capillary loops, no endothelial hyperplasia, widened subendothelial space in parts, irregularly thickened basement membrane in segments, widespread foot process effacement, no electron-dense deposits in mesangial/paramesangial areas.

**Question 3:** The underlying cause of this patient's malignant hypertension can be judged as:
A. Primary hypertension
B. Glomerulonephritis
C. Nephrotic syndrome
D. Chronic pyelonephritis
E. Diabetic nephropathy
F. Renal amyloidosis

**【Case 8】**
Male patient, 43 years old. Admitted due to sudden chest/back pain during activity for 2 hours, syncope once. Hypertension and diabetes history 8 years, no chronic cough or diabetes history. Physical examination: Acute ill appearance, BP 195/105mmHg, R 28 bpm, HR 45 bpm. Irregular rhythm, P2 accentuated, diastolic rumbling murmur audible at left sternal border 3rd intercostal space. ECG: Complete right bundle branch block, ST segment horizontal depression 0.2mV in II, III, aVF.

**Question 1:** The preliminary diagnosis may be:
A. Acute massive pulmonary embolism
B. Rheumatic heart disease, aortic insufficiency with acute left heart failure
C. Severe myocarditis
D. Acute inferior wall non-ST elevation myocardial infarction
E. Aortic dissection involving right coronary ostium
F. Tension pneumothorax

**Question 2:** If chest X-ray suggests widened mediastinum, and echocardiography suggests significantly enhanced aortic intima echo with displacement into the aortic lumen, the core content for further emergency examination is:
A. Pulmonary artery CTA
B. Pulmonary vein CTA
C. Coronary CTA
D. Aortic CTA
E. Cardiac MRI
F. Cardiac ECT

**Question 3:** Besides hypertension, the causes of this type of disease also include:
A. Cystic medial necrosis of aorta
B. Congenital cardiovascular disease
C. Atherosclerosis
D. Takayasu's arteritis
E. Cardiac interventional procedures
F. Trauma

**Question 4:** The general treatment principles for this type of disease include:
A. Absolute bed rest
B. Analgesia and sedation
C. Blood pressure control
D. Heart rate reduction
E. Heart failure control
F. Surgical evaluation for indication and timing

**Question 5:** Incorrect internal medicine drug use in the acute phase for this patient is:
A. Morphine
B. Low molecular weight heparin
C. Sodium nitroprusside
D. Metoprolol
E. Aspirin + Clopidogrel
F. Ticagrelor

**【Case 9】**
Male patient, 60 years old. History of extensive anterior wall MI 5 years ago, no interventional therapy, long-term aspirin and atorvastatin. Admitted this time due to chest pain, palpitations for 2 days. During hospitalization, patient suddenly fell to the ground while going to the bathroom in the morning, unresponsive, regained consciousness after 30 seconds, with chest tightness, unable to describe the episode.

**Question 1:** The cause of consciousness disturbance most likely related is:
A. Third-degree AV block
B. Stroke
C. Malignant arrhythmia
D. Ventricular aneurysm rupture
E. Epilepsy
F. Orthostatic hypotension

**Question 2:** If you happen to pass by the ward and witness the patient's loss of consciousness, the incorrect primary management is:
A. Check cardiac monitor for malignant arrhythmia
B. Prepare defibrillator for cardioversion
C. Perform mouth-to-mouth artificial respiration
D. Start chest compressions at 80/min
E. IV push 1mg epinephrine
F. IV push 150mg amiodarone

**Question 3:** After stabilization, tests helpful for definitive diagnosis are:
A. Dynamic ECG
B. Dynamic BP
C. Echocardiography
D. Intracardiac electrophysiology study
E. Coronary angiography
F. Cardiac MRI

**Question 4:** If considering post-MI scar-related ventricular tachycardia as the cause of syncope, treatments that can be considered are:
A. Long-term oral bisoprolol
B. Long-term oral amiodarone
C. ICD implantation
D. CRT-P implantation
E. Radiofrequency ablation
F. Long-term oral propafenone

**Question 5:** If considering acute pulmonary embolism as the cause of syncope, correct descriptions are:
A. May have had a large area embolism
B. The triad of chest pain, hemoptysis, dyspnea is more common clinically
C. ECG may show S wave in I, Q wave + T wave inversion in III
D. Pulmonary vein CTA is the gold standard for diagnosis
E. Add warfarin, use LMWH until INR reaches target
F. Add warfarin, avoid simultaneous LMWH to reduce bleeding risk

**【Case 10】**
Male patient, 45 years old. Recurrent cough, sputum for over 20 years, worse after colds, sometimes with mild wheezing when severe, no chronic low-grade fever, fatigue, night sweats, chest pain, hemoptysis, palpitations. No formal diagnosis/treatment, only antibiotics, antitussives/expectorants when severe, short-term treatment provided relief. Cough/wheeze recurred/worsened 2 months ago after cold, sputum gray-white mucoid, copious, with significant exertional dyspnea, local anti-infection (details unknown), antitussive/expectorant (e.g., acetylcysteine, 咳特灵) ineffective, breathlessness gradually worsened, developed nocturnal orthopnea. No recent fever/chills, palpitations, chest pain, hemoptysis, syncope, abdominal distension/loss of appetite, oliguria, or lower limb edema. Outside hospital chest X-ray: Markedly enhanced lung markings bilaterally, enlarged heart, water-bottle shaped cardiac shadow. Recent poor spirit/sleep, fair diet, normal bowel/bladder, significantly decreased physical capacity, stable weight. No family genetic history. No TB, hepatitis. No food/drug allergies. Smoker 30 years, 10 cigarettes/day; Drinker 30 years, 250ml/day. Long-term dust exposure job (stone processing). Diagnosed with chronic bronchitis outside hospital for chronic cough/wheeze. Denies congenital heart, digestive, endocrine, hematologic, neurologic diseases.
Physical examination: T 36.8℃, P 86 bpm, R 22 bpm, BP 110/88mmHg. Normal development, slightly poor nutrition, conscious, chronic ill appearance, walked into ward assisted, automatic posture, cooperative. No skin jaundice/spider nevi, no enlarged superficial lymph nodes, no scleral jaundice. No lip cyanosis (finger O2 sat 96%), slightly congested pharynx, soft neck, distended neck veins. Coarse breath sounds bilaterally, dry/wet rales in lower lungs, few scattered wheezes, heart border enlarged to both sides, distant muffled heart sounds, HR 86 bpm, regular, no pathologic murmurs, no pericardial friction rub or knock, soft/nontender abdomen, no hepatosplenomegaly, negative hepatojugular reflux and shifting dullness, normal bowel sounds, no lower limb edema, normal bilateral dorsalis pedis pulses, no peripheral vascular signs.

**Question 1:** Correct descriptions about this patient's condition are:
A. Can be definitively diagnosed as silicosis
B. Possible respiratory infection
C. Possibly cardiac dyspnea
D. Possible cardiac enlargement
E. Possible pericardial effusion
F. Cardiac disease may be the main current cause

**[Hint]** ① ECG: Sinus rhythm, essentially normal. ② Myocardial markers: CK-MB mass 1.0 ng/ml, TnI 0.001 ng/ml. NT-proBNP: 200.4 pg/ml. ③ Blood routine: WBC 6.4×10⁹/L, N 70.8%, RBC 3.99 T/L, Hb 103 g/L, PLT 319×10⁹/L. ④ Thyroid function, coagulation, urinalysis, stool routine normal. ⑤ Liver/renal function electrolytes: Normal bilirubin, ALT 84 U/L, AST 45 U/L, Total protein 65.4 g/L, Albumin 38 g/L; BUN 5.89 mmol/L, Cr 76.1 µmol/L, Normal uric acid; K⁺ 3.8 mmol/L, other electrolytes normal. Blood glucose/lipids: FBG 5.7 mmol/L, TG 0.76 mmol/L, TC 2.68 mmol/L, HDL-C 0.9 mmol/L, LDL-C 1.19 mmol/L. D-Dimer <0.50 mg/L. ⑥ Chest X-ray: Pulmonary congestion, interstitial edema, suspected bilateral pneumonia, globular heart. ⑦ Echocardiography: Large pericardial effusion (supine: anterior to RV wall ~1.2cm, posterior to LV wall ~3.1cm, lateral to LV wall ~2.9cm), normal remaining cardiac morphology/valve motion. ⑧ Abdominal US: Liver, gallbladder, pancreas, spleen, retroperitoneal great vessels normal, no significant ascites.

**Question 2:** Conclusions from auxiliary tests:
A. Acute myocardial injury
B. Suspected bilateral pneumonia
C. Unlikely dyspnea from pulmonary embolism
D. Presence of cardiac dyspnea
E. Pericarditis likely chronic stage
F. Pericardial effusion compressing bronchi/lungs and pulmonary congestion are main causes of acute dyspnea exacerbation

**[Hint]** ① Lung CT plain: Nodule near descending aorta in left lower lobe, nature indeterminate; Bilateral interstitial lung disease; Multiple low-density nodular opacities in left lung, thickened bilateral interlobar fissures, multiple small nodules in left major fissure, differentiate tumor vs inflammation; Increased/enlarged mediastinal and bilateral axillary lymph nodes; Bilateral pleural effusion, segmental atelectasis in lower lobes; Pericardial effusion. ② Pericardial fluid: Bloody, turbid, Rivalta's test positive, total cells 50,000×10⁶/L, total WBC 1800×10⁶/L. Biochemistry: Total protein 48.3 g/L, Total cholesterol 2.0 mmol/L, LDH 269 U/L, ADA 15 U/L. Pericardial fluid cytology: Many RBCs, some lymphocytes, few suspicious dysplastic cells. No acid-fast bacilli on smear. ③ Sputum: No fungi/acid-fast bacilli on smear, culture normal oral flora. ④ Blood tests for TB antibodies, tumor markers, autoantibodies, virology all negative.

**Question 3:** Causes of pericardial effusion that can be basically ruled out in this patient are:
A. Viral infection
B. Pyogenic infection
C. Connective tissue disease
D. Renal disease
E. Tumor
F. Tuberculous infection

**Question 4:** The patient can be diagnosed with:
A. Acute pericarditis
B. Congestive heart failure
C. Chronic interstitial lung disease
D. Pulmonary infection
E. Large pericardial effusion (likely neoplastic)
F. Pulmonary hypertension

**Question 5:** Treatments for this patient include:
A. Bed rest, oxygen
B. Encourage high-protein, high-calorie, vitamin-rich diet
C. Pericardiocentesis for decompression
D. IV fluoroquinolone (levofloxacin)
E. Antineoplastic chemotherapy
F. Anti-tuberculosis treatment

**【Case 11】**
Male patient, 23 years old. Chest pain, palpitations, fatigue 1 week after a common cold.
Physical examination: BP 110/60mmHg, HR 110 bpm. Auscultation: S1 slightly muffled, no pericardial friction rub. CK-MB 350 U/L. ECG: Sinus tachycardia, frequent ventricular premature beats, mild ST-T changes in precordial leads.

**Question 1:** The most likely diagnosis is:
A. Acute pericarditis
B. Sepsis
C. Acute Keshan disease
D. Viral myocarditis
E. Rheumatic heart disease
F. Acute infective endocarditis

**Question 2:** The "gold standard" examination for definitive diagnosis is:
A. Esophageal pacing
B. Cardiac MRI
C. Coronary CTA
D. Myocardial biopsy
E. Serum pathogen antibody testing
F. Coronary angiography

**Question 3:** If the patient's condition worsens short-term, it may lead to:
A. Acute heart failure
B. Cardiogenic shock
C. Adams-Stokes syndrome
D. Malignant arrhythmia
E. Atrioventricular block
F. Sudden death

**Question 4:** The patient's current treatment includes:
A. Avoid exertion
B. Balanced diet
C. Use beta-blockers
D. Use immunomodulators
E. Appropriately add myocardial metabolism improving drugs
F. Add antiplatelet drugs

**Question 5:** If after 2 days of treatment, the patient develops significant chest tightness, shortness of breath, with restlessness, cold clammy skin, thready pulse. Physical examination: BP 85/45mmHg, HR 140 bpm, SpO₂ 89%. Then, besides routine anti-shock medical management, the correct actions are:
A. Can give human immunoglobulin
B. Can give methylprednisolone pulse therapy
C. Can give antiviral therapy
D. If necessary, can give CRRT/hemofiltration
E. If necessary, can give IABP, LVAD, ECMO mechanical support
F. If necessary, can give endotracheal intubation, mechanical ventilation support

**【Case 12】**
Female patient, 48 years old. Admitted due to exertional dyspnea for 2 years, worsened with fever for 3 weeks. Never received systematic treatment. Penicillin allergy history.
Physical examination: T 38.1℃, P 116 bpm, R 24 bpm, BP 130/62mmHg, HR 96 bpm, regular rhythm, diastolic mid-late rumbling murmur audible at left sternal border. Few moist rales at lung bases. Mild splenomegaly.

**Question 1:** To confirm diagnosis, immediate examinations include:
A. Blood routine
B. Myocardial enzyme studies
C. Electrocardiogram
D. Chest X-ray
E. Transthoracic echocardiography
F. Percutaneous coronary angiography

**Question 2:** If TTE suggests aortic valve leaflets separate >1mm during diastole, diastolic LVOT regurgitant jet visible. For definitive diagnosis, further tests include:
A. Transesophageal echocardiography
B. Dynamic ECG
C. Coronary angiography
D. Serum immunology markers
E. Blood cultures
F. Dynamic BP monitoring

**Question 3:** In the Duke criteria for diagnosing this disease, minor criteria include:
A. Predisposing factors like valvular heart disease, IV drug use
B. Temperature >38℃
C. Vascular phenomena: major arterial emboli, septic pulmonary infarcts, mycotic aneurysm, intracranial hemorrhage, conjunctival hemorrhages, Janeway lesions
D. Immunological phenomena: glomerulonephritis, Osler's nodes, Roth's spots, rheumatoid factor
E. Microbiological evidence: positive blood culture but not meeting major criterion
F. Echocardiogram: consistent with IE but not meeting major criterion

**Question 4:** If TEE shows 4mm vegetation on aortic valve, blood culture pending. Current management includes:
A. Oxygen therapy
B. Use digitalis drugs
C. IV vancomycin drip
D. IM penicillin
E. Oral warfarin
F. Emergency surgical surgery

**Question 5:** Situations requiring consideration for urgent aortic valve replacement surgery in this patient are:
A. Severe aortic regurgitation/obstruction causing refractory pulmonary edema
B. Aorto-cardiac or pericardial fistula formation
C. Vegetation >1cm, recurrent embolic events despite appropriate antibiotics
D. Persistent fever and positive blood culture for 7-10 days or more
E. Infection caused by certain fungi or multidrug-resistant organisms
F. No heart failure, infection controlled fairly well

**【Case 13】**
Male patient, 45 years old, businessman, usually many social engagements. Long-term smoking and diabetes history, family history of coronary heart disease. 5 hours ago, after dinner with alcohol, sudden paroxysmal chest tightness, initially lasting minutes, gradually prolonged to half-hour intervals with relief, now chest tightness gradually worsening, rest no relief, with全身出汗 (profuse sweating).
Physical examination: T 36.2℃, P 114 bpm, R 22 bpm, BP 90/60mmHg. Acute distressed facies, no neck vein distension, clear lung sounds; HR 114 bpm, regular, no murmurs; soft abdomen, liver/spleen not palpable, no lower limb edema, cool extremities. No pathological signs.

**Question 1:** The most likely diagnosis is:
A. Pneumothorax
B. Aortic dissection
C. Pleuritic chest pain
D. Pulmonary embolism
E. Acute coronary syndrome
F. Cardiac neurosis

**Question 2:** Current examinations to choose are:
A. Electrocardiogram
B. Blood routine, ESR, CRP
C. Liver/renal function, electrolytes, lipids, glucose
D. Cardiac necrosis markers
E. Exercise stress test
F. Coronary angiography

**[Hint]** ECG: Significant ST elevation V1-V6 up to 0.2-0.6mV. Combining clinical symptoms and ECG, can rapidly diagnose acute extensive anterior wall ST-elevation myocardial infarction.

**Question 3:** The next step in management should be:
A. Wait for biochemical results before deciding
B. Symptomatic treatment, monitor ECG changes periodically
C. Emergency coronary angiography
D. Volume expansion for shock
E. Implant temporary pacemaker
F. Emergency CABG

**【Case 14】**
Male patient, 65 years old. Admitted for exertional precordial pain for 5 years, worsened with dyspnea for 10 days.
Physical examination: P 90 bpm, BP 105/90mmHg, HR 90 bpm. Few moist rales at lung bases, heart border mildly enlarged to left, systolic ejection murmur audible over aortic area, radiating to carotid artery with thrill.

**Question 1:** The most likely initial diagnosis is:
A. Aortic stenosis
B. Pulmonary stenosis
C. Mitral insufficiency
D. Hypertrophic obstructive cardiomyopathy
E. Mitral stenosis
F. Aortic insufficiency

**Question 2:** Common clinical manifestations of this disease are:
A. Angina pectoris
B. Dyspnea
C. Syncope
D. Palpitations
E. Nausea/Vomiting
F. Abdominal pain/Diarrhea

**Question 3:** Positive signs in such patients include:
A. Weakened peripheral arterial pulses
B. Water-hammer pulse
C. Normal S1 but weakened/absent S2
D. Systolic ejection murmur intensity proportional to disease severity
E. Paradoxical splitting of S2
F. Distant heart sounds

**Question 4:** Inappropriate drug treatment for this patient is:
A. Isosorbide dinitrate
B. Diuretics
C. Digoxin
D. Nifedipine
E. Atenolol
F. Warfarin

**Question 5:** Besides medication, current treatments for this disease also include:
A. PCI
B. CABG
C. AVR (Aortic Valve Replacement)
D. TAVI (Transcatheter Aortic Valve Implantation)
E. CPVI (Circumferential Pulmonary Vein Isolation) (Note: This is for AF, not AS)
F. MVR (Mitral Valve Replacement)

**【Case 15】**
Male patient, 60 years old. Admitted due to chest pain for 1 day. ECG showed ST elevation V1-V5 5mm, diagnosed acute extensive anterior wall MI. On hospital day 3, suddenly developed restlessness, dyspnea, orthopnea, pink frothy sputum.
Physical examination: P 120 bpm, BP 85/60mmHg. Extensive bubbling rales in lungs. Heart border mildly enlarged to left, HR 120 bpm, regular rhythm, S1 slightly weakened, systolic blowing murmur audible at mitral area, liver/spleen not palpable.

**Question 1:** The initial diagnosis should include:
A. Cardiac tamponade
B. Papillary muscle rupture
C. Ventricular aneurysm formation
D. Killip class IV heart failure
E. Acute respiratory failure
F. Cardiac rupture

**Question 2:** If the patient was sent to emergency within 6 hours of chest pain onset, the recommended treatment is:
A. Primary PCI
B. Elective PCI
C. Thrombolytic therapy
D. Facilitated PCI
E. Intensive antithrombotic medication
F. Emergency CABG

**Question 3:** Drugs that can be used in the acute phase are:
A. Dopamine
B. Dobutamine
C. Furosemide
D. Nitroglycerin
E. Doxofylline
F. Metoprolol sustained-release tablets

**Question 4:** Reasonable examinations helpful for differential diagnosis are:
A. Electrocardiogram
B. Chest CT
C. Echocardiography
D. Right heart catheterization
E. Coronary angiography
F. Chest X-ray

**Question 5:** If coronary angiography shows three-vessel disease, SYNTAX score 35, the most reasonable next treatment is:
A. Intra-aortic balloon pump (IABP)
B. Surgical bypass (CABG)
C. PCI
D. IABP + CABG
E. IABP + PCI
F. Medication only

**【Case 16】**
Male child, 9 years old. Admitted due to chest tightness, palpitations for 1 week. Previously healthy.
Physical examination: BP 120/70mmHg, HR 75 bpm. Regular rhythm, thin build, slightly cyanotic lips, coarse breath sounds bilaterally, no rales, grade 3/6 systolic murmur audible at left sternal border 3rd intercostal space.

**Question 1:** Possible diagnoses are:
A. Atrial septal defect
B. Patent ductus arteriosus (PDA)
C. Ventricular septal defect
D. Tetralogy of Fallot
E. Pulmonary hypertension
F. Heart failure

**Question 2:** Required auxiliary examinations are:
A. Chest X-ray
B. Blood routine
C. ECG
D. Cardiac ultrasound
E. Myocardial enzymes
F. Myocardial nuclear imaging
G. Right heart catheterization

**Question 3:** Chest X-ray shows prominent pulmonary artery segment. Blood routine: WBC 10.5×10⁹/L. Cardiac ultrasound shows 5mm muscular VSD, left-to-right shunt. ECG suggests sinus rhythm. The diagnosis is:
A. Atrial septal defect
B. PDA
C. Ventricular septal defect
D. Tetralogy of Fallot
E. Pulmonary hypertension
F. Heart failure

**Question 4:** Based on above results, the next step treatment is:
A. No treatment needed
B. Medical conservative treatment
C. Interventional closure surgery
D. Oxygen therapy
E. Observe for now, consider treatment when older
F. Close monitoring, treat if right-to-left shunt develops
G. Surgical repair

**【Case 17】**
Male patient, 66 years old. Headache, dizziness, palpitations for over 6 years, worsened with blurred vision for 1 week.
Physical examination: BP 180/100mmHg, conscious, clear lung sounds, no rales, HR 90 bpm, irregular rhythm, audible premature beats, no murmurs. Fundus grade III.

**Question 1:** The patient's diagnosis is:
A. Grade 3 hypertension, high risk
B. Grade 2 hypertension, high risk
C. Grade 3 hypertension, very high risk
D. Grade 2 hypertension, medium risk
E. Grade 2 hypertension, very high risk
F. Grade 1 hypertension, very high risk
G. Grade 1 hypertension, high risk

**Question 2:** Diseases requiring differentiation for this condition are:
A. Primary aldosteronism
B. Pheochromocytoma
C. Cushing's syndrome
D. Renovascular hypertension
E. Renal parenchymal hypertension
F. Coarctation of aorta
G. Glaucoma

**Question 3:** Further examinations needed after admission include:
A. Electrocardiogram
B. Echocardiography
C. Chest PA X-ray
D. Blood lipids
E. Myocardial nuclear imaging
F. 24-hour dynamic ECG monitoring
G. Coronary angiography

**[Hint]** ECG suggests LVH, ventricular premature beats; Echo: IVS thickness 15mm; Chest PA: Aortic configuration, cardiothoracic ratio 0.6; Holter: Ventricular premature beats, total 872, Lipids: LDL 4.2 mmol/L.

**Question 4:** The next step in treatment is:
A. Lifestyle modification
B. Monitor BP and risk factors for 3-6 months, no treatment yet
C. Rapid IV antihypertensive therapy
D. Statin therapy
E. Beta-blockers
F. Calcium channel blockers
G. ACE inhibitors

**Question 5:** If untreated, complications that may develop as the disease progresses include:
A. Hypertensive crisis
B. Hypertensive encephalopathy
C. Heart failure
D. Chronic renal insufficiency
E. Aortic dissection
F. Cerebral hemorrhage
G. Ventricular aneurysm

**【Case 18】**
Male patient, 68 years old. Admitted due to sudden severe chest pain for 2 hours. Past hypertension history, not taken seriously.
Physical examination: BP 180/100mmHg, P 80 bpm, coarse breath sounds bilaterally, no wet rales, HR 80 bpm, regular, no murmurs.

**Question 1:** The considered diagnoses are:
A. Aortic dissection
B. Pulmonary embolism
C. Acute myocardial infarction
D. Pneumothorax
E. Acute pericarditis
F. Pleurisy
G. Intercostal neuralgia

**Question 2:** For definitive diagnosis, further examinations are:
A. Blood routine
B. ECG
C. Chest X-ray
D. Cardiac ultrasound
E. Myocardial enzymes
F. Cardiac troponin I (cTnI)
G. D-dimer
H. Blood gas analysis

**[Hint]** Blood routine: WBC 10.0×10⁹/L, ECG: Sinus rhythm, cTnI <0.1 ng/ml, D-dimer <0.3 mg/L FEU (or µg/L, assumed low), Chest X-ray: Widened mediastinum.

**Question 3:** The most likely diagnosis is:
A. Acute myocardial infarction
B. Aortic dissection
C. Pulmonary embolism
D. Pneumothorax
E. Pericarditis
F. Pericardial effusion

**Question 4:** For further confirmation, the next examination needed is:
A. Aortic enhanced CT
B. Aortic MRI
C. Aortic Doppler ultrasound
D. Myocardial nuclear imaging
E. Chest fluoroscopy
F. Coronary angiography

**Question 5:** The next step in treatment to first consider is:
A. Oxygen and sedation/analgesia
B. Aspirin
C. Cardiotonic agents
D. ACEI or ARB
E. Statins
F. Diuretics
G. Beta-blockers
H. Calcium channel blockers
I. Surgical treatment

**【Case 19】**
Male patient, 25 years old. Exertional shortness of breath for 2 years, admitted with dizziness. Physical examination: Grade 3/6 systolic ejection murmur audible at left sternal border 3rd-4th intercostal spaces, no thrill, grade 3/6 systolic blowing murmur audible at apex, no lower limb edema.

**Question 1:** This patient's diagnosis could be:
A. Dilated cardiomyopathy
B. Ventricular septal defect
C. Patent ductus arteriosus
D. Pulmonary stenosis
E. Obstructive hypertrophic cardiomyopathy
F. Rheumatic heart disease, aortic stenosis
G. Coronary heart disease
H. Pulmonary heart disease

**Question 2:** Examinations the patient should undergo are:
A. Echocardiography
B. Cardiac function tests
C. Phonocardiography
D. Electrocardiogram
E. Chest X-ray
F. Cardiac CT
G. Exercise testing
H. Coronary angiography

**[Hint]** Echocardiography: IVS 20mm, LV posterior wall 13mm, SAM sign visible, CDFI shows systolic mosaic flow jet in LVOT, systolic mosaic flow jet regurgitating to LA visible at mitral orifice.

**Question 3:** The patient can now be definitively diagnosed with:
A. Dilated cardiomyopathy
B. Obstructive hypertrophic cardiomyopathy
C. Ventricular septal defect
D. Patent ductus arteriosus
E. Pulmonary stenosis
F. Rheumatic heart disease, aortic stenosis
G. Rheumatic heart disease, mitral insufficiency
H. Coronary heart disease

**Question 4:** Management for this patient is:
A. Beta-blockers
B. Verapamil
C. Digitalis
D. Diuretics
E. Nitroglycerin
F. Septal ablation
G. Cardiac pacing
H. Surgical septal myectomy

**Question 5:** Features *not* characteristic of obstructive HOCM are:
A. Septal hypertrophy
B. Multifocal ventricular premature beats
C. Post-stenotic dilation of ascending aorta
D. Ventricular hypertrophy
E. Diastolic gallop
F. Rough systolic murmur audible over aortic area, radiating to neck
G. Rumbling diastolic murmur at apex
H. Systolic ejection murmur at left sternal border 3rd-4th ICS

**Question 6:** Incorrect descriptions about obstructive HOCM are:
A. Markedly enlarged left ventricle
B. Markedly hypertrophied left ventricle
C. Systolic murmur audible at right sternal border
D. Echocardiography shows SAM sign
E. Beta-blockers can be first-line drugs
F. Often can cause sudden death
G. Can use nitroglycerin
H. Can use verapamil

**Question 7:** The reason for audible paradoxical splitting of S2 in obstructive HOCM is:
A. Obstruction to left ventricular ejection
B. Hyperdynamic LV contraction
C. Impaired LV filling
D. Pressure gradient across outflow tract
E. Small end-systolic volume
F. Left ventricular dilation
G. Prolonged LV ejection time
H. Shortened RV ejection time

**Question 8:** Factors that can intensify the murmur in obstructive HOCM are:
A. Valsalva maneuver
B. Bradycardia
C. Nitroglycerin
D. Digitalis
E. Beta-blockers
F. Diuretics
G. Isoproterenol
H. Tachycardia

**【Case 20】**
Male patient, 60 years old. Dinner at 18:00, drank about 250ml of 39%白酒 (baijiu) (usually can drink 400ml of same without drunk), paroxysmal abdominal pain half hour later, with nausea, came to emergency at 20:00. Hypertension history 8 years, usually takes nifedipine 10mg, 3 times/day, BP fluctuates (150-180)/(90-100)mmHg. Usually, no chest tightness or angina.
Physical examination: T 36.8℃, P 90 bpm, R 22 bpm, BP 150/75mmHg. Regular rhythm, normal lung sounds, soft abdomen, tenderness at xiphoid (±), no abdominal muscle rigidity, normal limb movement.

**Question 1:** Emergency diagnosis considerations are:
A. Acute gastritis
B. Acute cholecystitis
C. Acute pancreatitis
D. Acute gastroenteritis
E. Angina pectoris
F. Hypertension
G. Acute myocardial infarction
H. Cerebrovascular accident
I. Gastric perforation
J. Acute alcohol intoxication

**Question 2:** Emergency examinations should mainly be:
A. Blood routine
B. Bedside ECG
C. Abdominal X-ray plain film
D. Head CT
E. Stool routine and occult blood
F. Serum amylase
G. Gastroscopy
H. Serum myocardial enzymes

**[Hint]** Further examination: Conscious, soft neck no resistance, normal heart/lung sounds, soft abdomen, no obvious epigastric tenderness/rebound, no mass, Murphy's sign negative, liver dullness present, normal limb movement, pathological signs negative, amylase normal. WBC 11×10⁹/L, N 0.78, CK 100 U/L.

**Question 3:** Diseases that can be ruled out at this point are:
A. Acute gastritis
B. Acute cholecystitis
C. Acute pancreatitis
D. Acute myocardial infarction
E. Angina pectoris
F. Cerebrovascular accident
G. Gastric perforation

**[Hint]** Bedside ECG: ST elevation II, III, aVF convex upward 1.1mV, ST depression V1, V2 0.075mV. Patient developed crushing substernal pain.

**Question 4:** The patient's definitive diagnoses are:
A. Acute gastritis
B. Acute cholecystitis
C. Acute inferior wall myocardial infarction
D. Variant angina
E. Hypertension
F. Acute pericarditis
G. Biliary-cardiac syndrome

**[Hint]** Patient still has mild xiphoid pain, relieved by local massage, Murphy's sign negative, confirmed acute inferior wall MI.

**Question 5:** Emergency measures at this time should include:
A. Absolute bed rest
B. Continuous oxygen
C. Morphine 3mg IM
D. Continuous cardiac monitoring
E. Continuous IV nitroglycerin drip
F. Regular turning
G. IV furosemide to promote alcohol excretion
H. Polarizing solution (Glucose-Insulin-Potassium)
I. Beta-blocker application
J. Use cedilanid or digoxin

**[Hint]** After comprehensive management, chest pain persisted for nearly 5 hours without complete relief.

**Question 6:** Planned IV thrombolysis. General indications for thrombolysis are:
A. Age 60 years
B. Onset 5 hours
C. Acute inferior wall MI
D. Has hypertension
E. After alcohol consumption
F. No arrhythmia
G. No heart failure

**[Hint]** After IV urokinase 1.2 million units, pain resolved quickly.

**Question 7:** Clinical indicators of coronary reperfusion are:
A. Rapid pain relief
B. ECG returns to normal
C. Appearance of Q waves
D. ST segment resolution >50%
E. Early peak of myocardial enzymes
F. Patent vessel on coronary angiography
G. Myocardial enzymes rapidly normalize
H. Appearance of frequent VPBs or VT

**[Hint]** After IV urokinase 1.2 million U, developed frequent VPBs and paroxysmal VT.

**Question 8:** Key management points now are:
A. Lidocaine 80mg IV bolus, then lidocaine IV drip at 2 mg/min
B. IV potassium supplementation
C. Continue observation, no action yet
D. Increase nitroglycerin drip dose
E. Use sedatives
F. Electrical cardioversion
G. Stop anticoagulants
H. Bedside late potential test
I. Check electrolytes

**[Hint]** Due to slightly large IV fluid volume and fast drip rate, plus poor dietary control, on hospital day 3, developed rapid atrial fibrillation, patient complained of palpitations and chest tightness. After slowing infusion, IV furosemide push, and using cedilanid/digoxin for rate control, ventricular rate slowed. After 5 days of treatment, AF still not converted, planned electrical cardioversion.

**Question 9:** Correct procedures for electrical cardioversion are:
A. Use AC cardioversion
B. Use DC cardioversion
C. Stop digoxin 1 day before cardioversion
D. No need to stop digoxin before cardioversion
E. Use energy 50-100 J
F. Use energy 300-400 J
G. Use synchronized cardioversion
H. Use unsynchronized cardioversion
I. IV diazepam push until patient asleep then cardiovert
J. No need for diazepam, cardiovert while conscious
